# Anle138b ameliorates pathological phenotypes in mouse and cellular models of Huntington’s disease

**DOI:** 10.1038/s44321-026-00459-9

**Published:** 2026-06-26

**Authors:** Miguel da Silva Padilha, Seda Koyuncu, Evangeline Chabanis, Sergey Ryazanov, Andrei Leonov, David Vilchez, Rüdiger Klein, Armin Giese, Christian Griesinger, Irina Dudanova

**Affiliations:** 1 Center for Anatomy, Faculty of Medicine and University Hospital Cologne, University of Cologne, Cologne, Germany; 2 Department of Molecules – Signaling – Development, Max Planck Institute for Biological Intelligence, Martinsried, Germany; 3 Cologne Excellence Cluster on Cellular Stress Responses in Aging-Associated Diseases (CECAD), University of Cologne, Cologne, Germany; 4 Institute for Integrated Stress Response Signaling, Faculty of Medicine, University Hospital Cologne, Cologne, Germany; 5 Department of NMR Based Structural Biology, Max Planck Institute of Multidisciplinary Sciences, Göttingen, Germany; 6 Center for Molecular Medicine Cologne (CMMC), Faculty of Medicine and University Hospital Cologne, University of Cologne, Cologne, Germany; 7 MODAG GmbH, Wendelsheim, Germany; 8 Cluster of Excellence “Multiscale Bioimaging: From Molecular Machines to Networks of Excitable Cells” (MBExC), University of Göttingen, Göttingen, Germany; 9 Institute of Anatomy and Cell Biology, University of Würzburg, Würzburg, Germany

**Keywords:** Neuroscience

## Abstract

Huntington’s disease (HD) is a hereditary movement disorder caused by a CAG repeat expansion in the *huntingtin* gene. HD is characterized by deposition of mutant huntingtin (mHTT) aggregates, and by severe neurodegeneration of the basal ganglia and neocortex. No cure is currently available, and new treatment options are urgently needed. Here, we show that the oligomer modifying molecule anle138b (INN: emrusolmin) improves multiple disease phenotypes in cell culture and in two mouse models of HD. Application of anle138b reduced mHTT aggregate formation and ameliorated neurotoxicity in primary neurons. Oral administration of anle138b delayed deposition of mHTT inclusions, reduced brain atrophy, mitigated neuroinflammation and transcriptional alterations, improved motor function and extended life span in HD mice. Downregulation of striatal markers and synapse loss in striatal spiny projection neurons were also partially rescued. No adverse effects of anle138b were observed in wildtype animals. Moreover, anle138b markedly decreased mHTT aggregation in human neural precursor cells differentiated from HD patient-derived induced pluripotent stem cells (iPSCs). Altogether these results illustrate the potential of anle138b as a disease-modifying treatment for HD.

## Synopsis

The small molecule anle138b (Emrusolmin) decreases mutant Huntingtin (mHTT) aggregate load and has beneficial effects on disease-related phenotypes in cellular and animal models of Huntington’s disease (HD).

- Anle138b ameliorated toxicity of mHTT and reduced the frequency of mHTT inclusions in murine primary neurons.
- Oral administration of anle138b improved neuropathological and molecular disease hallmarks in two different HD mouse models.
- Anle138b treatment rescued motor dysfunction and prolonged life span of HD mice.
- Anle138b reduced mHTT aggregation in human HD neural precursor cells.

## Abstract (web summary)

The small molecule anle138b (Emrusolmin) decreases mutant Huntingtin (mHTT) aggregate load and has beneficial effects on disease-related phenotypes in cellular and animal models of Huntington’s disease (HD).

## Graphical abstract

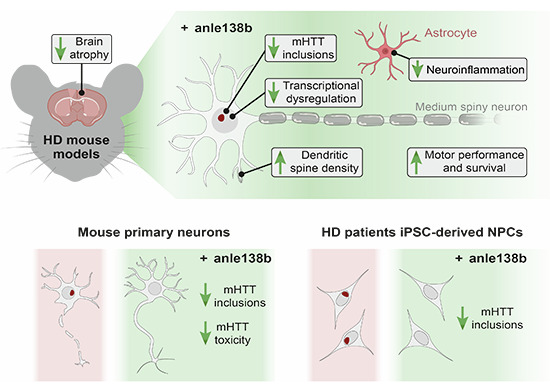

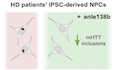

The paper explained **Problem**

Huntington’s disease (HD) is a devastating hereditary movement disorder without a current cure. The disease is caused by a CAG repeat expansion in the Huntingtin (*HTT*) gene, which makes the HTT protein aggregation-prone and leads to deposition of mutant HTT (mHTT) inclusions in the brain. HD causes severe neurodegeneration and motor impairments.

The oligomer modulator anle138b has been shown to reduce protein aggregates and ameliorate symptoms in models of other neurodegenerative proteinopathies. Moreover, the phase 1 trial revealed good safety and tolerability profiles of the compound. However, the therapeutic potential of anle138b in HD remains unexplored.

**Results**

This study demonstrates the beneficial effects of anle138b in cellular and mouse models of HD. In HD mice, administration of anle138b reduced mHTT aggregate load, mitigated brain atrophy, neuroinflammation, synapse loss and transcriptional alterations in the brain. Furthermore, anle138b ameliorated motor defects and modestly extended the life span of HD mice. The compound did not have any adverse effects in healthy animals. Anle138b also decreased mHTT aggregation in neural precursor cells differentiated from human HD-patient-derived iPSCs, validating the efficacy of the compound in a human model with endogenous mHTT expression.

**Impact**

These findings highlight the promise of anle138b as a disease-modifying therapy for HD. As CAG repeat disorders share common mechanisms, anle138b might also provide therapeutic benefit in disorders beyond HD.

## Introduction

Huntington’s disease (HD) is a hereditary brain disorder characterized by severe neurodegeneration, which affects in particular the basal ganglia and the neocortex (Waldvogel et al, 2015). HD manifests with motor defects, including involuntary movements (chorea) at early stages and akinesia at later stages, as well as psychiatric symptoms and cognitive impairments (Tabrizi et al, 2020). The cause of the disease is a CAG repeat expansion in exon1 of the *huntingtin* (*HTT*) gene (The Huntington’s Disease Collaborative Research Group, 1993). The mutation is translated into a pathologically elongated polyglutamine (polyQ) tract in the HTT protein, resulting in its misfolding, aggregation, and deposition of mutant HTT (mHTT) inclusions in the brain (DiFiglia et al, 1997). HD leads to neuronal damage at least partially through the toxic gain-of-function of the aggregation-prone mHTT, in particular in the form of toxic oligomeric species. mHTT aggregation compromises cellular fitness through a variety of mechanisms, including disturbance of protein homeostasis (proteostasis) (Labbadia and Morimoto, 2013; Miller et al, 2011; Saudou and Humbert, 2016; Tabrizi et al, 2020). Despite encouraging recent progress in the development of HTT-lowering therapies (Farag et al, 2026), no cure for HD is yet available, and there is a great need for additional treatment options. Reducing mHTT aggregation might be a promising disease-modifying strategy.

The diphenylpyrazole compound anle138b [3-(1,3-benzodioxol-5-yl)-5-(3-bromophenyl)-1*H*-pyrazole] (INN: emrusolmin) was identified in a screen for small molecules with prion aggregation-inhibiting activity (Wagner et al, 2013). Anle138b was shown to directly interact with the oligomeric species of disease-related aggregating proteins such as prion protein, α-synuclein, amyloid-beta (Aβ), tau and human islet amyloid polypeptide, inhibiting formation of aggregates and reducing their toxicity (Albariqi et al, 2024; Heras-Garvin et al, 2019; Martinez Hernandez et al, 2018; Wagner et al, 2015; Wagner et al, 2013; Wegrzynowicz et al, 2019). The compound proved to have beneficial effects in mouse models of different neurodegenerative proteinopathies, including prion disease, synucleinopathy, tauopathy and Aβ deposition (Brendel et al, 2019; Heras-Garvin et al, 2019; Levin et al, 2014; Martinez Hernandez et al, 2018; Wagner et al, 2015; Wagner et al, 2013). Anle138b is orally bioavailable, has suitable pharmacokinetic properties and efficiently penetrates the blood-brain barrier (Wagner et al, 2013). Importantly, a phase 1 clinical trial for anle138b demonstrated good safety and tolerability profiles of the compound at exposure levels exceeding those required for full efficacy in mouse models (Levin et al, 2022). A phase 2 trial in multiple system atrophy patients is currently ongoing (NCT06568237). However, the therapeutic potential of anle138b in the context of HD has not been explored to date.

Here, we show that anle138b reduces mHTT aggregation in cultured primary neurons, two complementary mouse models of HD (the early-onset transgenic R6/2 model with a severe phenotype and the late-onset zQ175DN knock-in model that is genetically similar to patients), as well as in human neural precursor cells (NPCs) differentiated from HD patients-derived induced pluripotent stem cells (iPSCs). Furthermore, anle138b mitigates neuropathological disease signatures, significantly improves motor performance, and extends the life span of HD mice. These findings highlight the potential of anle138b as a treatment for HD.

## Results

### Anle138b reduces mHTT toxicity and aggregation in primary neurons

**Figure 1:**
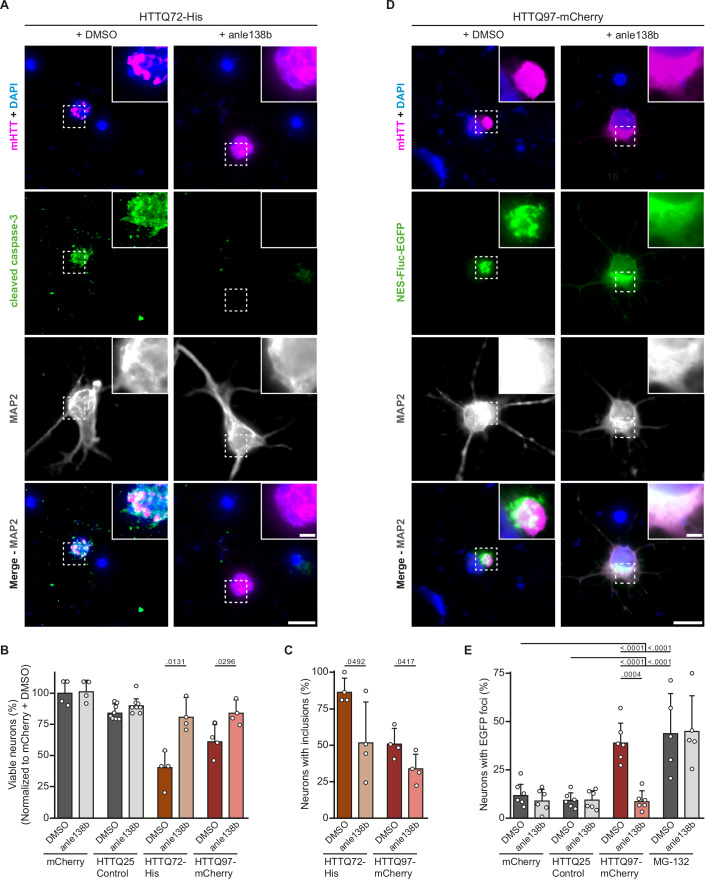
Anle138b reduces mHTT toxicity and improves proteostasis in primary neurons. (**A**) Representative images of day in vitro (DIV) 7 + 2 cortical neurons transfected with HTT-Exon1-Q72-His and treated with vehicle (DMSO, left) or 7 µM anle138b (right). Neurons were stained for cleaved caspase-3 and neuronal marker MAP2, and mHTT was detected by immunostaining against the His-tag. Nuclei were labeled with DAPI. Insets should higher magnification of the areas delineated by the dashed boxes. (**B**) Quantification of the percentage of viable transfected neurons, normalized to mCherry expressing cells treated with DMSO. Unpaired two-tailed *t-*test. *n* = 4 independent experiments. (**C**) Quantification of the fraction of neurons with mHTT inclusion bodies. Unpaired two-tailed *t-*test. *n* = 4 independent experiments. (**D**) Representative images of DIV 7 + 2 cortical neurons co-transfected with HTTQ97-mCherry and NES-Fluc-EGFP, treated with vehicle (DMSO, left) or 7 µM anle138b (right). Neurons were stained for MAP2, mHTT was detected by immunostaining against mCherry, and NES-Fluc-EGFP was detected by EGFP fluorescence. Nuclei were labeled with DAPI. Insets show higher magnifications of the areas delineated by the dashed boxes. (**E**) Quantification of the fraction of double-transfected neurons with EGFP foci. Two-way ANOVA with Bonferroni’s multiple comparison test: Treatment, ****p < 0.0001; Construct, ****p < 0.0001; treatment ×  construct ***p = 0.001. *n* = 5 independent experiments for MG-132 and 6 independent experiments for all other conditions. Data were presented as mean ± SD. *p* values for significant pairwise comparisons are indicated on the graphs. Scale bar in (**A**, **D**), 10 µm; insets, 2 µm. Source data are available online for this figure.

**Figure EV1:**
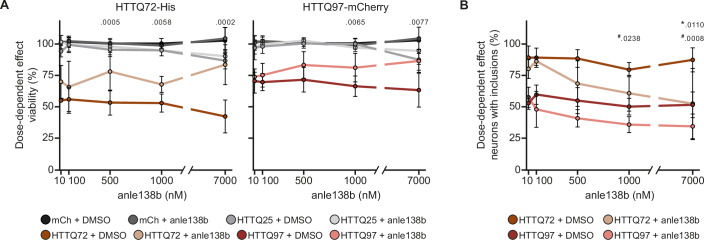
Dose-dependent effect of anle138b in primary neurons. (**A**) Dose-dependent effect of anle138b on the percentage of viable transfected neurons expressing HTTQ72-His (left) or HTTQ97-mCherry (right). Repeated measures ANOVA with Tukey’s multiple comparison test. (**B**) Dose-dependent effect of anle138b on the fraction of neurons with mHTT inclusion bodies. Repeated measures ANOVA with Tukey’s multiple comparison test. Data presented as mean ± SD. *p* values for significant pairwise comparisons are indicated on the graphs: # Difference between the “HTTQ72-His” conditions; * difference between the “HTTQ97-mCherry” conditions. *n* = 4 independent experiments. Source data are available online for this figure

To assess the effects of anle138b in the context of HD, we first employed a cellular model system and assessed mHTT-dependent phenotypes in primary neuronal cultures isolated from mouse embryos. The neurons were transfected with two versions of mHTT-exon 1, which differ in their polyQ length and tags: HTTQ72-His and HTTQ97-mCherry. We have shown in previous studies that these versions reproduce two characteristic locations of mHTT inclusion bodies: HTTQ72-His forms inclusions primarily in the nucleus, while inclusions of HTTQ97-mCherry are found mostly in the cytoplasm (Blumenstock et al, 2021; Voelkl et al, 2023). Immunostaining for the apoptotic marker cleaved caspase-3 was used to quantify neuronal cell death (Fig. 1A). Expression of both versions of mHTT significantly impaired neuronal survival (Fig. 1B), whereas the respective normal polyQ-length constructs (HTTQ25-His and HTTQ25-mCherry) did not cause neurotoxicity and were pooled together as controls. Addition of anle138b to the culture medium resulted in a significant rescue of viability of both HTTQ72-His and HTTQ97-mCherry expressing neurons (Fig. 1B). In addition, anle138b reduced the fraction of neurons bearing mHTT inclusions for both versions of mHTT (Fig. 1C). Experiments with different doses of anle138b in the culture medium demonstrated that the compound had a beneficial effect on neuronal survival already starting from the concentration of 500 nM and on mHTT inclusions from the concentration of 1 µM (Fig. EV1A,B). Of note, these concentrations are lower than the concentrations of anle138b reached in humans in the phase 1 trial (Levin et al, 2022).

To test whether the reduction in mHTT inclusion bodies is accompanied by improved neuronal proteostasis, we used a proteostasis reporter consisting of EGFP-fused firefly luciferase (Fluc-EGFP), which reveals proteostasis defects by forming EGFP foci in the cells (Gupta et al, 2011). We have previously shown that cytoplasmically targeted Fluc-EGFP (NES-Fluc-EGFP) reliably detects impairment in cytoplasmic proteostasis induced by HTTQ97-mCherry (Blumenstock et al, 2021). We therefore co-expressed NES-Fluc-EGFP and HTTQ97-mCherry in primary neurons and quantified EGFP foci upon treatment with anle138b or vehicle control (Fig. 1D). As a positive control, Fluc-EGFP transfected neurons were treated with the proteasome inhibitor MG-132, which readily induces Fluc-EGFP reaction. Consistent with our previous findings (Blumenstock et al, 2021), both HTTQ97-mCherry expression and proteasome inhibition, but not control HTTQ25-mCherry, caused a significant increase in the fraction of neurons with EGFP foci, indicative of impaired proteostasis. This impairment was completely rescued by anle138b application in the case of mHTT, but not MG-132 (Fig. 1E), suggesting that anle138b specifically rectifies proteostasis defects caused by aggregation of mHTT. Taken together, these results indicate that anle138b reduces aggregation of mHTT and prevents mHTT-dependent proteostasis failure and cell death in primary neurons.

### Anle138b improves motor function and extends life span in R6/2 mice

**Figure 2:**
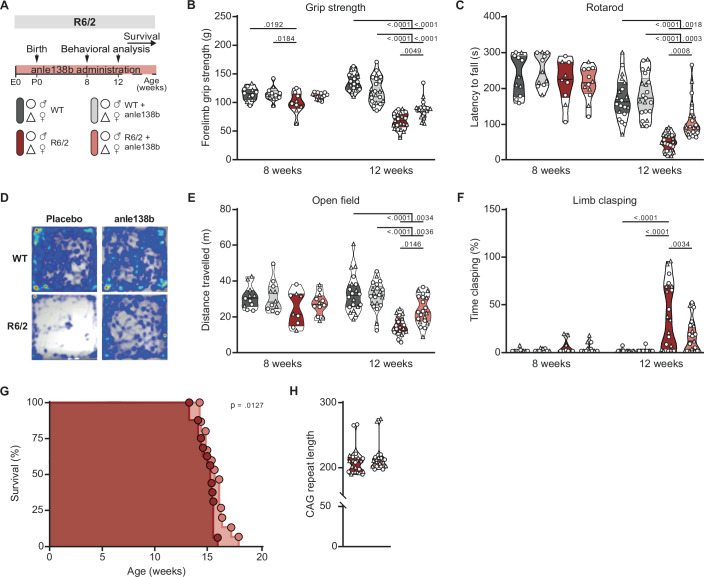
Anle138b improves motor performance and extends life span in R6/2 mice. (**A**) Experimental timeline for the R6/2 mice. (**B**) Forelimb grip strength. Two-way ANOVA with Bonferroni’s multiple comparison test, per age group. ANOVA for the 8 weeks’ time point: Treatment, *p* = 0.0801; Genotype, **p = 0.0073; Treatment × Genotype, *p* = 0.0839. ANOVA for the 12 weeks’ time point: Treatment, *p* = 0.3747; Genotype, ****p < 0.0001; Treatment × Genotype, ****p < 0.0001. (**C**) Latency to fall from the rotarod. Two-way ANOVA with Bonferroni’s multiple comparison test, per age group. 8 weeks: not significant; 12 weeks: Treatment, **p = 0.0023; Genotype, ****p < 0.0001; Treatment × Genotype, *p = 0.0141. (**D**) Heatmap of the open field arena depicting preferred occupied locations, from less (dark blue) to more (red) time spent in the same location. (**E**) Distance traveled in the open field arena. Two-way ANOVA with Bonferroni’s multiple comparison test, per age group. 8 weeks: not significant; 12 weeks: Treatment, *p = 0.0342; Genotype, ****p < 0.0001; Treatment x Genotype, **p = 0.0053. (**F**) Fraction of time spent clasping. Two-way ANOVA with Bonferroni’s multiple comparison test, per age group. 8 weeks: not significant; 12 weeks: Treatment, *p = 0.0131; Genotype, ****p < 0.0001; Treatment × Genotype, *p = 0.0140. (**G**) Kaplan–Meier survival curve. Log-rank test. For all behavioral analyses in (**B**–**F**), *n* = 9 – 12 (8 weeks) or 19–22 mice (12 weeks) per genotype and treatment group. (**H**) CAG repeat length in 13-week-old R6/2 mice. Unpaired two-tailed *t*-test, not significant. *n* = 21–22 mice per group. Males and females are represented by circles and triangles, respectively. Data presented as violin plots with median and interquartile ranges. *p* values for significant pairwise comparisons are indicated on the graphs. Source data are available online for this figure.

To explore the therapeutic potential of anle138b in vivo, we first used the R6/2 mouse line, which is a transgenic fragment model of HD that expresses exon1 of human *HTT* with an expanded CAG tract under the human *HTT* promoter (Mangiarini et al, 1996). The R6/2 model is well-characterized, has a rapid disease progression, clear histological and behavioral phenotypes and a shortened life span (Burgold et al, 2019; Carter et al, 1999; Mangiarini et al, 1996). Non-transgenic littermates were used as controls throughout the study. Since HD is a monogenic disorder, and gene expansion carriers can be identified at an early age or even prenatally through genetic testing, we treated the mice from conception in order to achieve the maximal possible effect of anle138b. The compound was administered in chow at a concentration of 2 g/kg, and placebo food with the same composition but without anle138b served as control. Mice were tested in a battery of assays for motor behavior at the age of 8 weeks (around the onset of motor impairments) and 12 weeks (advanced stage). Some of the animals were sacrificed after the behavioral tests, and brains were taken out for histological and biochemical analyses, while the rest of the mice were used to measure life span (Fig. 2A).

Anle138b treatment did not change the litter size (Appendix Fig. S1A) or the Mendelian distribution of genotypes, suggesting that it does not cause adverse effects on prenatal development. Placebo-treated R6/2 mice showed diminished forelimb strength in the grip strength test starting from 8 weeks. At 12 weeks, they also exhibited impaired coordination on the rotarod and reduced locomotion in the open field. In addition, when suspended by the tail, the mice clasped their limbs, a characteristic phenotype of HD animals (Fig. 2B–F). All these motor defects were significantly mitigated in anle138b-treated R6/2 mice. The compound did not have an effect on the body weight of the mice (Appendix Fig. S1B). Of note, no significant effect of anle138b on wild-type control littermates was observed in any of the tests (Fig. 2B–F; Appendix Fig. S1B). In addition, administration of anle138b modestly prolonged the life span of R6/2 mice by 5 days on average (Fig. 2G). Analyses of the mice separated by sex showed that both the degree of motor defects and the magnitude of their rescue by anle138b were comparable between male and female R6/2 mice (Appendix Fig. S2A–H). In contrast, the beneficial effect of anle138b on survival was due to a significantly longer life span of female mice, while the life span of the male mice was not changed (Appendix Fig. S1C,D). The length of CAG repeats was not different between anle138b- and placebo-treated R6/2 animals (Fig. 2H). Altogether, these findings demonstrate that oral administration of anle138b ameliorates HD-related neurological phenotypes and increases the life span of R6/2 animals.

### Anle138b mitigates brain atrophy and astrogliosis in R6/2 mice

**Figure 3:**
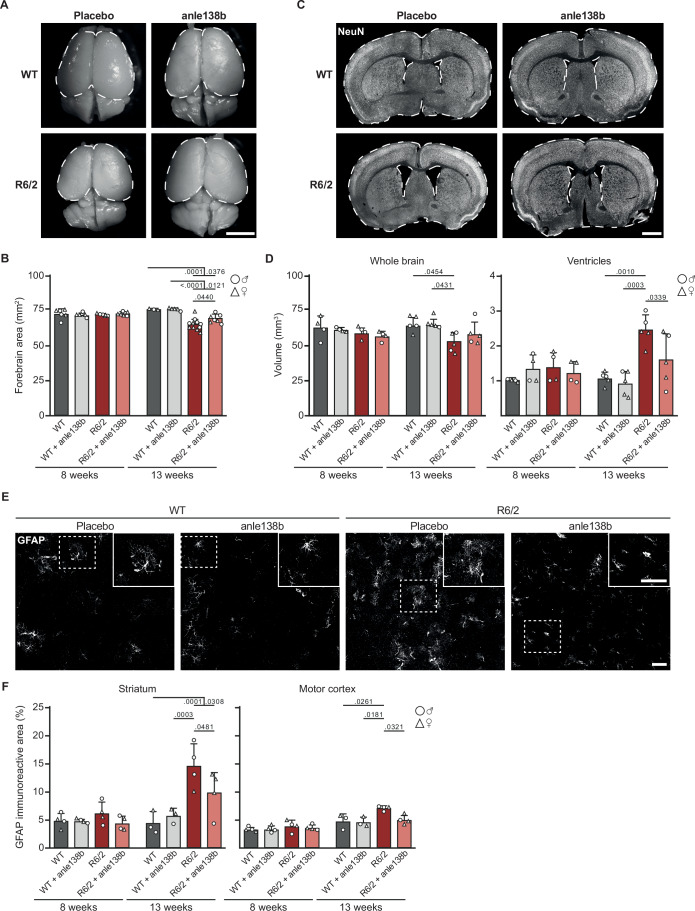
Treatment with anle138b ameliorates brain atrophy and decreases astrogliosis. (**A**) Representative whole brains of 13-week-old wildtype (WT) and R6/2 mice treated with placebo or anle138b. Dashed white lines outline the forebrain. (**B**) Quantification of whole forebrain area in 8 and 13-week-old WT and R6/2 mice. Two-way ANOVA with Bonferroni’s multiple comparison test, per age group. Eight weeks: not significant; 13 weeks: Treatment, *p* = 0.1260; Genotype, ****p < 0.0001; Treatment × Genotype, *p* = 0.1089. *n* = 6 (8 weeks) or 3–11 mice (13 weeks) per group. (**C**) Representative coronal brain sections stained with NeuN from 13-week-old WT and R6/2 mice treated with placebo or anle138b. Dashed white lines outline the whole brain and the ventricles. (**D**) Whole brain and ventricle volume quantification in 8- and 13-week-old WT and R6/2 mice. Two-way ANOVA with Bonferroni’s multiple comparison test, per age group and brain region. Whole brain, 8 weeks: not significant; 13 weeks: Treatment, *p* = 0.3006; Genotype, **p = 0.0047; Treatment × Genotype, *p* = 0.3168. Ventricle volume, 8 weeks: not significant; 13 weeks: Treatment, **p = 0.0052; Genotype, ****p < 0.0001; Treatment × Genotype, *p* = 0.1378. *n* = 4 (8 weeks) or 5 mice (13 weeks) per genotype and treatment group. (**E**) Representative GFAP astrocyte staining in the dorsal striatum of 13-week-old WT and R6/2 mice treated with placebo or anle138b. Insets in the top right corner show higher magnification of the areas delineated by the dashed white boxes. (**F**) Fraction of GFAP immunopositive area in the striatum (left) and motor cortex (right) of 8- and 13-week-old WT and R6/2 mice. Two-way ANOVA with Bonferroni’s multiple comparison test, per age group and brain region. 8 weeks: not significant; Striatum at 13 weeks: Treatment, ***p = 0.0003; Genotype, ***p = 0.0003; Treatment × Genotype, **p = 0.0041. Motor cortex at 13 weeks: Treatment, *p = 0.0328; Genotype, *p = 0.0128; Treatment × Genotype, *p* = 0.0561. *n* = 4 (8 weeks) or 3–4 mice (13 weeks). Data presented as mean ± SD. *p* values for significant pairwise comparisons are indicated on the graphs. Scale bars: (**A**) 5 mm; (**C**) 1 mm; (**E**) 100 µm. Source data are available online for this figure.

To investigate the impact of anle138b treatment on the neuropathological hallmarks of HD, we performed histological analysis of the R6/2 mouse brains at 8 and 13 weeks of age. Placebo-treated R6/2 mice had a significantly smaller forebrain at 13 weeks, a defect that was partially rescued by anle138b (Fig. 3A,B). To assess brain atrophy in more detail, we performed stereological analyses of serial coronal brain sections. The estimated total volume of the brain was significantly smaller in placebo-treated R6/2 mice compared to littermate controls, whereas no significant difference was detected between anle138b-treated R6/2 animals and controls (Fig. 3C,D). Moreover, placebo-treated R6/2 mice showed atrophy of the striatum and motor cortex, as well as a pronounced enlargement of the brain ventricles. While we did not detect a significant effect of anle138b on the size of the striatum or motor cortex (Appendix Fig. S3A), the ventricle enlargement was partially rescued by anle138b administration (Fig. 3C,D).

**Figure EV2:**
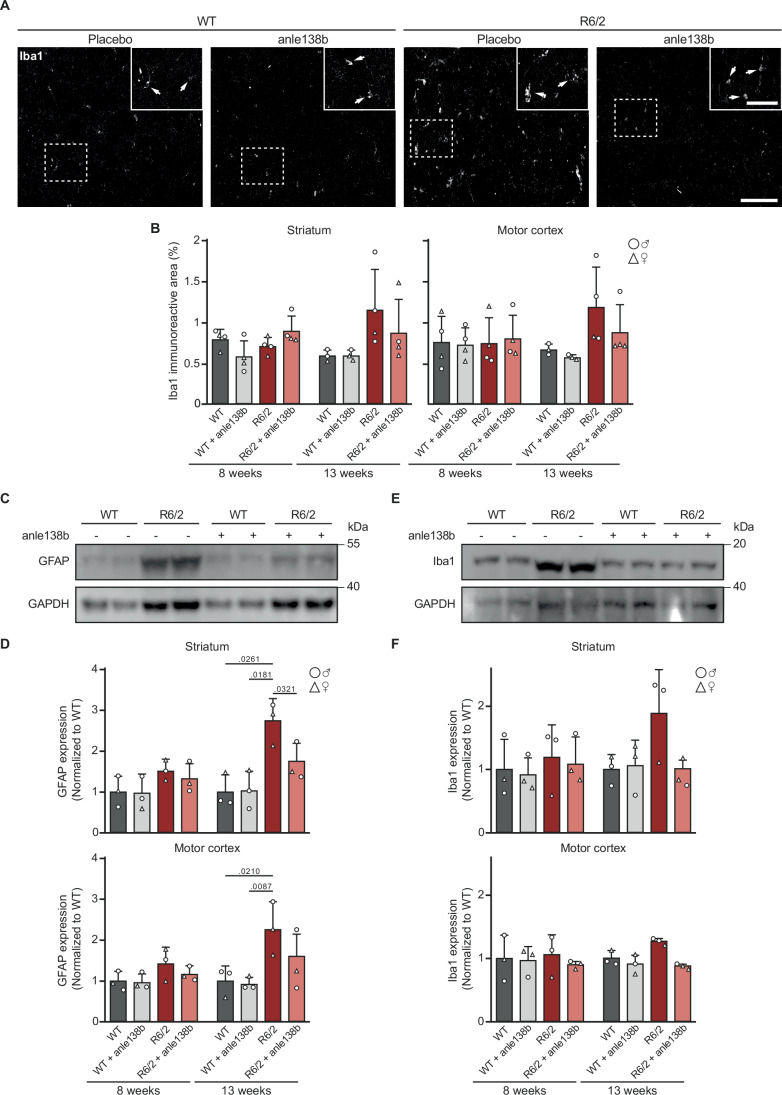
Microgliosis and astrogliosis in anle138b-treated R6/2 mice. (**A**) Representative images of Iba1 immunostaining in the dorsal striatum of 13-week-old WT and R6/2 mice treated with placebo or anle138b. Insets show higher magnification of the areas delineated by the dashed boxes. White arrows point to examples of microglia. (**B**) Fraction of Iba1 immunopositive area in the striatum and motor cortex of 8 and 13-week-old WT and R6/2 mice. Two-way ANOVA with Bonferroni’s multiple comparison test, not significant. *n* = 4 (8 weeks) or 3–4 mice (13 weeks) per group. (**C**) Representative immunoblot for GFAP in striatal lysates of 13-week-old WT and R6/2 mice treated with placebo or anle138b. GAPDH was used as a loading control. (**D**) Quantification of GFAP expression levels in the striatum and motor cortex of 8- and 13-week-old WT and R6/2 mice. Values were normalized to WT/placebo. Two-way ANOVA with Bonferroni’s multiple comparison test, per age group and brain region. Striatum, 8 weeks: not significant; 13 weeks: Treatment, *p = 0.0391; Genotype, **p = 0.0064; Treatment × Genotype, *p = 0.0291. Motor cortex, 8 weeks: not significant; 13 weeks: Treatment, *p = 0.0478; Genotype, *p = 0.0140; Treatment × Genotype, *p* = 0.0687. *n* = 3 mice per group. (**E**) Representative immunoblot for Iba1 in striatal lysates of 13-week-old WT and R6/2 mice treated with placebo or anle138b. GAPDH was used as a loading control. (**F**) Quantification of Iba1 expression levels in the striatum and motor cortex of 8- and 13-week-old WT and R6/2 mice. Values were normalized to WT/placebo. Two-way ANOVA with Bonferroni’s multiple comparison test, per age group and brain region, not significant. *n* = 3 mice per group. Data presented as mean ± SD. *p* values for significant pairwise comparisons are indicated on the graphs. Scale bars in (**B**) 100 µm; insets, 50 µm. Source data are available online for this figure

A prominent feature of HD is neuroinflammation, characterized by altered morphology and density of astrocytes and microglial cells (Palpagama et al, 2019; Wilton and Stevens, 2020). To assess the impact of anle138b on astrogliosis and microgliosis, we performed immunostainings for the astrocyte marker glial fibrillary acidic protein (GFAP) and microglial marker Iba1, and quantified the immunoreactive area in the striatum and motor cortex. GFAP immunoreactive area was significantly increased in both brain regions of placebo-treated R6/2 mice at 13 weeks of age. This increase was diminished by anle138b treatment (Fig. 3E,F). We also observed a trend towards increased Iba1 immunoreactive area in placebo-treated R6/2 mice, but this effect did not reach statistical significance, precluding us from assessing the impact of anle138b on microglia (Fig. EV2A,B). In addition, we assessed the protein levels of GFAP and Iba1 by Western blot. In agreement with the immunostaining results, GFAP levels were increased in the striatum and motor cortex of placebo-treated, but not anle138b-treated R6/2 mice (Fig. EV2C,D). Iba1 levels in placebo-treated mice showed a trend towards an increase, which did not reach statistical significance. In anle138b-treated mice, Iba1 levels were similar to controls (Fig. EV2E,F). In summary, these data illustrate the beneficial effects of anle138b on brain size and neuroinflammation markers in the R6/2 mouse brain.

### Anle138b reduces mHTT inclusion load and reverses neurochemical changes in the R6/2 brain

**Figure 4:**
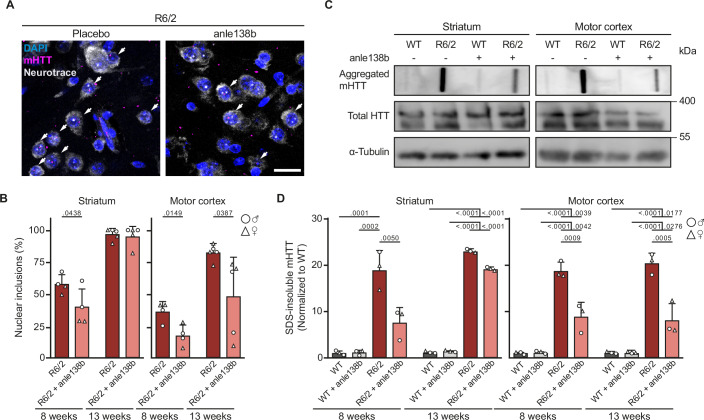
Anle138b reduces mHTT aggregate load in R6/2 mice. (**A**) Representative images of the motor cortex of 13-week-old R6/2 mice treated with placebo or anle138b. Neurons were identified by Neurotrace labeling, and aggregated mHTT was detected by EM48 immunostaining. Nuclei were labeled with DAPI. White arrows point to neurons with mHTT inclusion bodies. (**B**) Quantification of the fraction of neurons with nuclear mHTT inclusion bodies in the striatum and motor cortex of R6/2 mice. Unpaired two-tailed *t*-test. *n* = 4 (8 weeks) or 5 mice (13 weeks). (**C**) Filter trap membranes of lysates from the striatum and motor cortex of 8-week-old WT and R6/2 mice treated with placebo or anle138b. Total levels of HTT were determined by immunoblotting, and α-Tubulin was used as a loading control. (**D**) Quantification of the filter trap assay. Values were normalized to WT/placebo. Two-way ANOVA with Bonferroni’s multiple comparison test. Striatum at 8 weeks: Treatment, **p = 0.0064; Genotype, ****p < 0.0001; Treatment × Genotype, **p = 0.0061. Striatum at 13 weeks: Treatment, ****p < 0.0001; Genotype, ****p < 0.0001; Treatment × Genotype, ****p < 0.0001. Motor cortex at 8 weeks: Treatment, **p = 0.0016; Genotype, ****p < 0.0001; Treatment × Genotype, **p = 0.0014. Motor cortex at 13 weeks: Treatment, **p = 0.0012; Genotype, ****p < 0.0001; Treatment × Genotype, ***p = 0.0007. *n* = 3 mice per group. Data presented as mean ± SD. *p* values for significant pairwise comparisons are indicated on the graphs. Scale bar in (**A**), 20 µm. Source data are available online for this figure.

As anle138b reduced the frequency of mHTT inclusion bodies in cultured neurons, we next assessed its impact on mHTT inclusion load in the mouse brain. EM48 staining for aggregated HTT revealed abundant neuronal inclusions in the cortex and striatum of R6/2 mice (Fig. 4A), as described previously (Davies et al, 1997; Hosp et al, 2017). As nuclear and cytoplasmic mHTT inclusion bodies have distinct properties and toxicity mechanisms (Blumenstock et al, 2021; Landles et al, 2020; Riguet et al, 2021), we analyzed these two types of inclusions separately. For nuclear inclusions, we observed a significant reduction in the fraction of inclusion-positive neurons, with the exception of 13-week-old striatal samples, an advanced time point when the immunohistochemically detectable inclusion load in the striatum is saturated (Fig. 4B). Cytoplasmic inclusions were rare at 8 weeks of age in both brain regions, but at 13 weeks they became more numerous, and we detected a beneficial effect of anle138b on the fraction of inclusion-bearing neurons in the motor cortex (Appendix Fig. S3B). The treatment did not have a pronounced effect on the inclusion size (Appendix Fig. S3C). In line with the histological analyses, the filter trap assay revealed a decrease in aggregated mHTT in anle138b-treated brains from 8 weeks on (Fig. 4C,D).

**Figure 5:**
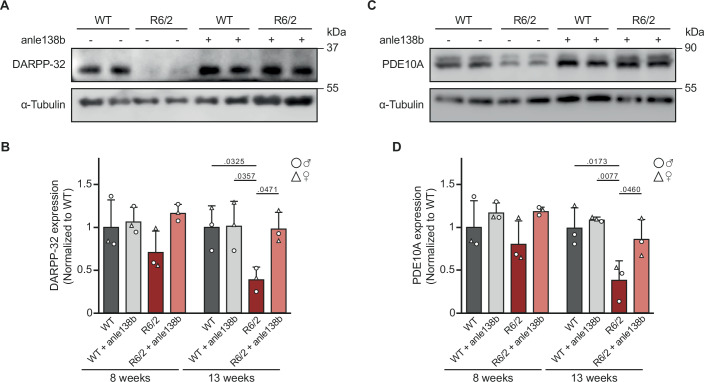
Anle138b treatment rescues expression of striatal markers in R6/2 mice. (**A**) Representative immunoblot for DARPP-32 in striatal lysates of 13-week-old WT and R6/2 mice treated with placebo or anle138b. (**B**) Quantification of DARPP-32 expression levels in the striatum of 8 and 13-week-old WT and R6/2 mice. Values were normalized to WT/placebo. Two-way ANOVA with Bonferroni’s multiple comparison test. 8 weeks: not significant; 13 weeks: Treatment, *p = 0.0386; Genotype, *p = 0.0222; Treatment × Genotype, *p = 0.0334. (**C**) Representative immunoblot for PDE10A in striatal lysates of 13-week-old WT and R6/2 mice treated with placebo or anle138b. (**D**) Quantification of PDE10A expression levels in the striatum of 8 and 13-week-old WT and R6/2 mice. Values were normalized to WT/placebo. Two-way ANOVA with Bonferroni’s multiple comparison test. 8 weeks: not significant; 13 weeks: Treatment, *p = 0.0215; Genotype, **p = 0.0035; Treatment × Genotype, *p* = 0.0711. In (**A**, **C**), α-Tubulin was used as loading control. For all assays, *n* = 3 mice per group. Data presented as mean ± SD. *p* values for significant pairwise comparisons are indicated on the graphs. Source data are available online for this figure.

Neurodegenerative changes in the striatum of HD mice include the loss of medium spiny neuron markers such as dopamine- and cAMP-regulated neuronal phosphoprotein of 32 kDa (DARPP-32) and phosphodiesterase 10 A (PDE10A) (Bibb et al, 2000; Menalled et al, 2012; Suelves et al, 2019). PDE10A downregulation also occurs in HD patients, and PDE10A tracers are used in human positron emission tomography (PET) imaging as an early-stage HD biomarker (Niccolini et al, 2015; Russell et al, 2014). We therefore quantified these marker proteins in the striatum of anle138b-treated mice by western blot. R6/2 mice displayed reduced levels of both DARPP-32 and PDE10A in striatal lysates at 13 weeks of age. Remarkably, anle138b administration reversed the loss of both marker proteins (Fig. 5A–D). These results indicate that anle138b treatment decreases mHTT aggregate load and prevents loss of striatal neuron markers in R6/2 mice.

### Anle138b mitigates synapse loss in the striatum of R6/2 mice

**Figure 6:**
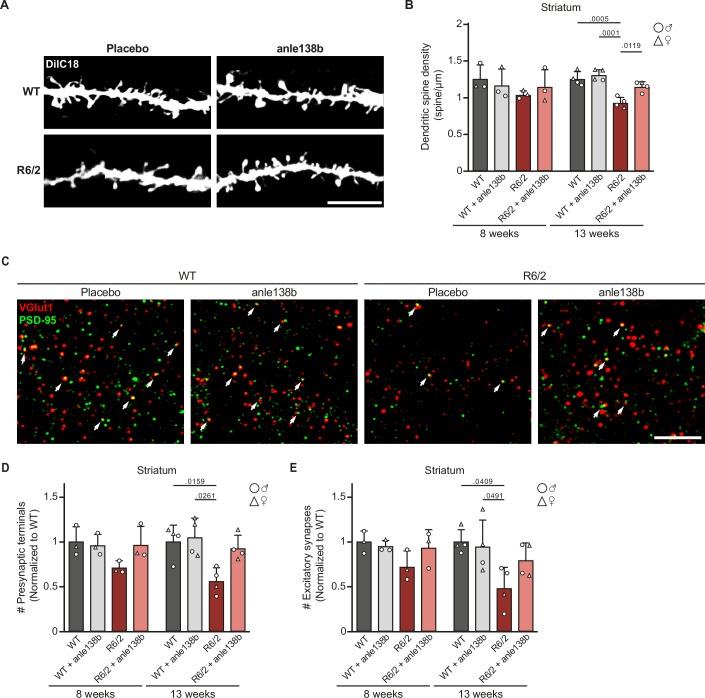
Anle138b mitigates synapse loss in the striatum of R6/2 mice. (**A**) Representative images of DiIC18 labeling showing dendrites with spines in the striatum of 13-week-old WT and R6/2 mice treated with placebo or anle138b. (**B**) Quantification of dendritic spine density in the striatum of 8- and 13-week-old WT and R6/2 mice. Two-way ANOVA with Bonferroni’s multiple comparison test, per age group. 8 weeks: not significant; 13 weeks: Treatment, **p < 0.0053; Genotype, ****p < 0.0001; Treatment × Genotype, *p* = 0.0517. *n* = 3 mice (8 weeks) or 4 mice (13 weeks) per genotype and treatment group. (**C**) Representative images of immunostained VGlut1 and PSD-95 puncta in the striatum of 13-week-old WT and R6/2 mice treated with placebo or anle138b. Excitatory synapses were identified by the overlap between the puncta in the two channels (examples indicated by white arrows). (**D**) Quantification of the number of VGlut1 puncta in the striatum of 8 and 13-week-old WT and R6/2 mice. Two-way ANOVA with Bonferroni’s multiple comparison test, per age group. 8 weeks: not significant; 13 weeks: Treatment, *p* = 0.07; Genotype, **p < 0.0059; Treatment × Genotype, *p* = 0.0688. *n* = 3 mice (8 weeks) or 4 mice (13 weeks) per genotype and treatment group. (**E**) Quantification of the number of overlapping VGlut1/PSD-95 puncta in the striatum of 8- and 13-week-old WT and R6/2 mice. Two-way ANOVA with Bonferroni’s multiple comparison test, per age group. 8 weeks: not significant; 13 weeks: Treatment, *p* = 0.2670; Genotype, *p < 0.0122; Treatment × Genotype, *p* = 0.1217. *n* = 3 mice (8 weeks) or 4 mice (13 weeks) per genotype and treatment group. Data presented as mean ± SD. *p* values for significant pairwise comparisons are indicated on the graphs. Scale bars in (**A**, **C**), 5 µm. Source data are available online for this figure.

Synaptic defects constitute an important hallmark of neurodegenerative diseases including HD, where loss of glutamatergic corticostriatal inputs precedes and likely triggers dysfunction and degeneration of medium spiny neurons (Deng et al, 2013; Estrada-Sanchez and Rebec, 2013; Langfelder et al, 2016; Spampanato et al, 2008; Uytterhoeven et al, 2025). Previous studies demonstrated reduction in dendritic spines and downregulation of synaptic marker proteins in HD mouse models (Hosp et al, 2017; Indersmitten et al, 2015; Langfelder et al, 2016; Morton and Edwardson, 2001; Murmu et al, 2013). We therefore investigated the ability of anle138b to ameliorate these impairments in the striatum of R6/2 mice. First, we visualized the dendritic arbors of the striatal medium spiny neurons in brain sections by DiIC18 labeling and counted spines on the proximal dendrites. Dendritic spine density was significantly reduced in placebo-treated R6/2 mice at 13 weeks of age. This reduction was rescued by anle138b treatment (Fig. 6A,B).

Dendritic spines are a major site of excitatory synaptic inputs. We therefore assessed the density of excitatory synapses by co-immunostaining for the excitatory presynaptic marker VGlut1 and postsynaptic marker PSD-95. We observed a lower density of VGlut1-positive presynaptic terminals in placebo-treated 13-week-old R6/2 mice (Fig. 6C,D). Accordingly, the density of synapses assessed by the number of overlapping VGlut1 and PSD-95 puncta was also significantly reduced (Fig. 6E). In contrast, the density of presynaptic terminals and overlapping VGlut1 and PSD-95 puncta in anle138b-treated R6/2 mice was not significantly different from wild-type littermates (Fig. 6C–E). These findings suggest that anle138b partially prevents synapse loss in striatal medium spiny neurons of R6/2 mice.

### Anle138b ameliorates disease phenotypes in zQ175DN knock-in HD mice

**Figure EV3:**
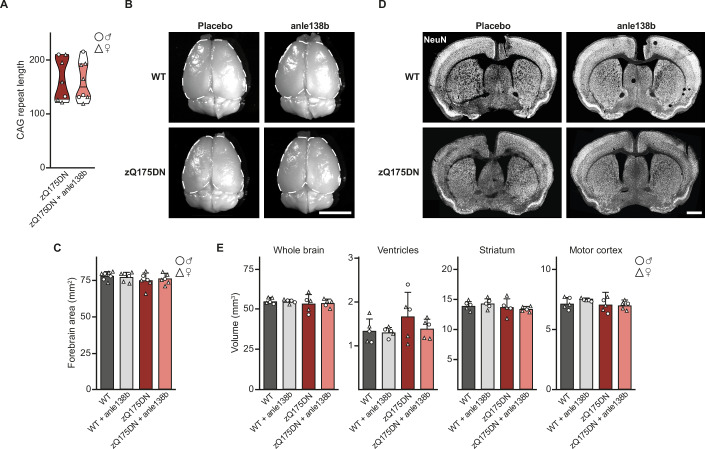
Unchanged brain morphology in zQ175DN mice. (**A**) CAG repeat length in 9-month-old zQ175DN mice. Unpaired two-tailed *t*-test, not significant. *n* = 7–8 mice per group. (**B**) Representative images of the brains of 9-month-old WT and zQ175DN mice treated with placebo or anle138b. Dashed white lines outline the forebrain. (**C**) Quantification of forebrain area in 9-month-old WT and zQ175DN mice. Two-way ANOVA with Bonferroni’s multiple comparison test, not significant. *n* = 6–7 mice per group. (**D**) Representative coronal brain sections of 9-month-old WT and zQ175DN mice treated with placebo or anle138b, immunostained for the neuronal marker NeuN. (**E**) Whole brain, ventricle, striatum and motor cortex volume quantification in 9-month-old WT and zQ175DN mice. Two-way ANOVA with Bonferroni’s multiple comparison test, not significant. *n* = 5 mice per group. Data presented as mean ± SD. Scale bars in (**B**) 5 mm; (**D**) 1 mm. Source data are available online for this figure

**Figure 7:**
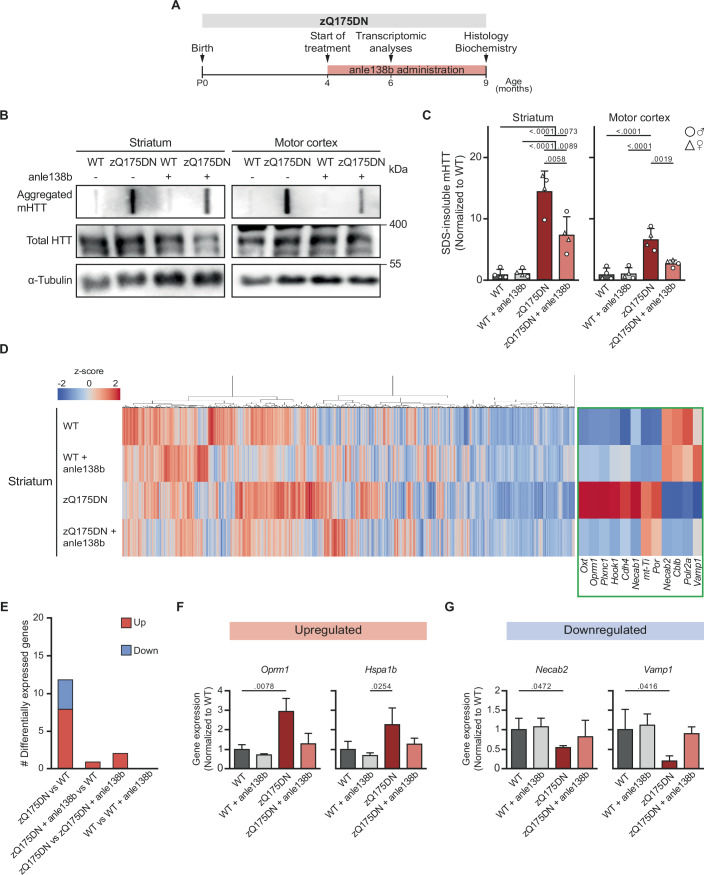
Anle138b mitigates HD phenotypes in zQ175DN mice. (**A**) Experimental timeline for the zQ175DN mice. (**B**) Filter trap of lysates from the striatum and motor cortex of 9-month-old WT and zQ175DN mice treated with placebo or anle138b. Total levels of HTT were determined by immunoblotting, α-Tubulin was used as a loading control. (**C**) Quantification of aggregated mHTT in the striatum and motor cortex of 9-month-old WT and zQ175DN mice. Values were normalized to WT/placebo. Two-way ANOVA with Bonferroni’s multiple comparison test. Striatum: Treatment, *p* = 0.2715; Genotype, **p = 0.0017; Treatment × Genotype, *p* = 0.2208. Motor cortex: Treatment, **p = 0.01; Genotype, ***p = 0.0001; Treatment × Genotype, **p = 0.0074. *n* = 3–4 mice per group. (**D**) Heatmap showing differentially expressed genes in the striatum of 6-month-old WT and zQ175DN mice treated with placebo or anle138b. The heatmap cluster on the right shows the 12 differentially expressed genes in placebo-treated zQ175DN mice compared to WT littermates. (**E**) Number of up- and down-regulated genes between the groups. (**F**) Expression of the upregulated genes *Oprm1* and *Hspa1b* in the striatum of 6-month-old WT and zQ175DN mice treated with placebo or anle138b. Two-way ANOVA with Bonferroni’s multiple comparison test. *Oprm1*: Treatment, **p = 0.0073; Genotype, *p = 0.0114; Treatment × Genotype, *p = 0.0255; *Hspa1*: Treatment, *p = 0.0234; Genotype, *p = 0.0234; Treatment × Genotype, *p* = 0.1238. (**G**) Expression of the downregulated genes *Necab2* and *Vamp1* in the striatum of 6-month-old WT and zQ175DN mice treated with placebo or anle138b. Two-way ANOVA with Bonferroni’s multiple comparison test. *Necab2*: Treatment, *p = 0.0224; Genotype, *p* = 0,1135; Treatment × Genotype, *p* = 0.0635; *Vamp1*: Treatment, *p = 0.0462; Genotype, *p = 0.0254; Treatment × Genotype, *p* = 0.1560. For all transcriptomic analyses shown in (**D**–**G**), *n* = 4 WT, 3 WT + anle138b, 7 zQ175DN and 4 zQ175DN + anle138b mice. Data presented as mean ± SD. *p* values for significant pairwise comparisons are indicated on the graphs. Source data are available online for this figure.

Having observed a clear disease-modifying effect of anle138b in R6/2 mice, we sought to validate these findings in another HD model more similar to adult-onset HD. To this end, we used heterozygous zQ175DN knock-in mice expressing full-length *Htt* with ~170 CAG repeats from the endogenous murine *Htt* locus (Fig. EV3A) (Menalled et al, 2012; Southwell et al, 2016). As treatments during the entire pre- and postnatal development are difficult in human patients, we furthermore tested a different anle138b administration regime. The compound was delivered at the same concentration of 2 g/kg as in the R6/2 model, but the treatment was started in adult mice at 4 months of age, and histological and biochemical analyses were conducted in 9-month-old animals (Fig. 7A). We did not observe clear behavioral defects in heterozygous zQ175DN mice in a previous study (Voelkl et al, 2023), therefore here we focused on the assessment of brain morphology, mHTT aggregation and expression of striatal markers.

**Figure EV4:**
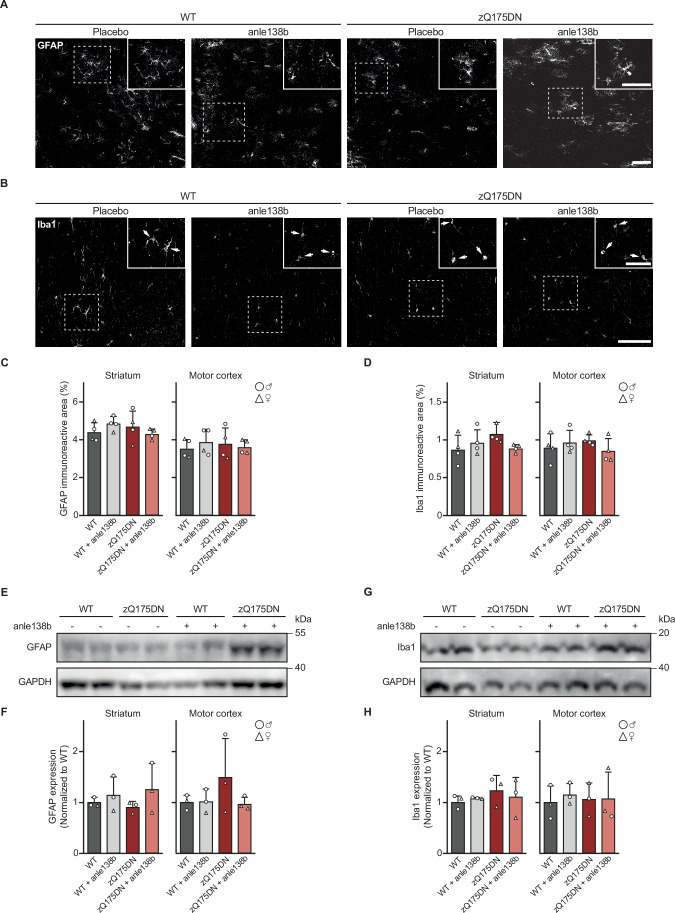
zQ175DN mice do not exhibit signs of neuroinflammation. (**A**) Representative images of GFAP immunostaining in the dorsal striatum of 9-month-old WT and zQ175DN mice treated with placebo or anle138b. Insets show magnification of the areas delineated by the white dashed boxes. (**B**) Representative images of Iba1 immunostaining in the dorsal striatum of 9-month-old WT and zQ175DN mice treated with placebo or anle138b. Insets show magnification of the areas delineated by the white dashed boxes. Arrows point to examples of microglia. (**C**) Fraction of GFAP immunopositive area in the striatum and motor cortex of 9-month-old WT and zQ175DN mice. Two-way ANOVA with Bonferroni’s multiple comparison test, not significant. *n* = 4 mice per group. (**D**) Fraction of Iba1 immunopositive area in the striatum and motor cortex of 9-month-old WT and zQ175DN mice. Two-way ANOVA with Bonferroni’s multiple comparison test, not significant. *n* = 4 mice per group. (**E**) Representative immunoblot for GFAP in striatal lysates of 9-month-old WT and zQ175DN mice treated with placebo or anle138b. GAPDH was used as a loading control. (**F**) Quantification of GFAP expression levels in the striatum and motor cortex of 9-month-old WT and zQ175DN mice. Values were normalized to WT/placebo. Two-way ANOVA with Bonferroni’s multiple comparison test, not significant. *n* = 3 mice per group. (**G**) Representative immunoblot for Iba1 in striatal lysates of 9-month-old WT and zQ175DN mice treated with placebo or anle138b. GAPDH was used as a loading control. (**H**) Quantification of Iba1 expression levels in the striatum and motor cortex of 9-month-old WT and zQ175DN mice. Values were normalized to WT/placebo. Two-way ANOVA with Bonferroni’s multiple comparison test, not significant. *n* = 3 mice per group. Data presented as mean ± SD. Scale bars in (**A**, **B**) 100 µm; insets, 50 µm. Source data are available online for this figure

**Figure EV5:**
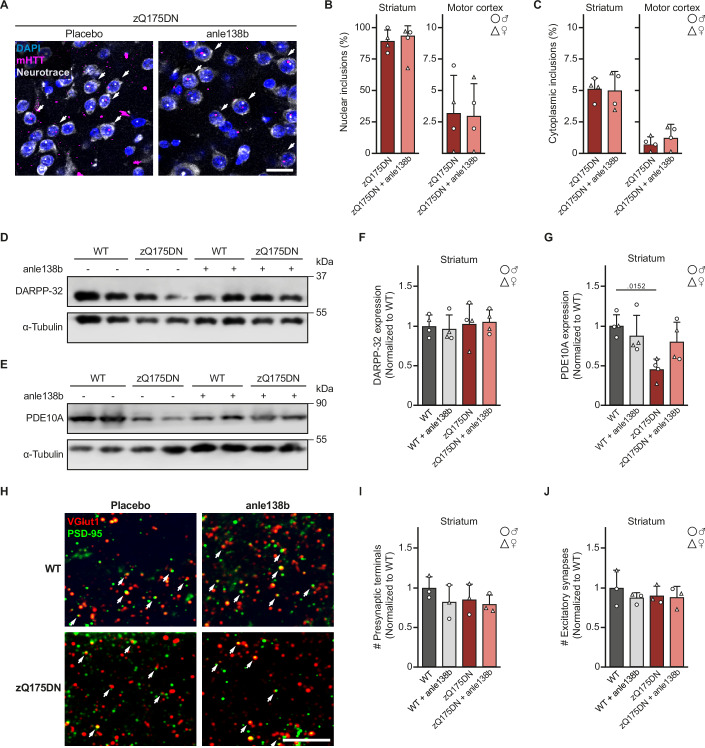
Effects of anle138b on inclusion load, striatal markers and synapse density in zQ175DN mice. (**A**) Representative images of neurons with nuclear mHTT inclusions (magenta) in the motor cortex of 9-month-old zQ175DN mice treated with placebo or anle138b. Neurons were identified by Neurotrace labeling, and aggregated mHTT was detected by EM48 immunostaining. Nuclei were labeled with DAPI. White arrows point to neurons with mHTT inclusion bodies. (**B**) Quantification of the fraction of neurons with nuclear mHTT inclusion bodies in the striatum and motor cortex of 9-month-old zQ175DN mice. Unpaired two-tailed *t*-test, not significant. *n* = 4 mice per group. (**C**) Quantification of the fraction of neurons with cytoplasmic mHTT inclusion bodies in the striatum and motor cortex of 9-month-old zQ175DN mice. Unpaired two-tailed *t*-test, not significant. *n* = 4 mice per group. (**D**) Representative immunoblot for DARPP-32 in striatal lysates of 9-month-old WT and zQ175DN mice treated with placebo or anle138b. α-Tubulin was used as a loading control. (**E**) Representative immunoblot for PDE10A in striatal lysates of 9-month-old WT and zQ175DN mice treated with placebo or anle138b. α-Tubulin was used as a loading control. (**F**) Quantification of DARPP-32 expression levels. Values were normalized to WT/placebo. Two-way ANOVA with Bonferroni’s multiple comparison test, not significant. *n* = 4 mice per group. (**G**) Quantification of PDE10A expression levels. Values were normalized to WT/placebo. Two-way ANOVA with Bonferroni’s multiple comparison test. Treatment, *p* = 0.3005; Genotype, *p = 0.01; Treatment × Genotype, *p = 0.0390. *n* = 4 mice per group. (**H**) Representative images of immunostained VGlut1 and PSD-95 puncta in the dorsal striatum of 9-month-old WT and zQ175DN mice treated with placebo or anle138b. Excitatory synapses were identified by the overlap between the puncta in the two channels (examples indicated by white arrows). (**I**) Quantification of the number of VGlut1 puncta in the striatum of 9-month-old WT and zQ175DN mice. Two-way ANOVA with Bonferroni’s multiple comparison test, not significant. *n* = 3 mice per group. (**J**) Quantification of the number of overlapping VGlut1/PSD-95 puncta in the striatum of 9-month-old WT and zQ175DN mice. Two-way ANOVA with Bonferroni’s multiple comparison test, not significant. *n* = 3 mice per group. Data presented as mean ± SD. *p* values for significant pairwise comparisons are indicated on the graphs. Scale bars: (**A**) 20 µm; (**H**) 5 µm. Source data are available online for this figure

We did not detect significant differences in the area of the whole forebrain of zQ175DN mice compared to wild-type littermate controls, nor in the volume of the whole brain, the lateral ventricles, striatum or motor cortex (Fig. EV3B–E). Immunostaining and western blot for GFAP and Iba1 also did not reveal signs of astro- or microgliosis at 9 months of age (Fig. EV4A–H). We therefore could not assess the potential beneficial effects of anle138b on these parameters. The fraction of neurons with nuclear and cytoplasmic mHTT inclusion bodies in the striatum and motor cortex was not different between placebo- and anle138b-treated zQ175DN animals (Fig. EV5A–C). However, filter trap assay showed significant accumulation of aggregated mHTT species in the striatum and motor cortex of placebo-treated zQ175DN mice, and this accumulation was reduced by anle138b treatment in the motor cortex (Fig. 7B,C). We next quantified the protein levels of striatal markers by Western blot. While the levels of DARPP-32 were not significantly changed in zQ175DN mice at 9 months of age (Fig. EV5D,F), PDE10A levels were decreased in placebo-treated, but not in anle138b-treated mice (Fig. EV5E,G). The density of excitatory pre- and postsynaptic markers was not changed in the striatum of zQ175DN mice, precluding assessment of anle138b’s effect on synapse loss in this model (Fig. EV5H–J). Taken together, these findings demonstrate that anle138b reduces mHTT aggregation and prevents reduction in PDE10A levels in the zQ175DN full-length knock-in model of HD.

### Anle138b mitigates transcriptional dysregulation in zQ175DN mice

To explore the molecular pathways rescued by anle138b in HD mice, we treated zQ175DN mice with the compound starting from 4 months and harvested striatal and cortical tissue for RNA-seq at 6 months, an early time point in disease progression when transcriptional dysregulation was reported in this model (Langfelder et al, 2016). Transcriptional changes detected in zQ175DN mice were overall mild, consistent with the presymptomatic stage of the mice. In the striatum of placebo-treated zQ175DN mice, we detected 12 differentially expressed genes (DEGs), of which eight were upregulated and four were downregulated compared to wild-type littermates. In contrast, comparison of anle138b-treated zQ175DN mice and wild-type controls revealed only one upregulated gene (Fig. 7D,E). Among the genes upregulated in placebo-treated zQ175DN animals was the μ-type opioid receptor (*Oprm1*), previously shown to be increased in HD mice (Morigaki et al, 2020), and the chaperone heat shock 70 kDa protein 1B (*Hspa1b*) (Fig. 7F). Among the downregulated DEGs, we found genes encoding the calcium-binding protein NECAB2 and the synaptic SNARE protein VAMP1 (Fig. 7G). Of note, the levels of *Htt* transcript were not changed between the experimental groups, indicating that anle138b does not modify HTT expression (Appendix Fig. S4D). Consistent with the unchanged levels of the glial proteins GFAP and Iba1 (Fig. EV4A–H), striatal markers DARPP-32 and PDE10A (Fig. EV5D–G), and synaptic proteins VGlut1 and PSD-95 (Fig. EV5H–J) in zQ175DN mice at 9 months of age, we did not observe dysregulation of the respective RNAs (Appendix Fig. S4A–C). RNA-seq of the cortical tissue did not reveal significant DEGs between any of the conditions (Appendix Fig. S4E). This is consistent with the mild mHTT pathology detected in the cortex of the zQ175DN mouse model (Fig. EV5A–C). These findings suggest that anle138b mitigates transcriptional dysregulation in the HD mouse brain.

### Anle138b reduces mHTT aggregates in human HD-iPSC-derived neural precursor cells

**Figure 8:**
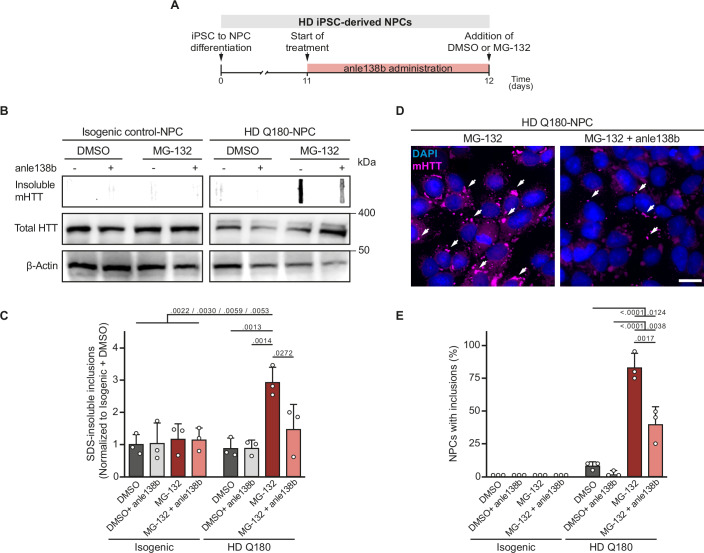
Anle138b reduces mHTT aggregation in human HD-iPSC-derived NPCs. (**A**) Experimental timeline for HD-iPSC-derived NPC cultures. (**B**) Filter trap of HD Q180-NPCs and isogenic control-NPCs treated with vehicle (DMSO) or 7 µM anle138b. Total levels of HTT were determined by immunoblotting; β-Actin served as a loading control. (**C**) Quantification of insoluble mHTT. Values were normalized to vehicle-treated isogenic control-NPCs. Three-way ANOVA with Bonferroni’s multiple comparison test. mHTT, *p = 0.0296; MG-132, **p = 0.0015; anle138b, *p* = 0.0875; mHTT × MG-132, **p = 0.0080; mHTT × anle138b, *p* = 0.0718; MG-132 × anle138b, *p* = 0.0608; mHTT × MG-132 × anle138b, *p* = 0.0906. *n* = 3 independent experiments. (**D**) Representative images of HD Q180-NPCs treated with 5 µM MG-132 and vehicle (DMSO, left) or 7 µM anle138b (right). Aggregated mHTT was detected by anti-PolyQ immunostaining and nuclei were labeled with Hoechst 33342. White arrows highlight mHTT inclusion bodies. (**E**) Quantification of the fraction of NPCs with mHTT inclusion bodies. Two-way ANOVA with Bonferroni’s multiple comparison test: MG-132, ****p < 0.0001; anle138b, **p = 0.0011; MG-132 × anle138b **p = 0.0063. *n* = 3 independent experiments. Data were presented as mean ± SD. *p* values for significant pairwise comparisons are indicated on the graphs. Scale bar in (**D**) 20 µm. Source data are available online for this figure.

Having observed positive effects of anle138b in murine neurons and HD mouse models, we aimed to validate these findings in a human model system derived from HD patients. To this end, we used two independent HD-iPSCs lines (Q71 and Q180). For the Q180 line, an isogenic control was available. We differentiated the iPSCs into neural progenitor cells (NPCs) and treated the cultures for 24 h with anle138b or vehicle control. Because of the lack of mHTT-dependent cell death in NPCs (Koyuncu et al, 2018), we focused our analyses on mHTT aggregation in this system. As NPCs do not develop mHTT inclusions spontaneously, we used the proteasome inhibitor MG-132 to induce mHTT aggregation (Fig. 8A). Filter trap assay revealed a marked increase in aggregated mHTT upon addition of MG-132, which was prevented by anle138b in both lines (Fig. 8B,C; Appendix Fig. S5A,B). In addition, we analyzed mHTT inclusion formation in the Q180 line by immunofluorescence. MG-132 treatment resulted in the formation of mHTT inclusion bodies in the majority of Q180-NPCs, while the fraction of cells with inclusions was significantly diminished by anle138b treatment (Fig. 8D,E). These results demonstrate the efficacy of anle138b in reducing mHTT aggregation in a human model system with endogenous expression of mHTT.

## Discussion

Here, we show that the small molecule anle138b significantly reduces mHTT toxicity and ameliorates disease phenotypes in a primary neuron model and two different mouse models of HD. The treatment had beneficial effects not only when administered throughout embryonic development and postnatal life, but also when started at an adult age. The zQ175DN knock-in model only displayed a few significant phenotypes (mHTT aggregation, decreased PDE10A protein levels). The effect of anle138b on those phenotypes was in agreement with the findings in the R6/2 model (Appendix Table S1). Moreover, we demonstrate that anle138b ameliorated transcriptional alterations in this line already at the preclinical age of 6 months after only 2 months of treatment. It should be mentioned that the transcriptional changes detected in our study in 6-month-old zQ175DN mice were very mild compared to previous findings (Langfelder et al, 2016; Lee et al, 2020). This might be due to the low sample size, colony differences and/or methodological aspects, such as the use of 3’ RNA-seq technique that has limited sensitivity compared to full-length bulk RNA-seq. Nevertheless, the subtle transcriptional dysregulation that we were able to detect was partially rescued by anle138b treatment. In the R6/2 line, we additionally show that anle138b improved motor defects seen at the advanced disease stage, prolonged life span, reduced brain atrophy, astrogliosis and synapse loss (Appendix Table S1). The mild nature of the zQ175DN knock-in model precluded the assessment of anle138b effects on these phenotypes in this mouse line (Voelkl et al, 2023). Importantly, anle138b also inhibited mHTT aggregation in human NPCs differentiated from HD-iPSCs, confirming its efficiency in human neuronal cells with endogenous full-length mHTT. Of note, anle138b did not alter HTT expression and did not cause adverse effects in wild-type mice in any of the described assays. These findings indicate that anle138b may be a possible therapeutic option for HD.

A number of other small molecules have shown disease-modifying potential in HD mice (Di Pardo et al, 2014; Ferrante et al, 2004; Hsiao et al, 2014; Masuda et al, 2008; Peng et al, 2008; Reick et al, 2016; Reiner et al, 2012; Squitieri et al, 2015). One of these compounds, the sigma-1 receptor agonist Pridopidine (Squitieri et al, 2015), is currently in clinical testing. Another compound, the vesicle monoamine transporter inhibitor Deutetrabenazine, is used for treating HD-associated chorea (Anderson et al, 2017). The splicing modulator Branaplam showed promising results in mouse and iPSC models of HD (Keller et al, 2022; Krach et al, 2022), but turned out to have neurotoxic side effects in a phase 2 clinical trial (NCT05111249), illustrating the challenges of developing small-molecule therapeutics for HD. Of note, the mechanisms of action of these drugs are distinct from anle138b, which reduces mHTT aggregate load without detectable impact on other cellular pathways. While the effects of anle138b in HD mice are similar in magnitude to other small molecules, many other treatments had to be administered through subcutaneous, intraperitoneal or intraventricular injections. In contrast, anle138b has excellent oral bioavailability. Moreover, the available results from clinical phase 1 trials indicate a favorable safety and tolerability profile in humans (Levin et al, 2022).

In both HD models used in the study, we administered anle138b to presymptomatic animals before the onset of prominent degenerative changes in the brain and manifestation of neurological symptoms. As HD is a hereditary disorder and mutation carriers can be identified in the presymptomatic phase, preventive approaches for this disease are feasible. In future studies, it will be interesting to determine whether anle138b treatment is also beneficial when started after symptom onset.

It should be noted that while behavioral and neuropathological phenotypes were clearly improved by anle138b treatment in animals of either sex, a positive effect on life span was detected only in female mice. Sex-specific differences in clinical manifestations and treatment efficacy are documented in human patients and animal models of several neurodegenerative diseases, including HD. In particular, female HD patients show, on average, more severe symptomology with regard to both motor and cognitive deficits (Hentosh et al, 2021). Furthermore, several experimental treatments for HD produced effects of different magnitude depending on the sex of the animals (Ma et al, 2007; Reiner et al, 2012), in line with our observations for anle138b. The causes of these differences remain poorly understood and represent an important subject for future research.

What could be the potential cellular substrate for anle138b-mediated improvements in neurological symptoms? We show that the treatment partially prevented loss of spines and excitatory synapses on striatal medium spiny neurons. A major source of excitatory inputs to the striatum is the neocortex, and dysfunction and degeneration of the corticostriatal afferents is believed to be a likely trigger of medium spiny neuron demise (Deng et al, 2013; Estrada-Sanchez and Rebec, 2013). Improved synaptic integrity and corticostriatal communication is therefore a likely contributing factor in the beneficial effects of anle138b, although other brain regions and cellular substrates might also play a role.

Further experiments are needed to clarify the mechanistic details of anle138b action at the molecular level. The rescue of HD-related phenotypes in all utilized models correlated with the ability of anle138b to reduce mHTT aggregate load, suggesting that the functional improvements likely result from a reduction in aggregated species of mHTT. Experiments with other purified recombinant aggregating proteins such as tau and α-synuclein showed that anle138b can directly modulate aggregation by binding to oligomers (Albariqi et al, 2024; Heras-Garvin et al, 2019; Martinez Hernandez et al, 2018; Wagner et al, 2015; Wagner et al, 2013; Wegrzynowicz et al, 2019). It should be kept in mind that in living cells, disease-related protein aggregates, including mHTT aggregates, show a great heterogeneity of conformations and biological effects (Gracia et al, 2020; Isas et al, 2021; Riguet et al, 2021; Swanson et al, 2025). Actions of anle138b may therefore be distinct depending on the mHTT inclusion type. In the human brain, two major subcellular locations of mHTT inclusion bodies are the nucleus and the cytoplasm of neurites (DiFiglia et al, 1997). Anle138b reduced the frequency of both nuclear and cytoplasmic inclusions in primary neurons and in the HD mouse brain. Interestingly, nuclear and cytoplasmic mHTT inclusions appear to have different protein composition, biochemical and ultrastructural properties, and distinct mechanisms of toxicity (Landles et al, 2020; Riguet et al, 2021). In particular, our previous findings indicate that cytoplasmic mHTT inclusions markedly impair cellular proteostasis (Blumenstock et al, 2021). Using the Fluc-EGFP proteostasis reporter, we show that anle138b ameliorates this impairment. Nuclear inclusions might be less burdensome for the proteostasis system, and the details of anle138b effects in this compartment remain to be explored. In addition, a crucial pathogenic mechanism of HD that has recently come into focus is somatic instability of CAG repeats (Handsaker et al, 2025; Tabrizi et al, 2020). It will therefore be important in future studies to also investigate the ability of anle138b to modulate somatic repeat expansion. In summary, our results provide evidence of the promise of anle138b as a disease-modifying therapy for HD. As CAG trinucleotide repeat disorders share several common mechanisms (Everett and Wood, 2004), anle138b might also be beneficial in CAG expansion diseases beyond HD.

## Methods

**Table:** Reagents and tools table

Reagent/resource	Reference or source	Identifier or catalog number
**Experimental models**		
R6/2 (*M. musculus*)	The Jackson Laboratory	Stock #002810
zQ175DN (*M. musculus*)	The Jackson Laboratory	Stock #029928
HD Q71-iPSC	Kindly provided by G. Q. Dailey (Park et al, 2008)	
HD Q180-iPSC	NINDS iPSC Repository	ID: ND36999
HD Q180-iPSC isogenic control	Kindly provided by M. A. Pouladi (Xu et al, 2017)	
**Recombinant DNA**		
pcDNA3.1 mCherry	Kindly provided by F. U. Hartl (Hipp et al, 2012)	
pcDNA3.1 HTT-Exon1-Q25-mCherry-myc-His	Kindly provided by F. U. Hartl (Hipp et al, 2012)	
pcDNA3.1 HTT-Exon1-Q97-mCherry-myc-His	Kindly provided by F. U. Hartl (Hipp et al, 2012)	
pcDNA3.1 HTT-Exon1-Q25-His	Cloned in-house (Voelkl et al, 2023)	
pcDNA3.1 HTT-Exon1-Q72-His	Cloned in-house (Voelkl et al, 2023)	
NES-Fluc-EGFP	Kindly provided by F. U. Hartl (Park et al, 2013)	
**Antibodies**		
Goat anti-mCherry (1:300)	Origene	Cat# AB0040-200
Rabbit anti-cleaved caspase-3 (1:400)	Cell Signaling Technologies	Cat# 9662
Mouse anti-EM48 (1:300)	Merck	Cat# MAB5374
Chicken anti-MAP2 (1:500)	Novus Biological	Cat# NB300-213
Mouse anti-His (1:300)	Abcam	Cat# ab18184
Mouse anti-NeuN (1:400)	Thermo Fisher Scientific	Cat# MA5-33103
Rabbit anti-DARPP-32 (WB- 1:800 / IHC 1:400)	Abcam	Cat# ab40801
Chicken anti-GFAP (1:500)	Origene	Cat# AP31806PU-N
Goat anti-Iba1 (1:300)	Abcam	Cat# ab107159
Mouse anti-VGlut1 (1:300)	Abcam	Cat# ab134283
Rabbit anti-PSD-95 (1:300)	GeneTex	Cat# GTX133091
Rabbit anti-PDE10A (1:1000)	Abcam	Cat# ab227829
Rabbit anti-HTT (1:1000/1:5000)	Cell Signaling Technologies	Cat# 5656S
Mouse anti-α-Tubulin (1:2000)	Sigma-Aldrich	Cat# T9026
Mouse anti-PolyQ (1:50)	Merck	Cat# MAB1574
Mouse anti-β-Actin (1:5000)	Abcam	Cat# 8226
Rabbit anti-Iba1 (1:500)	Abcam	ab178847
Donkey anti-goat, Cy3 (1:300)	Jackson Immunoresearch	RRID: AB_2340411
Donkey anti-rabbit, Alexa Fluor 647 (1:300)	Jackson Immunoresearch	RRID: AB_2492288
Donkey anti-mouse, Cy3 (1:300)	Jackson Immunoresearch	RRID: AB_2340813
Donkey anti-chicken, Alexa Fluor 647 (1:300)	Jackson Immunoresearch	RRID: AB_2340379
Donkey anti-mouse, Alexa Fluor 647 (1:300)	Jackson Immunoresearch	RRID: AB_2340862
Donkey anti-rabbit, Alexa Fluor 488 (1:300)	Jackson Immunoresearch	RRID: AB_2313584
Donkey anti-chicken, Alexa Fluor 488 (1:300)	Jackson Immunoresearch	RRID: AB_2340375
Goat anti-mouse, Alexa Fluor 488 (1:300)	Thermo Fisher Scientific	Cat# A-11029
Donkey anti-mouse HRP (1:2500)	Thermo Fisher Scientific	Cat# na931
Donkey anti-rabbit HRP (1:2500)	Thermo Fisher Scientific	Cat# na934
Donkey anti-chicken HRP (1:2500)	Thermo Fisher Scientific	Cat# SA1-300
**Oligonucleotides and other sequence-based reagents**		
R6/2 Fw primer	Eurofins Genomics	5’ CCGCTCAGGTTCT GCTTTTA 3’
R6/2 Rv primer	Eurofins Genomics	5’ TGGAAGGACTTGA GGGACTC 3’
zQ175DN Fw primer	Eurofins Genomics	5’ GCGGGCTTATAC CCCTACAG 3’
zQ175DN Rv primer	Eurofins Genomics	5’ TCCAGGACAGCC AGAGCTAG 3’
**Chemicals, Enzymes and other reagents**		
Taq DNA Polymerase	New England Biolabs	Cat# M0267S
Proteinase k	Sigma-Aldrich	Cat# P6556
ROCK inhibitor	Abcam	Cat# ab143784
Ketamine (100 mg/kg)	Medistar	
Xylazine (20 mg/kg)	Medistar	
NeuroTrace 640/660 (1:300)	Thermo Fisher Scientific	Cat# N21483
DAPI (0.5 µg/ml)	Thermo Fisher Scientific	Cat# 62248
Hoechst 33342 (2 µg/ml)		
DiIC18	Thermo Fisher Scientific	Cat# D282
MG-132	Merck	Cat# M7449
**Software**		
ImageJ/Fiji v 1.54r	Schindelin et al, 2012	
Leica Application Suite X v 3.5.7.23225 software	Leica	
EthoVision XT 16 software	Noldus Information Technology	
FastQC v0.12.1	Babraham Informatics	
**Other**		
Protein Dye Assay kit	Bio-Rad	Cat # 500-0006EDU
HRP substrate Clarity Max Western ECL Substrate kit	Bio-Rad	Cat# 170-5062
Lexogen SPLIT RNA Extraction kit	Lexogen	
DNF-471 RNA kit	Agilent	
QuantSeq 3’ mRNA-Seq Library Prep kit FWD V2	Lexogen	
ChemiDoc MP Imaging System	Bio-Rad	Cat# 1200-3154
TriStar^2^ S LB 942 microplate reader	Berthold Technologies	
Rota-Rod NG system	Ugo Basile	Cat# 47650
BIO-GS3 Grip Test	Bioseb	
STELLARIS 5 confocal microscope	Leica	
Thunder Imager 3D Tissue fluorescent microscope	Leica	
Axio Imager Z1 microscope	Zeiss	
Perimax 12 peristaltic pump	SPETEC	Cat# 43012281711
Nanodrop2000c	Thermo Fisher Scientific	
VT1000S vibratome	Leica	Cat# 1404723512
Illumina NovaSeq X	Illumina	

### Plasmids

For transfection, the following plasmids were used: pcDNA3.1 mCherry, pcDNA3.1 HTT-Exon1-Q25-mCherry-myc-His and pcDNA3.1 HTT-Exon1-Q97-mCherry-myc-His (Hipp et al, 2012); pcDNA3.1 HTT-Exon1-Q25-His and pcDNA3.1 HTT-Exon1-Q72-His (Jeong et al, 2011; Voelkl et al, 2023); and NES-Fluc-EGFP (Park et al, 2013). The sequence of HTT-Exon1-Q25-mCherry-myc-His and HTT-Exon1-Q97-mCherry-myc-His and the sequence of HTT-Exon1-Q25-His and HTT-Exon1-Q72-His, are identical apart from the polyQ length.

### Mouse lines

All animal experiments were approved by the Government of Upper Bavaria (permit numbers ROB-55.2-2532.Vet_02-20-05 and ROB-55.2-2532.Vet_02-19-83) and LAVE North Rhine-Westphalia (permit numbers 81-02.04.2022.A399, 2025-163-Grundantrag and 2024-81_4-Grundantrag) and conducted in accordance with the relevant guidelines and regulations. Mice were housed under specific-pathogen-free conditions and ad libitum access to food and water. Transgenic R6/2 mice (Mangiarini et al, 1996) (JAX stock #002810) were maintained by breeding hemizygous R6/2 males with the F1 female progeny of the cross between CBA (Janvier Labs) and C57BL/6 (Janvier Labs) mice. Knock-in zQ175DN mice (Menalled et al, 2012; Southwell et al, 2016) (JAX stock #029928) were maintained on a C57BL/6 background. Anle138b treatment was administered orally, as food pellets (2 g/kg; Ssniff Spezialdiäten). Control groups were administered food pellets of the same composition, but without anle138b (placebo). The size of experimental groups was calculated by power analysis. For all experiments, study groups were assigned randomly before birth, mice of either sex were used, and experimenters were blind to the treatment groups. R6/2 and zQ175DN CAG repeat tract length was determined from ear biopsies by Transnetyx® and amounted to 206 ± 10 and 162 ± 35 repeats, respectively.

### Anle138b and BSA complexation

For application in an aqueous medium, a 10 mM stock solution of anle138b in dimethyl sulfoxide (DMSO) was prepared. Next, anle138b in DMSO was dissolved in an equimolar amount of bovine serum albumin (BSA; Roth) in phosphate buffer saline (PBS) to a final theoretical concentration of 200 µM. The final concentration of the anle138b/BSA complex was determined by high-performance liquid chromatography to be 165 µM.

### Primary neuronal cultures

Primary cortical neurons were prepared from E15.5 CD-1 wild-type mouse embryos of either sex. Pregnant female mice were sacrificed by cervical dislocation, the uterus was removed from the abdominal cavity, and the embryos were harvested and decapitated in a sterile Petri dish with ice-cold dissection medium, consisting of Hanks’ balanced salt solution (HBSS; Invitrogen) supplemented with 0.01 M HEPES, pH 7.4, 0.01 M MgSO_4_, and 1% penicillin/streptomycin (Invitrogen). The brain was removed from the skull, the hemispheres separated, the meninges removed, and the cortices isolated. The individual cortices were digested with 0.25% trypsin containing 1 mM ethylenediaminetetraacetic acid (EDTA) and 15 μl 0.1% DNAse I for 20 min at 37 °C. The digestion was stopped by washing with Neurobasal medium (Invitrogen) containing 5% fetal bovine serum, and cells were dissociated by triturating the tissue in prewarmed Neurobasal medium. Cells were centrifuged at 130×*g* for 5 min, the supernatant was removed, and the pellet resuspended in Neurobasal medium supplemented with 2% B27 (Invitrogen), 1% L-glutamine (Invitrogen) and 1% penicillin/streptomycin. The neurons were then plated in 24-well plates on 13 mm sterile coverslips coated with 50 µg/ml poly-D-Lysine (Sigma) and 1 µg/ml laminin (Thermo Fisher Scientific) at a density of 120,000 neurons per coverslip.

### Transfection of primary neurons

Neurons were transfected at day in vitro (DIV) 7 using the NeuroMag Transfection Reagent (OZ Biosciences), following the manufacturer’s instructions. Briefly, the transfection solution was prepared by adding 1 µg of NeuroMag reagent per µg of DNA to 50 µL of prewarmed Neurobasal medium containing 1 µg of DNA per construct. The mixture was incubated at room temperature (RT) for 20 min and added to the neurons in a 24-well plate. The cultures were placed on a magnetic plate at 37 °C, 5% CO_2_ for 15 min. Finally, the magnetic plate was removed, and the neurons were kept at 37 °C, 5% CO_2_ for protein expression.

### Anle138b treatment and proteasome inhibition in primary neurons

Neurons were treated at DIV 7 + 1 with 7 µM anle138b/BSA in PBS:DMSO (3:1) or DMSO as a control, for 24 h at 37 °C, 5% CO_2_. For proteasome inhibition, neurons were treated at DIV 7 + 2 with 5 µM MG-132 or DMSO as a control, for 4 h at 37 °C, 5% CO_2_.

### Immunocytochemistry on primary neurons

Transfected neurons were fixed at DIV 7 + 2 with 4% paraformaldehyde (PFA; Electron Microscopy Sciences) in PBS for 15 min at RT. Remaining free PFA groups were quenched with 50 mM ammonium chloride in PBS for 10 min at RT. Neurons were rinsed once with PBS, permeabilized with 0.25% Triton X-100 (Merck) in PBS for 5 min and washed 3 × 5 min with PBS. Following the washes with PBS, neurons were incubated in blocking solution consisting of 2% BSA, 4% donkey serum (Jackson Immunoresearch), and 0.01% NaN_3_ in PBS for 30 min at RT. After blocking, coverslips were transferred to a light-protected humid chamber and incubated with primary antibodies diluted in blocking solution for 1 h. The following primary antibodies were used: goat anti-mCherry (1:300; AB0040-200, Origene), rabbit anti-cleaved caspase-3 (1:400; 9662, Cell Signaling Technologies), mouse anti-EM48 (1:300; MAB5374, Merck), chicken anti-MAP2 (1:500; NB300-213, Novus Biological) and mouse anti-His (1:300; ab18184, Abcam). Neurons were washed 3 × 10 min with PBS at RT and incubated for 30 min with Alexa Fluor 488, Alexa Fluor 647 and Cyanine Cy3 conjugated secondary antibodies, derived from donkey (1:300; Jackson Immunoresearch) and 0.5 µg/ml DAPI in blocking solution. Neurons were washed 2 × 10 min with PBS, rinsed once with Mili-Q water and mounted on Menzer glass slides (VWR) using Prolong Glass Antifade mounting medium (Invitrogen).

Images were acquired using a Thunder Imager 3D Tissue fluorescent microscope (Leica) and analyzed using the ImageJ/Fiji software (Schindelin et al, 2012). For neuronal viability analysis, neurons with a negative cleaved caspase-3 signal were considered to be viable. For the analysis of Fluc-EGFP foci-containing cells, a value of two standard deviations above the foci/cytoplasm intensity ratio was considered as the threshold for the presence of foci. For all analyses, *n* = 4–6 independent experiments and 20–35 cells were considered per condition. Whenever possible, the experimenter was blind to the treatment groups.

### Behavioral tests and life span analysis

All behavioral analyses were performed during the mice’s dark phase, and habituation to the testing room was done for a period of 30–60 min prior to any testing. For the grip strength test, a total of three trials were performed per mouse. For each trial, forelimb strength was measured with the bar version of the BIO-GS3 Grip Test (Bioseb). The final score was the average of the three trials. Rotarod performance was analyzed using the Rota-Rod NG system (Ugo Basile), and it consisted of two training days followed by a test day. During training, the mice were placed on the rotating rod at 5 rotations per minute (rpm) for 5 min. For the test, a speed gradient of 5–40 rpm was implemented, and the latency to fall was measured up to a limit of 5 min. Three trials were performed, and the results were averaged to obtain the final score. The open field test consisted of a single trial in which mice were recorded in an open arena (40 × 40 × 40 cm) with black walls, white floor and illuminated homogeneously from above. Mice were recorded for 15 min and the distance traveled was calculated automatically using the EthoVision XT 16 software (Noldus Information Technology). For limb clasping, mice were recorded for 20 s while held by their tail, ~30 cm above a bench top. Limb clasping was assessed on three consecutive days, and the time spent clasping with either fore-, hind- or fore- and hindlimbs was manually recorded. The final value was the average of the three trials. Life span was determined by monitoring mouse burden according to a score sheet based on appearance, body weight and rearing reflex on a daily basis. Mice with severe burden were considered to have reached the experimental endpoint.

### Immunohistochemistry

Mice were deeply anesthetized with 1.6% ketamine/0.08% xylazine and transcardially perfused with PBS followed by 4% PFA in PBS. Brains were extracted from the skull and post-fixed in 4% PFA in PBS overnight at 4 °C. Fixed brains were sectioned into 30 µm thick coronal sections using a VT1000S vibratome (Leica). Floating sections were permeabilized with 0.5% Triton X-100 in PBS for 30 min at RT. Following permeabilization, staining against the VGlut1 and PSD-95 markers required antigen retrieval with 20 µg/ml Proteinase K (Sigma-Aldrich) in 50 mM Tris Base, 1 mM EDTA and 0.5% Triton X-100 in H_2_O for 10 min at 37 °C. Sections were washed once with PBS for 10 min at RT and incubated in blocking solution consisting of 0.2% BSA, 5% donkey serum, 0.2% L-Lysine (Sigma-Aldrich), 0.2% Glycine (Sigma-Aldrich), and 0.01% NaN_3_ in PBS for 1.5 h at RT. After blocking, sections were incubated with primary antibodies diluted in 2% BSA, 0.3% Triton X-100, and 0.01% NaN_3_ in PBS at 4 °C overnight. The following primary antibodies were used: mouse EM48 (1:300; MAB5374, Merck), chicken anti-MAP2 (1:500; NB300-213, Novus Biological), mouse anti-NeuN (1:400; MA5-33103, Thermo Fisher Scientific), rabbit anti-DARPP-32 (1:400; ab40801, Abcam), chicken anti-GFAP (1:500; AP31806PU-N, Origene), goat anti-Iba1 (1:300, ab107159, Abcam), mouse anti-VGlut1 (1:300; ab134283, Abcam) and rabbit anti-PSD-95 (1:300; GTX133091, GeneTex). Sections were washed 3 ×10 min with PBS at RT and incubated for 1 h at RT with Alexa Fluor 488, Cyanine Cy3 and/or Alexa Fluor 647 conjugated secondary antibodies, derived from donkey (1:300; Jackson Immunoresearch), NeuroTrace 640/660 (1:300; Thermo Fisher Scientific) and 0.5 µg/ml DAPI in 0.3% Triton X-100, 3% donkey serum and 0.01% NaN_3_ in PBS. Sections were washed 3 × 10 min with PBS and mounted on Menzer glass slides using Prolong Glass Antifade mounting medium.

Images were acquired using a STELLARIS 5 confocal microscope (Leica) and analyzed using the ImageJ/Fiji software. For the quantification of the forebrain area, a region of interest (ROI) was manually drawn around the forebrain. The volume of the striatum, motor cortex, ventricles and whole brain was analyzed according to the principle of Cavalieri, as previously described (Cyr et al, 2005) (volume = s_1_d_1_ + s_2_d_2_ + … s_n_d_n_, where s = surface area and d = distance between sections). For volume estimation, nine sections spaced 200 µm apart were used. Reference anatomical landmarks, such as the anterior commissure and the corpus callosum, were used to choose the starting sections and verify the consistency of the final sections (Bregma = 1.045 mm to Bregma = −0.755 mm). The boundaries of the individual brain regions were defined by anatomical landmarks identifiable by NeuN staining according to the Allen Brain Atlas (mouse.brain-map.org). Immunoreactivity was analyzed in the striatum and motor cortex (Bregma = 0.345 ± 0.1 mm) by identifying these areas, manually drawing an ROI and measuring their size. Background correction was used when necessary, and the area occupied by the appropriate immunofluorescent signal (GFAP or Iba1) was quantified by adequate thresholding and morphological filtering. The percentage of the ROI area occupied by immunoreactive species was determined by calculating the ratio between the two areas. For each mouse, two brain sections were analyzed. The percentage of inclusion-bearing neurons was analyzed in the striatum and motor cortex (Bregma = 0.345 ± 0.1 mm) by counting the mHTT inclusions overlapping with nuclear DAPI (nuclear inclusions) or Neurotrace dye signal (cytoplasmic inclusions). For each mouse, 10 fields of view (FOVs), per section, and two brain sections were analyzed. For the quantification of excitatory synapses in the dorsal striatum (Bregma = −0.2 ± 0.1 mm), images of pre- and postsynaptic immunostainings (VGlut1 and PSD-95, respectively) were superimposed using the ImageJ “Colocalization Threshold” feature. Puncta smaller or bigger than 2 or 10 pixels, respectively, and puncta with a circularity smaller than 0.8 were excluded from the analysis. The resulting colocalization map was isolated, and the number of puncta quantified using the ImageJ “Analyze Particles” feature. For each mouse, two brain sections and 10 FOVs per section were analyzed. Whenever possible, the experimenter was blind to the treatment groups.

### Dendritic spine labeling

Fixed brain sections were labeled with the lipophilic dye 1,1’-dioctadecyl-3,3,3’,3’-tetramethylindocarbocyanine perchlorate (DiIC18; Thermo Fisher Scientific) as previously described (Speranza et al, 2022). Briefly, sections were placed on a glass slide, the PBS was removed, and DiIC18 crystals were applied directly into the striatum. For this, the tip of a 27-gauge needle was coated with DiIC18 and the crystals were slowly deposited in the regions of interest, taking care to avoid excessive amounts of crystals in the same area. The sections were covered with PBS to avoid dehydration and incubated in the dark for 15 min, at RT. Next, the sections were transferred to a 24-well plate and incubated in PBS at 4 °C, protected from light, for 3 days. For combined immunofluorescence, the sections were permeabilized with 100 µg/mL Saponin (Thermo Fisher Scientific), 3% BSA in PBS at RT, for 30 min. After permeabilization, sections were incubated with primary antibodies diluted in 100 µg/mL Saponin, 3% BSA and 0.01% NaN_3_ in PBS at 4 °C, o/n. The following primary antibodies were used: chicken anti-MAP2 (1:500; NB300-213, Novus Biological) and rabbit anti-DARPP-32 (1:400; ab40801, Abcam). Sections were washed 3 × 10 min with PBS at RT and incubated for 3 h at RT with Alexa Fluor 488 and Alexa Fluor 647 conjugated secondary antibodies, derived from donkey (1:300; Jackson ImmunoResearch) and 0.5 µg/ml DAPI in 3% BSA and 0.01% NaN_3_ in PBS. Finally, sections were washed 3 × 10 min with PBS and mounted on Menzer glass slides using Prolong Glass Antifade mounting medium.

Images were acquired using a STELLARIS 5 confocal microscope and analyzed using the ImageJ/Fiji software. For dendritic spine density analysis in the dorsal striatum (Bregma = -0.2 ± 0.1 mm), an ROI was manually drawn around DiIC18-labeled dendrites, and their length was measured. All lateral protrusions in the ROI were semi-automatically detected using the ImageJ plugin “Dendritic Spine Counter” and spine density expressed as the number of spines per dendritic length (n/µm). For each mouse, 10–28 dendrites were analyzed, and 20 µm of each dendrite were considered for analysis. Whenever possible, the experimenter was blind to the treatment groups.

### Western blot and filter trap assay of brain tissue lysates

Mice were sacrificed by cervical dislocation, brains extracted from the skull and the striatum and motor cortex dissected on ice. Brain regions were homogenized on ice in lysis buffer (50 mM Tris buffer, pH 7.4, 150 mM NaCl, 2 mM EDTA, 1% Triton X-100, phosphatase inhibitor (Roche) and protease inhibitor cocktail (Roche)). The resulting lysates were centrifuged at 15,000 × *g* for 15 min at 4 °C, the pellet discarded, and total protein concentration was determined using the Protein Dye Assay kit (Bio-Rad). For Western blots, samples containing 20 µg of total protein were denatured at 95 °C for 5 min and separated according to their size on 10% SDS-PAGE gels. The proteins were transferred onto polyvinylidene fluoride (PVDF) membranes (Bio-Rad), using a Trans-Blot Turbo transfer system (Bio-Rad), and unspecific binding sites were blocked by incubating the membranes in 5% dried milk (Milipore), 3% BSA in 0.1% Tris-buffered saline with 0.1% Tween 20 (ITW Reagents) (TBS-T) for 2 h at RT. After blocking, the membranes were incubated with primary antibodies diluted in 3% BSA and 0.01% NaN_3_ in TBS-T, overnight at 4 °C. The following primary antibodies were used: rabbit anti-PDE10A (1:1,000; ab227829, Abcam), rabbit anti-DARPP-32 (1:800; ab40801, Abcam), rabbit anti-HTT (1:1,000; 5656S, Cell Signaling Technologies), and mouse anti-α-Tubulin (1:2,000; T9026, Sigma-Aldrich). Membranes were washed 3 × 10 min in TBS-T at RT and incubated for 2 h at RT with horse-radish peroxidase (HRP) conjugated secondary antibodies, derived from donkey (1:2500; Thermo Fisher Scientific), diluted in 3% dried milk and 0.01% NaN_3_ in TBS-T. Proteins were visualized by chemiluminescence by adding the HRP substrate Clarity Max^TM^ Western ECL Substrate (Bio-Rad) to the membranes. Images were acquired using the FUSION FX system (Vilber).

For filter trap assays, tissue samples were centrifuged at 2000 × *g* for 10 min at 4 °C, the pellet discarded, and total protein concentration calculated. Filter trap assays were performed with 35 µg of total protein diluted in 100 µL of lysis buffer. Cellulose acetate membranes (0.2 µm pore size, Bio-Rad) and filter paper (Bio-Rad) were pre-equilibrated in 0.1% SDS in H_2_O for 10 min at RT and assembled in the slot blot apparatus (Bio-Dot SF, Bio-Rad). Samples were loaded and allowed to completely pass through the membrane under vacuum. Wells were washed 3x with 100 µL 0.1% SDS in H_2_O, followed by standard immunoblotting of the membranes. For the detection of aggregated mHTT, mouse EM48 (1:1000; MAB5374, Merck) primary antibody and HRP-conjugated secondary antibody (1:2500; Thermo Fisher Scientific) were used. Samples were visualized by chemiluminescence by adding the HRP substrate Clarity Max^TM^ Western ECL Substrate to the membranes. Images were acquired using the FUSION FX system.

For each experiment, three to four biological replicates per genotype and treatment group were analyzed. One technical replicate was performed for each mouse.

### RNA sequencing

RNA extraction, library preparation, sequencing and initial data processing were performed by Lexogen NGS Services, Lexogen GmbH, Austria. In brief, RNA was isolated using Lexogen’s SPLIT RNA Extraction Kit. Samples were characterized by UV-Vis spectrophotometry (Nanodrop2000c, Thermo Fisher), and RNA integrity was assessed on a Fragment Analyzer System using the DNF-471 RNA Kit (15 nt) (Agilent). The libraries were constructed using Lexogen’s QuantSeq 3’ mRNA-Seq Library Prep Kit FWD V2. Prepared libraries were quality controlled on a Fragment Analyzer System using the DNF-474 using the HS-DNA kit (1–6000 bp) (Agilent). cDNA libraries were sequenced with an Illumina NovaSeq X platform using 100 nt single end read length, with five million reads on average per sample. Sequencing quality control of the raw reads was assessed using FastqQC software, and adapter sequences were removed with cutadapt (Martin, 2011). Alignment to the *Mus musculus* reference genome (GRCm38, Ensembl) and read counting were performed using STAR (Dobin et al, 2013) and featureCounts (Liao et al, 2014). The DESeq2 R package (Love et al, 2014) was used to perform differential gene expression analysis. Genes were considered differentially expressed with an adjusted *p* value lower than 0.05.

### iPSC culture and differentiation into NPCs

The HD Q71-iPSC and HD Q180-iPSC (ND36999) lines were kindly provided by George Q. Daley and the NINDS iPSC Repository through the Coriell Institute, respectively. Corrected isogenic counterparts of Q180-iPSCs were kindly provided by M. A. Pouladi (Xu et al, 2017). The HD Q71-iPSC line was established and characterized for pluripotency (Park et al, 2008). iPSCs were maintained on Geltrex (ThermoFisher Scientific) using mTeSR1 media (Stem Cell Technologies) at 37 °C, 5% CO_2_. The absence of mycoplasma was confirmed by testing the iPSC lines for mycoplasma contamination at least once every 2 weeks. Neural differentiation was induced with STEMdiff Neural Induction Medium (Stem Cell Technologies) after the monolayer culture method (Chambers et al, 2009). In brief, iPSCs were washed once with PBS, followed by the addition of 1 ml of Gentle Dissociation Reagent (Stem Cell Technologies). After 10 min incubation, the cells were gently collected, and 2 ml of DMEM/F12 (Thermo Fisher Scientific) containing 10 μM ROCK inhibitor (Abcam) was added. The cells were then centrifuged at 300 × *g* for 10 min and resuspended in STEMdiff Neural Induction Medium supplemented with 10 μM ROCK inhibitor. Cells were seeded on plates coated with 15 μg/ml poly-ornithine and 10 μg/ml laminin at a density of 200,000 cells/cm^2^ for neural differentiation.

### Anle138b treatment and proteasome inhibition in NPCs

NPCs were treated with 7 μM anle138b/BSA for 16 h. The following day, cells were treated with fresh 7 μM anle138b/BSA for 8 h in the presence of 5 μM MG-132 for proteasome inhibition or DMSO as a control treatment.

### Immunocytochemistry in NPCs

NPCs were fixed with 4% PFA in PBS for 20 min at RT. Cells were rinsed once with PBS, permeabilized with 0.2% Triton X-100 in PBS for 10 min and incubated in a 3% BSA, 0.2% Triton X-100 in PBS blocking solution for 10 min at RT. After blocking, NPCs were incubated with the mouse anti-PolyQ (1:50; MAB1574, Merck) primary antibody diluted in blocking solution for 2 h at RT. Cells were washed once with 0.2% Triton X-100 in PBS at RT and incubated for 1 h with a goat anti-mouse Alexa Fluor 488 secondary antibody (1:500; A-11029, Thermo Fisher Scientific) and 2 µg/ml Hoechst 33342 (1656104, Life Technologies) in blocking solution. NPCs were washed once with 0.2% Triton X-100 in PBS, once with dH_2_O, once with EtOH, and mounted using FluorSave mounting medium (Merck).

Images were acquired using an Axio Imager Z1 microscope (Zeiss) and analyzed using the ImageJ/Fiji software. For all analyses, *n* = 3 independent experiments and 20 cells were considered per condition. Whenever possible, the experimenter was blind to the treatment groups.

### Western blot and filter trap assay in NPCs

Cells were collected and lysed in a non-denaturing lysis buffer (50 mM Hepes, pH 7.4, 150 mM NaCl, 1 mM EDTA, and 1% Triton X-100) supplemented with 2 mM sodium orthovanadate, 1 mM phenylmethylsulfonyl fluoride, and protease inhibitor mix on ice. Lysates were homogenized through a syringe needle (27-gauge). Standard BCA protein assay was used to determine protein concentration. After equilibration of protein concentration, the equilibrated whole lysates were centrifuged at 8000 × *g* for 5 min at 4 °C. Supernatant and pellet were separated for SDS-PAGE and filter trap analysis. The supernatant was used for SDS-PAGE analysis, transferred to PVDF membranes (Millipore), and subjected to immunoblotting. The following antibodies were used: anti-β-Actin (1:5000; 8226, Abcam) and anti-HTT (1:1000; ab5656, Cell Signaling Technologies). For the filter trap, the pellets were resuspended in 2% SDS and loaded onto a cellulose acetate membrane assembled in a slot blot apparatus. The cellulose acetate membrane was rinsed with 0.2% SDS, and retained SDS-insoluble mHTT aggregates were detected with mouse anti-polyQ antibody (1:5000; MAB1574, Millipore).

### Statistical analysis

Statistical analysis and graphical representations were performed in GraphPad Prism^TM^ v 10.2.3 (GraphPad Software Inc.). Bar graphs show the mean and standard deviation. Violin plots show the median, density curves and interquartile range. The significance level was set to *p* < 0.05, the statistical tests and group numbers are detailed in the figure legends.

## Supplementary information

Appendix

Peer Review File

Source data Fig. 1

Source data Fig. 2

Source data Fig. 3

Source data Fig. 4

Source data Fig. 5

Source data Fig. 6

Source data Fig. 7

Source data Fig. 8

Figure EV1 Source Data

Figure EV2 Source Data

Figure EV3 Source Data

Figure EV4 Source Data

Figure EV5 Source Data

Appendix Figure S1 Source Data

Appendix Figure S2 Source Data

Appendix Figure S3 Source Data

Appendix Figure S5 Source Data

Expanded View Figures

## Data Availability

The RNA-seq dataset generated in this study is available in the following database: Sequence read archive (SRA), PRJNA1429108 The source data of this paper are collected in the following database record: biostudies:S-SCDT-10_1038-S44321-026-00459-9.
