# Supplementary material for: Anle138b ameliorates pathological phenotypes in mouse and cellular models of Huntington’s disease
Source: EMBO Mol Med. 2026 Jun 26;18(7):2838–66. doi: 10.1038/s44321-026-00459-9 (PMC13365221; doi:10.1038/s44321-026-00459-9)
Supplement: Supplementary file 1 — Appendix [file 44321_2026_459_MOESM1_ESM.pdf]

Appendix

Anle138b ameliorates pathological phenotypes in mouse and cellular models of Huntington's disease

Miguel da Silva Padilha[1,2,3], Seda Koyuncu[3,6], Evangeline Chabanis[1], Sergey Ryazanov[4], Andrei Leonov[4], David Vilchez[3,5,6], Rüdiger Klein[2], Armin Giese[7], Christian Griesinger[4,8] and Irina Dudanova[1,2,3,9]

[1]Center for Anatomy, Faculty of Medicine and University Hospital Cologne, University of Cologne, Cologne, Germany;

[2]Department of Molecules – Signaling – Development, Max Planck Institute for Biological Intelligence, Martinsried, Germany

[3]Cologne Excellence Cluster on Cellular Stress Responses in Aging-Associated Diseases (CECAD), University of Cologne, Cologne, Germany

[4]Department of NMR Based Structural Biology, Max Planck Institute of Multidisciplinary Sciences, Göttingen, Germany;

[5]Center for Molecular Medicine Cologne (CMMC), Faculty of Medicine and University Hospital Cologne, University of Cologne, Cologne, Germany

[6]Institute for Integrated Stress Response Signaling, Faculty of Medicine, University Hospital Cologne, Cologne, Germany

[7]MODAG GmbH, Wendelsheim, Germany;

[8]Cluster of Excellence "Multiscale Bioimaging: From Molecular Machines to Networks of Excitable Cells" (MBExC), University of Göttingen, Göttingen, Germany;

[9]Institute of Anatomy and Cell Biology, University of Würzburg, Würzburg, Germany

Correspondence: Irina Dudanova, irina.dudanova@uni-wuerzburg.de

Appendix Data: Contents

Appendix figure S1. Additional characterization of anle138b-treated R6/2 mice ...……………..…….. 3

Appendix figure S2. Sex-specific effect of anle138b on motor performance of R6/2 mice ……….…... 4

Appendix figure S3. Brain atrophy and mHTT inclusion bodies in anle138b-treated R6/2 mice …..... 6

Appendix figure S4. Transcriptional profiling in the striatum and motor cortex of zQ175DN mice ….. 7

Appendix figure S5. Anle138b ameliorates insoluble mHTT load in a second line of HD-iPSC derived NPCs ……………………………………………………………………………………………….. 8

Appendix Table S1. Summary of the effects of anle138b on HD-related phenotypes in the R6/2 and zQ175DN mouse models ………………………………………………………………………. 8

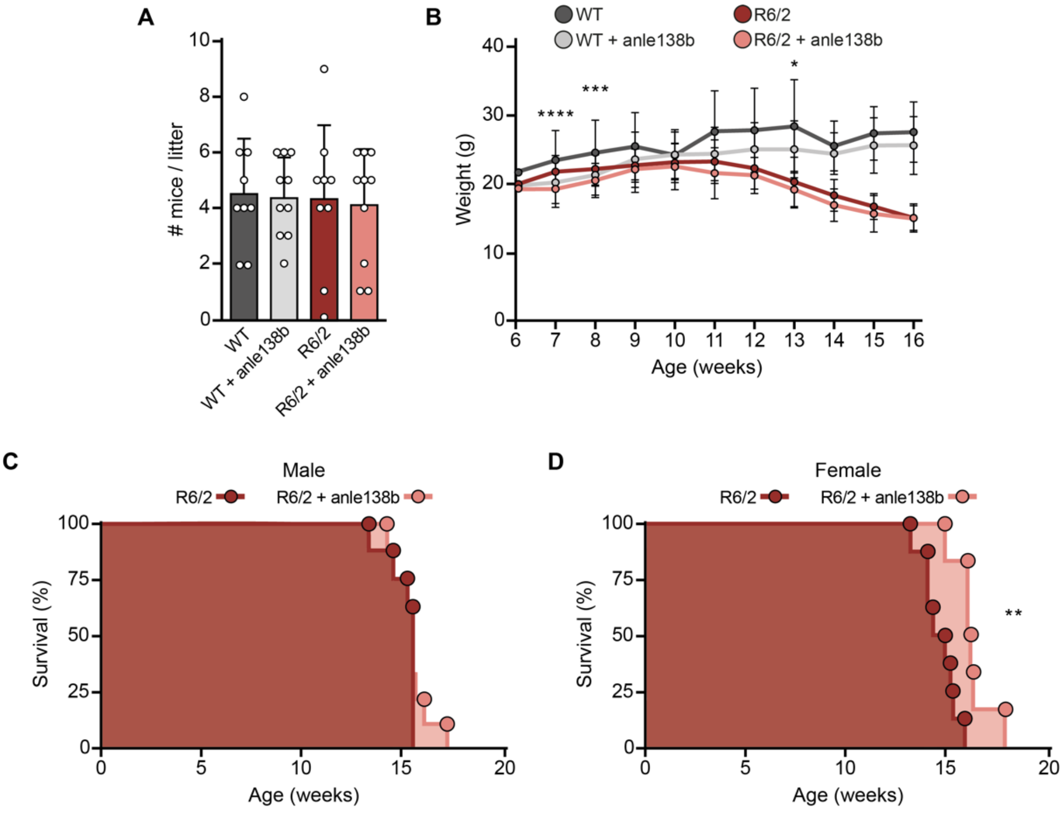

Appendix figure S1. Additional characterization of anle138b-treated R6/2 mice.
(A) Number of WT and R6/2 pups per litter for placebo or anle138b-treated breeding pairs. Two-way ANOVA, not significant. n = 9 – 10 litters per group. **(B)** Body weight of WT and R6/2 mice treated with placebo or anle138b. Repeated measures ANOVA. Significant differences between treatment groups are indicated on the graph for the respective time points: *$p < 0.05$; ***$p < 0.001$; ****$p < 0.0001$. n = 19 – 22 mice per group. **(C – D)** Kaplan-Meier survival curve for male (C) and female (D) R6/2 mice. The same data as shown in Fig. 2G, separated by sex. Log-rank test. Significant pairwise comparisons are indicated on the graphs. **$p < 0.01$. Source data for this figure is available online.

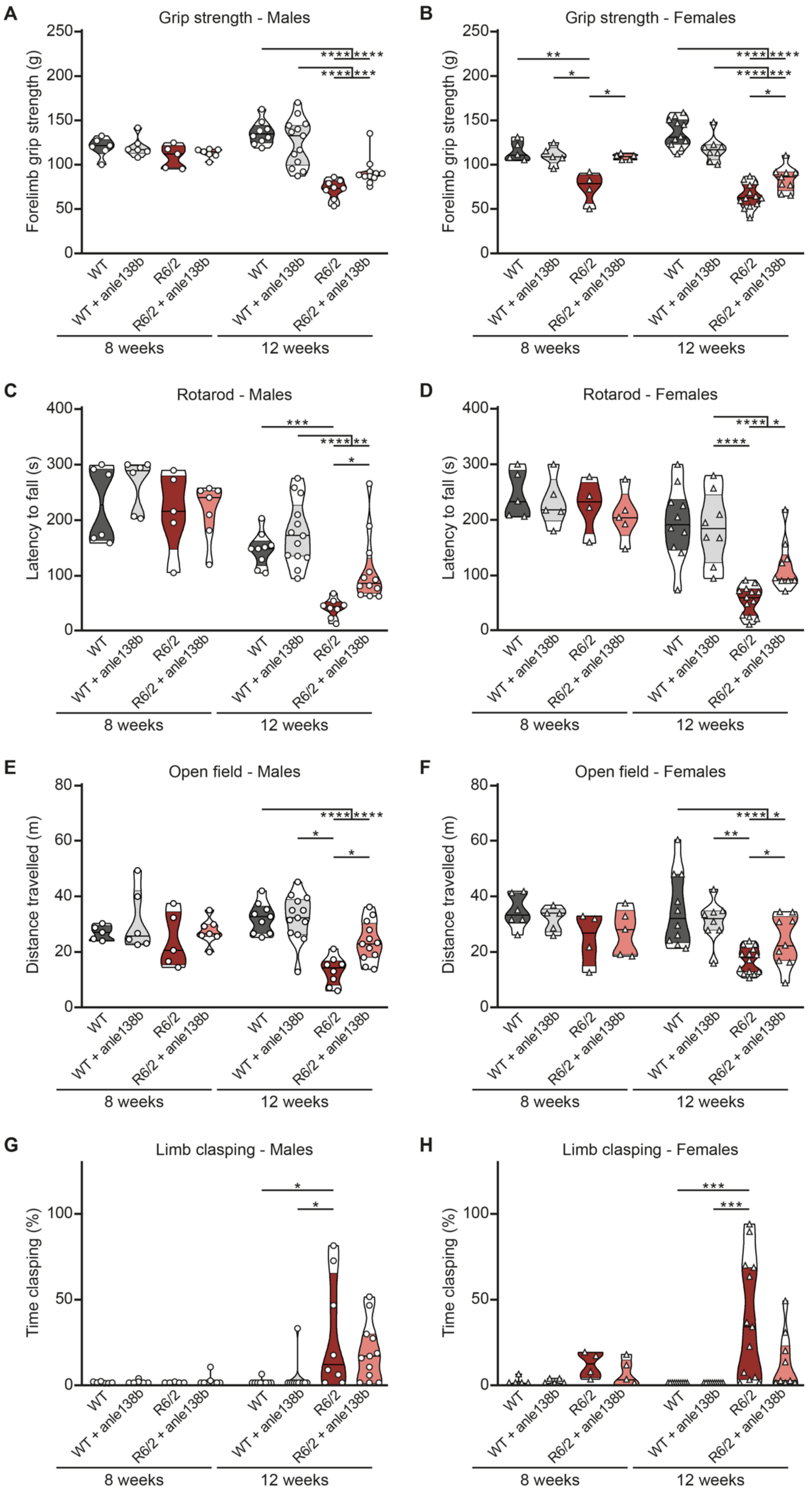

A
Grip strength - Males
Forelimb grip strength (g)
B
Grip strength - Females
C
Rotarod - Males
Latency to fall (s)
D
Rotarod - Females
E
Open field - Males
Distance travelled (m)
F
Open field - Females
G
Limb clasping - Males
Time clasping (%)
H
Limb clasping - Females
WT
WT + anle138b
R6/2
R6/2 + anle138b
8 weeks
12 weeks

Appendix figure S2. Sex-specific effect of anle138b on motor performance of R6/2 mice.
(A) Male mice forelimb grip strength. Two-way ANOVA with Bonferroni's multiple comparison test, per age group. 8 weeks: not significant; 12 weeks: Treatment, p = 0.4970; Genotype, ****p < 0.0001; Treatment x Genotype, *p = 0.0118. **(B)** Female mice forelimb grip strength. Two-way ANOVA with Bonferroni's multiple comparison test, per age group. 8 weeks: Treatment, p = 0.0907; Genotype, *p = 0.0114; Treatment x Genotype, *p = 0.0169.; 12 weeks: Treatment, p = 0.9688; Genotype, ****p < 0.0001; Treatment x Genotype, ***p = 0.0003. **(C)** Male mice latency to fall from the rotarod. Two-way ANOVA with Bonferroni's multiple comparison test, per age group. 8 weeks: not significant; 12 weeks: Treatment, **p = 0.0027; Genotype, ****p < 0.0001; Treatment x Genotype, p = 0.21196. **(D)** Female mice latency to fall from the rotarod. Two-way ANOVA with Bonferroni's multiple comparison test, per age group. 8 weeks: not significant; 12 weeks: Treatment, p = 0.1093; Genotype, ****p < 0.0001; Treatment x Genotype, *p = 0.0411. **(E)** Male mice distance travelled in the open field arena. Two-way ANOVA with Bonferroni's multiple comparison test, per age group. 8 weeks: not significant; 12 weeks: Treatment, *p = 0.0211; Genotype, ****p < 0.0001; Treatment x Genotype, *p = 0.0224. **(F)** Female mice distance travelled in the open field arena. Two-way ANOVA with Bonferroni's multiple comparison test, per age group. 8 weeks: Treatment, p = 0.9604; Genotype, *p = 0.0389; Treatment x Genotype, p = 0.4390 ; 12 weeks: Treatment, p = 0.6052; Genotype, ***p = 0.0001; Treatment x Genotype, p < 0.0569. **(G)** Male mice fraction of time spent clasping. Two-way ANOVA with Bonferroni's multiple comparison test, per age group. 8 weeks: not significant; 12 weeks: Treatment, p = 0.4853; Genotype, ***p = 0.0004; Treatment x Genotype, p = 0.3109. **(H)** Female mice fraction of time spent clasping. Two-way ANOVA with Bonferroni's multiple comparison test, per age group. 8 weeks: Treatment, p = 0.2995; Genotype, **p = 0.01; Treatment x Genotype, p = 0.2724.; 12 weeks: Treatment, p = 0.0613; Genotype, ***p < 0.0006; Treatment x Genotype, p = 0.0613. For all behavioral analyses, n = 5 – 7 males and 4 – 5 females (8 weeks) or 8 – 13 males and 8 – 13 females (12 weeks) per group. The same data as shown in Fig. 2B-C, E-F, separated by sex. Data presented as violin plots with median and interquartile ranges. Significant pairwise comparisons are indicated on the graphs. *p < 0.05, **p < 0.01, ***p < 0.001, ****p < 0.0001. Source data for this figure is available online.

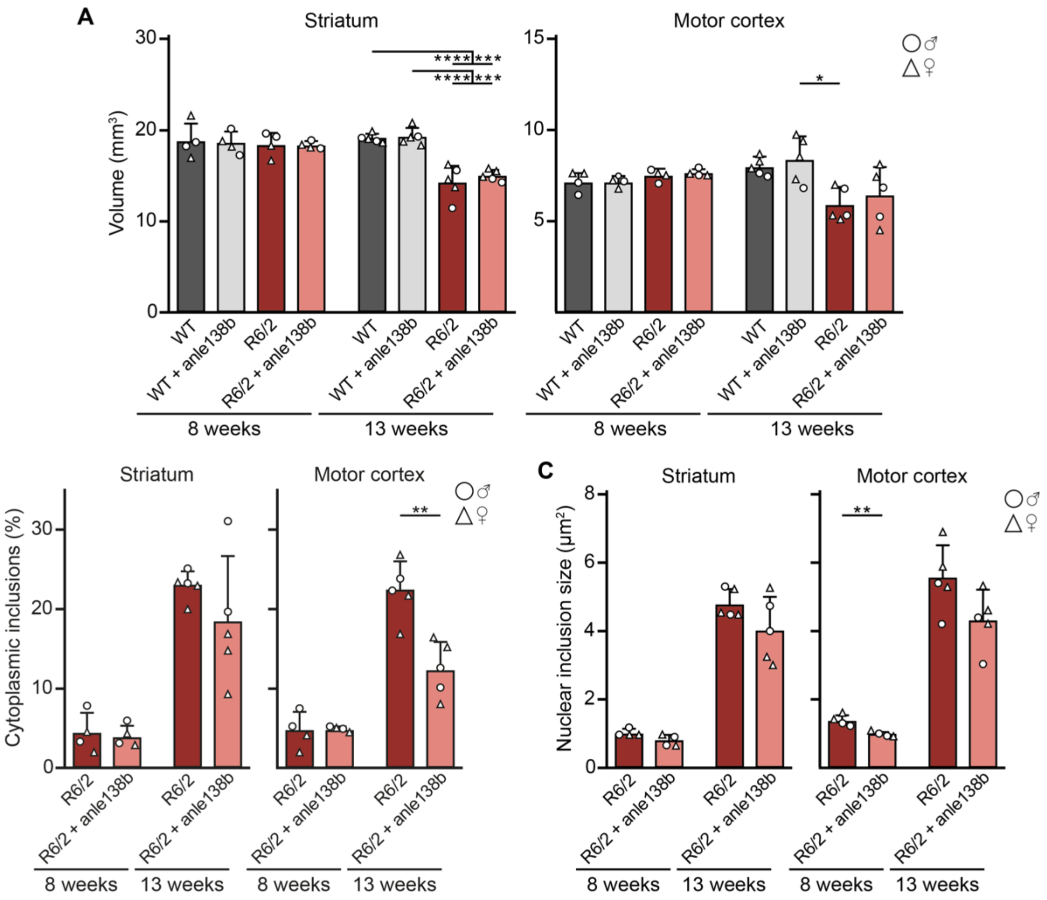

Appendix figure S3. Brain atrophy and mHTT inclusion bodies in anle138b-treated R6/2 mice.
(A) Striatum and motor cortex volume quantification in 8 and 13-week-old WT and R6/2 mice. Two-way ANOVA with Bonferroni's multiple comparison test. Striatum, 8 weeks: not significant; 13 weeks: Treatment, $p = 0.3682$; Genotype, ****$p < 0.0001$; Treatment x Genotype, $p = 0.5531$. Motor cortex, 8 weeks: not significant; 13 weeks: Treatment, $p = 0.4037$; Genotype, **$p < 0.0012$; Treatment x Genotype, $p = 0.9394$. n = 4 (8 weeks) or 5 mice (13 weeks) per group. **(B)** Quantification of the fraction of neurons with cytoplasmic mHTT inclusion bodies in the striatum and motor cortex of R6/2 mice. Unpaired two-tailed *t*-test, per age group and brain region. n = 4 (8 weeks) or 5 mice (13 weeks) per group. **(C)** Quantification of nuclear mHTT inclusion body size in the striatum and motor cortex of 8 and 13-week-old R6/2 mice. Unpaired two-tailed *t*-test, per age group and brain region. n = 4 (8 weeks) or 5 mice (13 weeks) per group. Data presented as mean ± SD. Significant pairwise comparisons are indicated on the graphs. *$p < 0.05$, **$p < 0.01$, ***$p < 0.001$, ****$p < 0.0001$. Source data for this figure is available online.

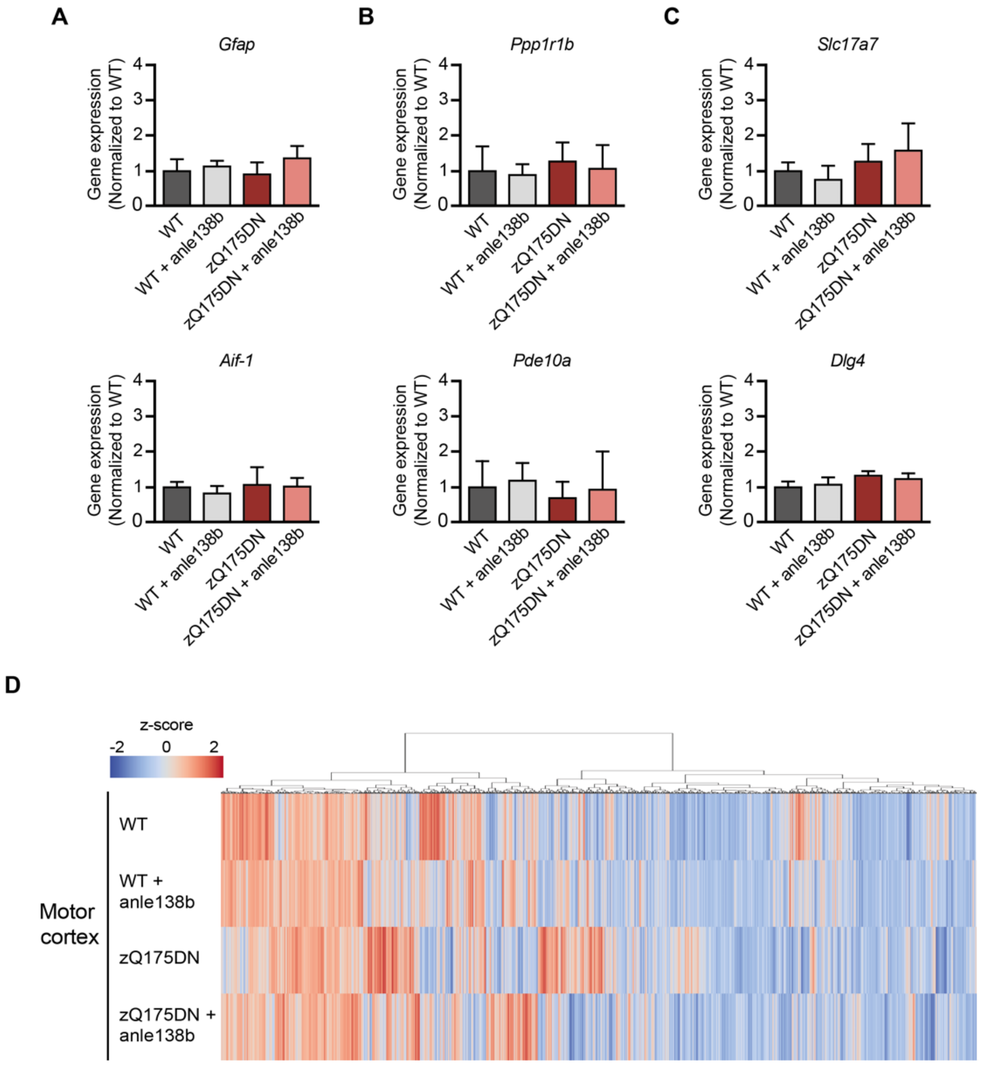

Appendix figure S4. Transcriptional profiling in the striatum and motor cortex of zQ175DN mice. **(A)** Expression of the *Gfap* and *Aif-1* genes encoding the glial markers GFAP and Iba1, respectively, in the striatum of 6-month-old WT and zQ175DN mice treated with placebo or anle138b. Two-way ANOVA with Bonferroni's multiple comparison test. *Gfap*: Treatment, p = 0.1175; Genotype, p = 0.6933; Treatment x Genotype, p = 0.3667; A*if-1*: Treatment, p = 0.5598; Genotype, p = 0.4359; Treatment x Genotype, p = 0.7398. **(B)** Expression of the *Ppp1r1b* and *Pde10a* genes, encoding the striatal markers DARPP-32 and PDE10A, respectively, in the striatum of 6-month-old WT and zQ175DN mice treated with placebo or anle138b. Two-way ANOVA with Bonferroni's multiple comparison test. *Ppp1r1b*: Treatment, p = 0.6416; Genotype, p = 0.5029; Treatment x Genotype, p = 0.8761; *Pde10a*: Treatment, p = 0.6663; Genotype, p = 0.5465; Treatment x Genotype, p = 0.9909. **(C)** Expression of the *Slc17a7* and *Dlg4* genes, encoding the synaptic proteins VGlut1 and PSD-95, respectively, in the striatum of 6-month-old WT and zQ175DN mice treated with placebo or anle138b. Two-way ANOVA with Bonferroni's multiple comparison test. *Slc17a7*: Treatment, p = 0.9513; Genotype, p = 0.0885; Treatment x Genotype, p = 0.3685; *Dlg4*: Treatment, p = 0.8719; Genotype, *p = 0.0274; Treatment x Genotype, p = 0.4041. **(D)** Heatmap showing differentially expressed genes in the motor cortex of 6-month-old WT and zQ175DN mice treated with placebo or anle138b. n = 3 - 7 mice per group. Data presented as mean ± SD. Source data for this figure is available online.

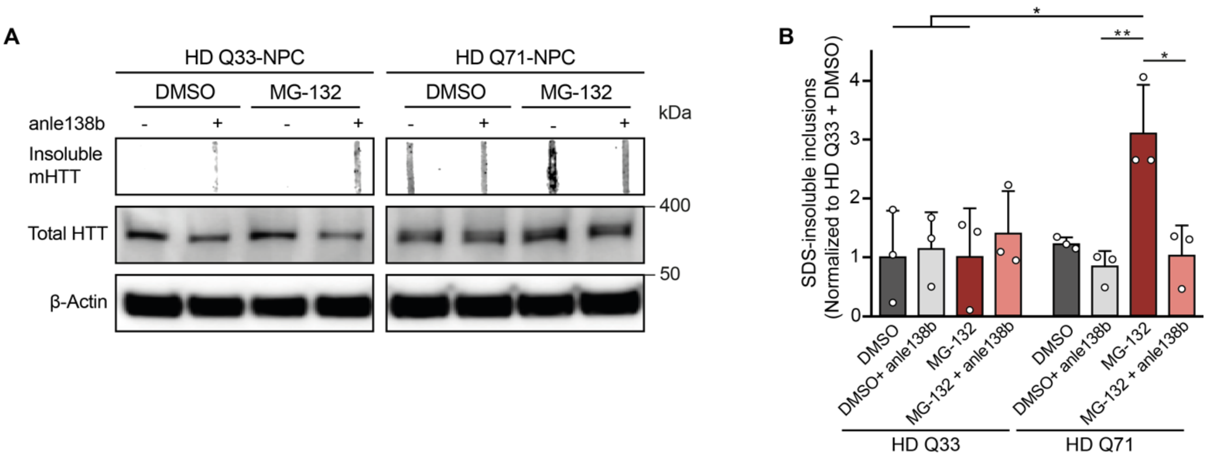

Appendix figure S5. Anle138b ameliorates insoluble mHTT load in a second line of HD-iPSC derived NPCs.
(A) Filter trap of HD Q71-NPCs and HD Q33-NPCs treated with vehicle (DMSO) or 7 µM anle138b. Total levels of HTT were determined by immunoblotting, β-Actin served as loading control. The order of the bands on the filter trap membrane was digitally rearranged to be the same as the order on the western blot membranes. **(B)** Quantification of insoluble mHTT. Values were normalized to vehicle-treated HD Q33-NPCs. Three-way ANOVA with Bonferroni's multiple comparison test. mHTT, $p = 0.1280$; MG-132, $*p = 0.0322$; anle138b, $p = 0.0752$; mHTT x MG-132, $p = 0.0931$; mHTT x anle138b, $**p = 0.0085$; MG-132 x anle138b, $p = 0.1808$; mHTT x MG-132 x anle138b, $p = 0.0808$. $n = 3$ independent experiments. Data presented as mean ± SD. Significant pairwise comparisons are indicated on the graphs. $*p < 0.05$, $**p < 0.01$. Source data for this figure is available online.

Model	R6/2 (12 weeks)	zQ175DN (9 months)
Motor behavior and life span	Grip Strength - Significant improvement<br>Rotarod - Significant improvement<br>Open field - Significant improvement<br>Limb clasping - Significant improvement<br>Life span - Significant increase in females	**No motor phenotype** was observed until 12 months of age [19]. Heterozygous zQ175DN mice **do not display reduced survival** [28]
Brain atrophy	Forebrain area - Significant improvement<br>Whole brain volume - Non-significant improvement<br>Ventricular volume - Significant improvement	Forebrain area - **No phenotype**<br>Whole brain volume - **No phenotype**<br>Ventricular volume - **No phenotype**
Neuroinflammation	Astrogliosis - Significant improvement<br>Microgliosis - Non-significant improvement	Astrogliosis - **No phenotype**<br>Microgliosis - **No phenotype**
mHTT aggregate load	Nuclear inclusions - Significant improvement<br>SDS-insoluble inclusions - Significant improvement	Nuclear inclusions - Non-significant improvement<br>SDS-insoluble inclusions - Significant improvement
Striatal markers	DARPP-32 - Significant improvement<br>PDE10A - Significant improvement	DARPP-32 - **No phenotype**<br>PDE10A - Improvement
Synapse density	Dendritic spine density - Significant improvement<br>Excitatory synapses - Non-significant improvement	Excitatory synapses - **No phenotype**

Appendix Table S1. Summary of the effects of anle138b on HD-related phenotypes in the R6/2 and zQ175DN mouse models.
