# Supplementary material for: Anle138b ameliorates pathological phenotypes in mouse and cellular models of Huntington’s disease
Source: EMBO Mol Med. 2026 Jun 26;18(7):2838–66. doi: 10.1038/s44321-026-00459-9 (PMC13365221; doi:10.1038/s44321-026-00459-9)
Supplement: Supplementary file 20 — Expanded View Figures [file 44321_2026_459_MOESM20_ESM.pdf]

Expanded View Figures

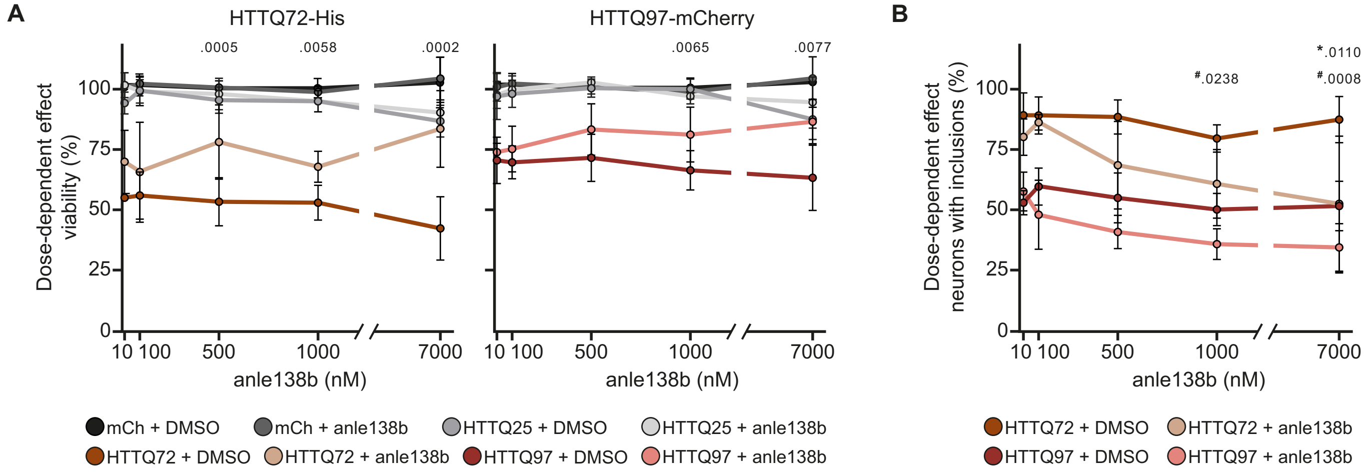

Figure EV1. Dose-dependent effect of anle138b in primary neurons.

(**A**) Dose-dependent effect of anle138b on the percentage of viable transfected neurons expressing HTTQ72-His (left) or HTTQ97-mCherry (right). Repeated measures ANOVA with Tukey's multiple comparison test. (**B**) Dose-dependent effect of anle138b on the fraction of neurons with mHTT inclusion bodies. Repeated measures ANOVA with Tukey's multiple comparison test. Data presented as mean ± SD. *p* values for significant pairwise comparisons are indicated on the graphs: # Difference between the "HTTQ72-His" conditions; * difference between the "HTTQ97-mCherry" conditions. $n = 4$ independent experiments. Source data are available online for this figure

© The Author(s)

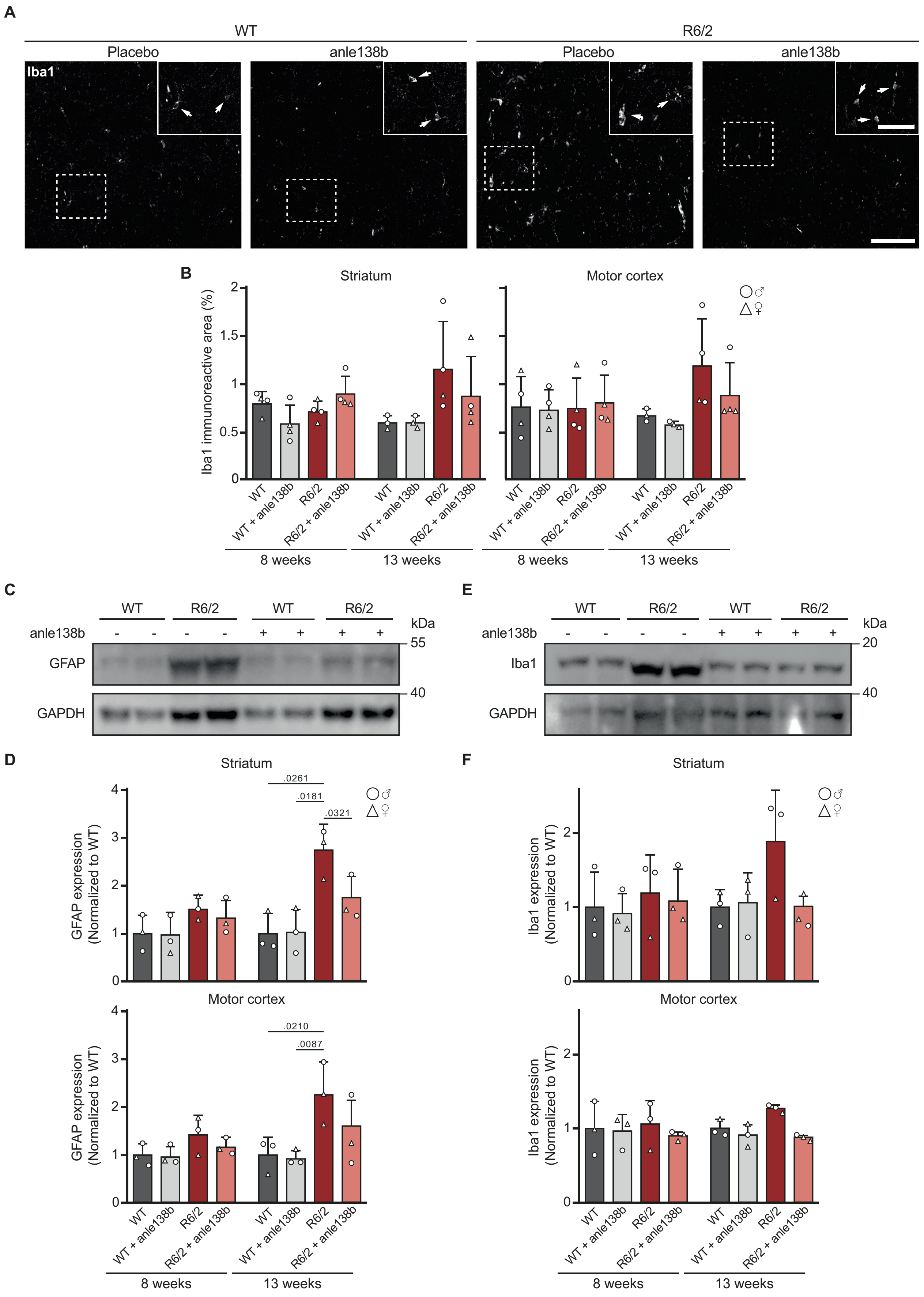
A
WT
R6/2
Placebo
anle138b
Placebo
anle138b
Iba1
B
Striatum
Motor cortex
Iba1 immunoreactive area (%)
WT
WT + anle138b
R6/2
R6/2 + anle138b
8 weeks
13 weeks
C
WT
R6/2
WT
R6/2
kDa
anle138b
GFAP
GAPDH
55
40
E
WT
R6/2
WT
R6/2
kDa
anle138b
Iba1
GAPDH
20
40
D
Striatum
GFAP expression (Normalized to WT)
.0261
.0181
.0321
Motor cortex
.0210
.0087
F
Striatum
Iba1 expression (Normalized to WT)
Motor cortex
8 weeks
13 weeks

Figure EV2. Microgliosis and astrogliosis in anle138b-treated R6/2 mice.

(**A**) Representative images of Iba1 immunostaining in the dorsal striatum of 13-week-old WT and R6/2 mice treated with placebo or anle138b. Insets show higher magnification of the areas delineated by the dashed boxes. White arrows point to examples of microglia. (**B**) Fraction of Iba1 immunopositive area in the striatum and motor cortex of 8 and 13-week-old WT and R6/2 mice. Two-way ANOVA with Bonferroni's multiple comparison test, not significant. $n = 4$ (8 weeks) or 3–4 mice (13 weeks) per group. (**C**) Representative immunoblot for GFAP in striatal lysates of 13-week-old WT and R6/2 mice treated with placebo or anle138b. GAPDH was used as a loading control. (**D**) Quantification of GFAP expression levels in the striatum and motor cortex of 8- and 13-week-old WT and R6/2 mice. Values were normalized to WT/placebo. Two-way ANOVA with Bonferroni's multiple comparison test, per age group and brain region. Striatum, 8 weeks: not significant; 13 weeks: Treatment, $^*p = 0.0391$; Genotype, $^{**}p = 0.0064$; Treatment × Genotype, $^*p = 0.0291$. Motor cortex, 8 weeks: not significant; 13 weeks: Treatment, $^*p = 0.0478$; Genotype, $^*p = 0.0140$; Treatment × Genotype, $p = 0.0687$. $n = 3$ mice per group. (**E**) Representative immunoblot for Iba1 in striatal lysates of 13-week-old WT and R6/2 mice treated with placebo or anle138b. GAPDH was used as a loading control. (**F**) Quantification of Iba1 expression levels in the striatum and motor cortex of 8- and 13-week-old WT and R6/2 mice. Values were normalized to WT/placebo. Two-way ANOVA with Bonferroni's multiple comparison test, per age group and brain region, not significant. $n = 3$ mice per group. Data presented as mean ± SD. p values for significant pairwise comparisons are indicated on the graphs. Scale bars in (**B**) 100 µm; insets, 50 µm. Source data are available online for this figure

 © The Author(s)

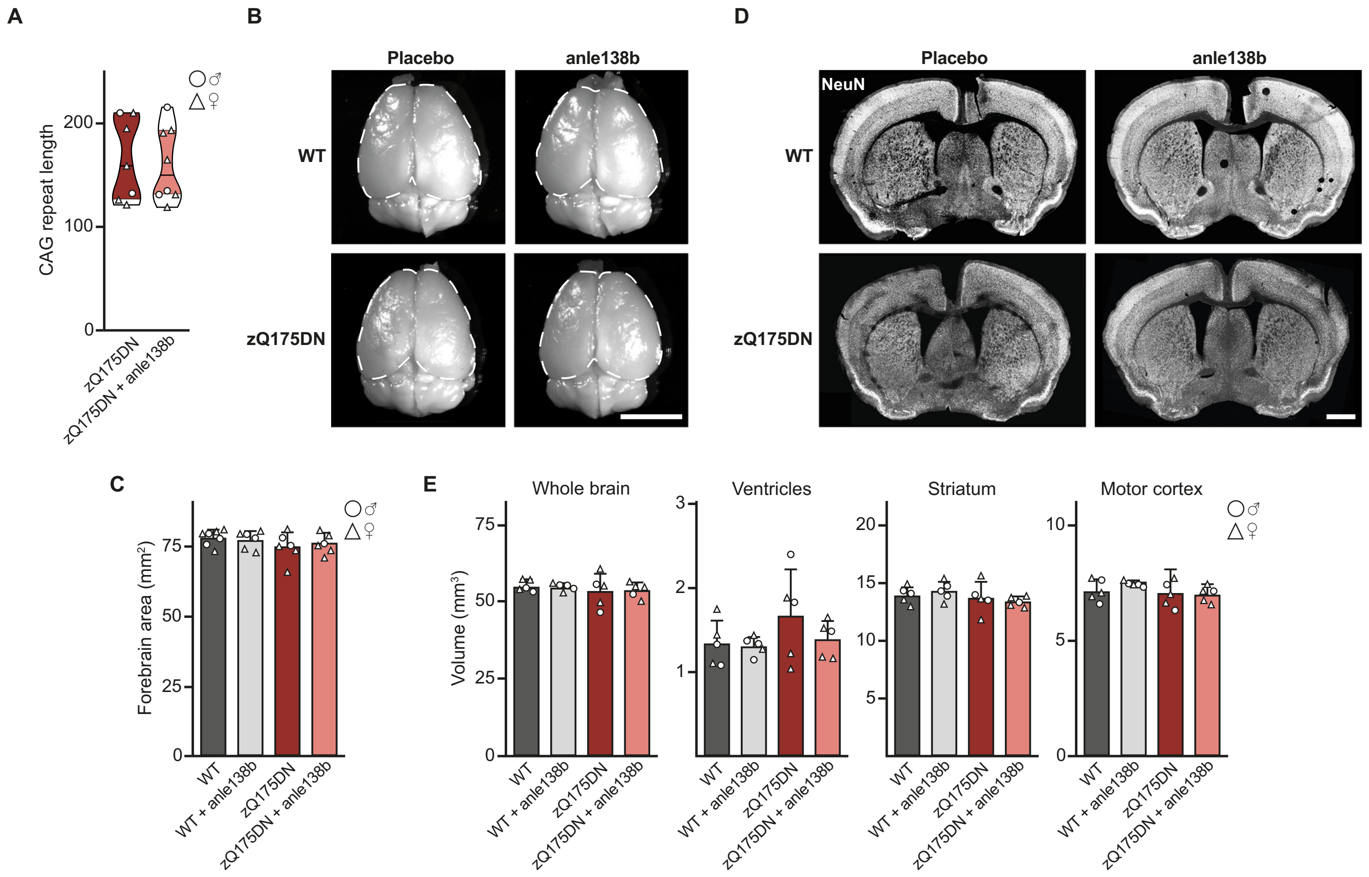

Figure EV3. Unchanged brain morphology in zQ175DN mice.

(**A**) CAG repeat length in 9-month-old zQ175DN mice. Unpaired two-tailed *t*-test, not significant. $n = 7$–8 mice per group. (**B**) Representative images of the brains of 9-month-old WT and zQ175DN mice treated with placebo or anle138b. Dashed white lines outline the forebrain. (**C**) Quantification of forebrain area in 9-month-old WT and zQ175DN mice. Two-way ANOVA with Bonferroni's multiple comparison test, not significant. $n = 6$–7 mice per group. (**D**) Representative coronal brain sections of 9-month-old WT and zQ175DN mice treated with placebo or anle138b, immunostained for the neuronal marker NeuN. (**E**) Whole brain, ventricle, striatum and motor cortex volume quantification in 9-month-old WT and zQ175DN mice. Two-way ANOVA with Bonferroni's multiple comparison test, not significant. $n = 5$ mice per group. Data presented as mean ± SD. Scale bars in (**B**) 5 mm; (**D**) 1 mm. Source data are available online for this figure

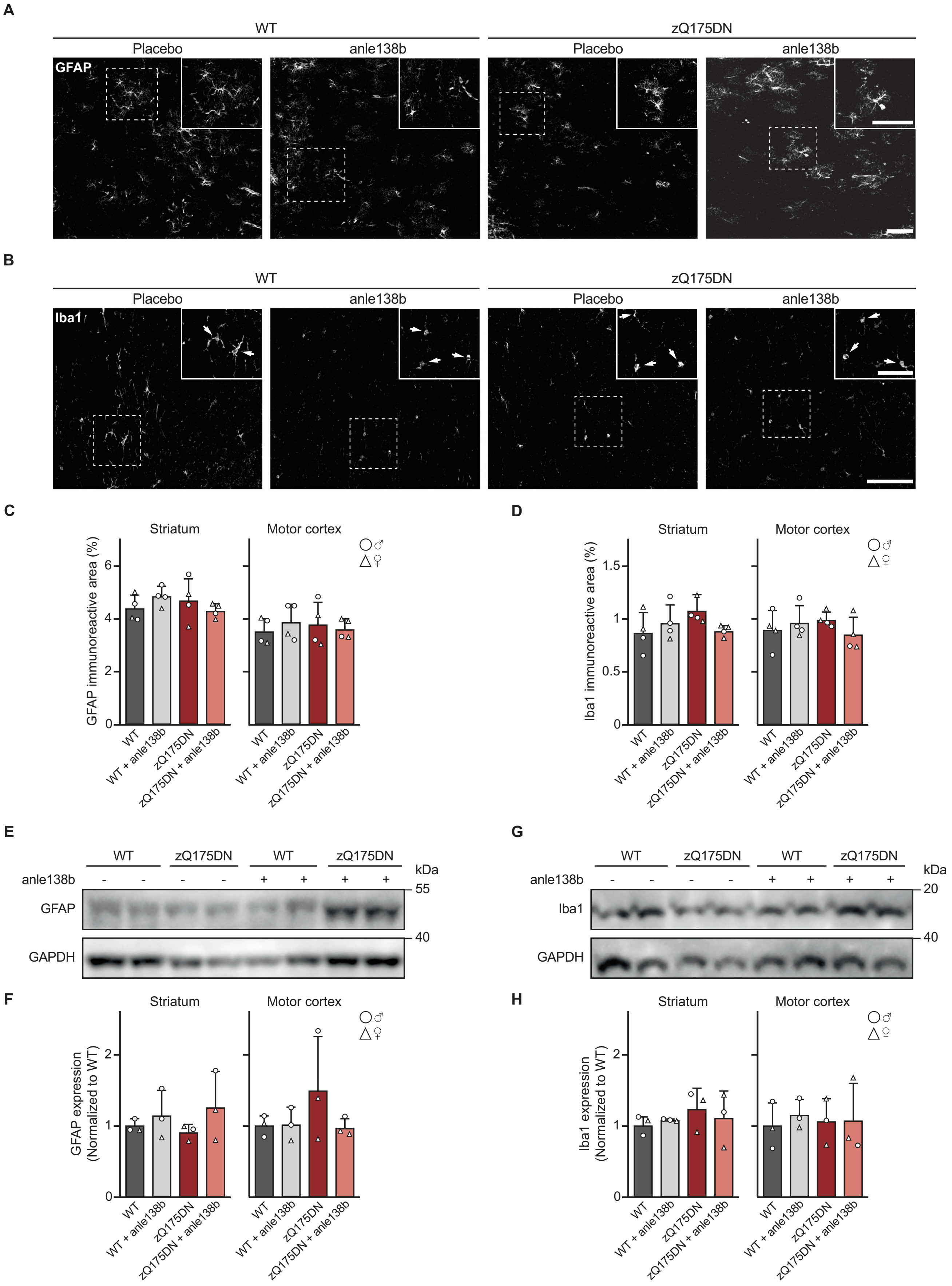
A
WT
zQ175DN
Placebo
anle138b
Placebo
anle138b
GFAP
B
WT
zQ175DN
Placebo
anle138b
Placebo
anle138b
Iba1
C
Striatum
Motor cortex
GFAP immunoreactive area (%)
WT
WT + anle138b
zQ175DN
zQ175DN + anle138b
D
Striatum
Motor cortex
Iba1 immunoreactive area (%)
E
WT
zQ175DN
WT
zQ175DN
anle138b
kDa
55
40
GFAP
GAPDH
G
WT
zQ175DN
WT
zQ175DN
anle138b
kDa
20
40
Iba1
GAPDH
F
Striatum
Motor cortex
GFAP expression (Normalized to WT)
H
Striatum
Motor cortex
Iba1 expression (Normalized to WT)

 © The Author(s)

Figure EV4. zQ175DN mice do not exhibit signs of neuroinflammation.

(**A**) Representative images of GFAP immunostaining in the dorsal striatum of 9-month-old WT and zQ175DN mice treated with placebo or anle138b. Insets show magnification of the areas delineated by the white dashed boxes. (**B**) Representative images of Iba1 immunostaining in the dorsal striatum of 9-month-old WT and zQ175DN mice treated with placebo or anle138b. Insets show magnification of the areas delineated by the white dashed boxes. Arrows point to examples of microglia. (**C**) Fraction of GFAP immunopositive area in the striatum and motor cortex of 9-month-old WT and zQ175DN mice. Two-way ANOVA with Bonferroni's multiple comparison test, not significant. $n = 4$ mice per group. (**D**) Fraction of Iba1 immunopositive area in the striatum and motor cortex of 9-month-old WT and zQ175DN mice. Two-way ANOVA with Bonferroni's multiple comparison test, not significant. $n = 4$ mice per group. (**E**) Representative immunoblot for GFAP in striatal lysates of 9-month-old WT and zQ175DN mice treated with placebo or anle138b. GAPDH was used as a loading control. (**F**) Quantification of GFAP expression levels in the striatum and motor cortex of 9-month-old WT and zQ175DN mice. Values were normalized to WT/placebo. Two-way ANOVA with Bonferroni's multiple comparison test, not significant. $n = 3$ mice per group. (**G**) Representative immunoblot for Iba1 in striatal lysates of 9-month-old WT and zQ175DN mice treated with placebo or anle138b. GAPDH was used as a loading control. (**H**) Quantification of Iba1 expression levels in the striatum and motor cortex of 9-month-old WT and zQ175DN mice. Values were normalized to WT/placebo. Two-way ANOVA with Bonferroni's multiple comparison test, not significant. $n = 3$ mice per group. Data presented as mean ± SD. Scale bars in (**A**, **B**) 100 µm; insets, 50 µm. Source data are available online for this figure

© The Author(s)

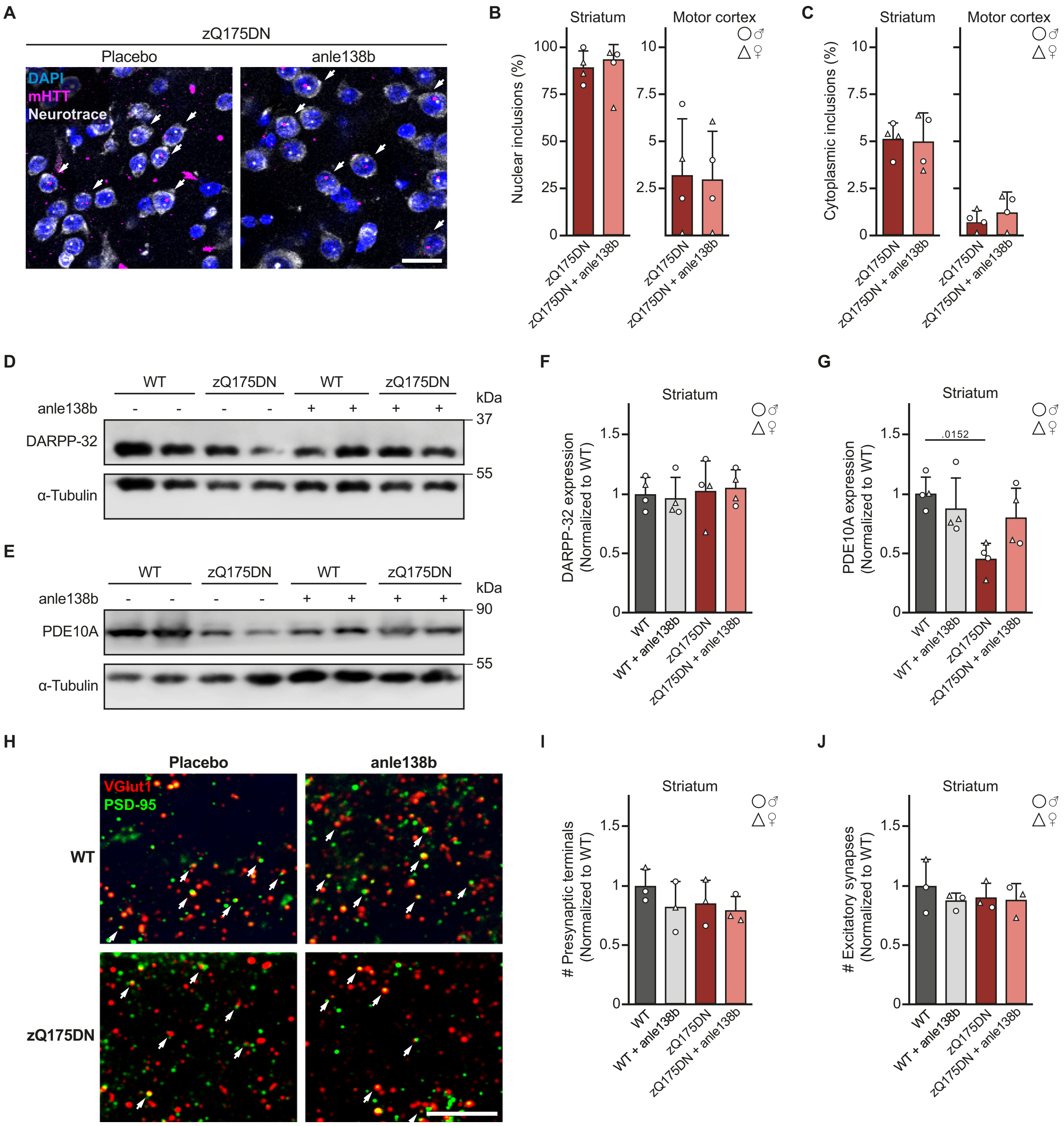
A
zQ175DN
Placebo
anle138b
DAPI
mHTT
Neurotrace
B
Striatum
Motor cortex
Nuclear inclusions (%)
zQ175DN
zQ175DN + anle138b
C
Cytoplasmic inclusions (%)
D
WT
zQ175DN
anle138b
DARPP-32
α-Tubulin
kDa
37
55
E
PDE10A
90
F
DARPP-32 expression (Normalized to WT)
WT + anle138b
G
PDE10A expression (Normalized to WT)
.0152
H
VGlut1
PSD-95
I
Presynaptic terminals (Normalized to WT)
J
Excitatory synapses (Normalized to WT)

 © The Author(s)

Figure EV5. Effects of anle138b on inclusion load, striatal markers and synapse density in zQ175DN mice.

(**A**) Representative images of neurons with nuclear mHTT inclusions (magenta) in the motor cortex of 9-month-old zQ175DN mice treated with placebo or anle138b. Neurons were identified by Neurotrace labeling, and aggregated mHTT was detected by EM48 immunostaining. Nuclei were labeled with DAPI. White arrows point to neurons with mHTT inclusion bodies. (**B**) Quantification of the fraction of neurons with nuclear mHTT inclusion bodies in the striatum and motor cortex of 9-month-old zQ175DN mice. Unpaired two-tailed *t*-test, not significant. $n = 4$ mice per group. (**C**) Quantification of the fraction of neurons with cytoplasmic mHTT inclusion bodies in the striatum and motor cortex of 9-month-old zQ175DN mice. Unpaired two-tailed *t*-test, not significant. $n = 4$ mice per group. (**D**) Representative immunoblot for DARPP-32 in striatal lysates of 9-month-old WT and zQ175DN mice treated with placebo or anle138b. α-Tubulin was used as a loading control. (**E**) Representative immunoblot for PDE10A in striatal lysates of 9-month-old WT and zQ175DN mice treated with placebo or anle138b. α-Tubulin was used as a loading control. (**F**) Quantification of DARPP-32 expression levels. Values were normalized to WT/placebo. Two-way ANOVA with Bonferroni's multiple comparison test, not significant. $n = 4$ mice per group. (**G**) Quantification of PDE10A expression levels. Values were normalized to WT/placebo. Two-way ANOVA with Bonferroni's multiple comparison test. Treatment, $p = 0.3005$; Genotype, $^{*}p = 0.01$; Treatment × Genotype, $^{*}p = 0.0390$. $n = 4$ mice per group. (**H**) Representative images of immunostained VGlut1 and PSD-95 puncta in the dorsal striatum of 9-month-old WT and zQ175DN mice treated with placebo or anle138b. Excitatory synapses were identified by the overlap between the puncta in the two channels (examples indicated by white arrows). (**I**) Quantification of the number of VGlut1 puncta in the striatum of 9-month-old WT and zQ175DN mice. Two-way ANOVA with Bonferroni's multiple comparison test, not significant. $n = 3$ mice per group. (**J**) Quantification of the number of overlapping VGlut1/PSD-95 puncta in the striatum of 9-month-old WT and zQ175DN mice. Two-way ANOVA with Bonferroni's multiple comparison test, not significant. $n = 3$ mice per group. Data presented as mean ± SD. *p* values for significant pairwise comparisons are indicated on the graphs. Scale bars: (**A**) 20 µm; (**H**) 5 µm. Source data are available online for this figure

© The Author(s)
