# Supplementary material for: Anle138b ameliorates pathological phenotypes in mouse and cellular models of Huntington’s disease
Source: EMBO Mol Med. 2026 Jun 26;18(7):2838–66. doi: 10.1038/s44321-026-00459-9 (PMC13365221; doi:10.1038/s44321-026-00459-9)
Supplement: Supplementary file 2 — Peer Review File [file 44321_2026_459_MOESM2_ESM.pdf]

EMBO Molecular Medicine

Anle138b ameliorates pathological phenotypes in mouse and cellular models of Huntington's disease

Miguel da Silva Padilha, Seda Koyuncu, Evangeline Chabanis, Sergey Ryazanov, Andrei Leonov, David Vilchez, Rüdiger Klein, Armin Giese, Christian Griesinger, and Irina Dudanova

Corresponding author(s): Irina Dudanova (irina.dudanova@uni-wuerzburg.de)

Review Timeline:

Submission Date:	5th Mar 25
Editorial Decision:	16th Apr 25
Revision Received:	27th Feb 26
Editorial Decision:	4th May 26
Revision Received:	18th May 26
Accepted:	20th May 26

Editor: Jingyi Hou

Transaction Report:

(Note: With the exception of the correction of typographical or spelling errors that could be a source of ambiguity, letters and reports are not edited. Depending on transfer agreements, referee reports obtained elsewhere may or may not be included in this compilation. Referee reports are anonymous unless the Referee chooses to sign their reports.)

1st Editorial Decision 16th Apr 2025

17th Apr 2025

Dear Prof. Dudanova,

Thank you again for submitting your work to EMBO Molecular Medicine. We have now received feedback from the three referees who agreed to evaluate your manuscript. As you will see in the reports below, the referees find your study of interest but have raised several concerns that will need to be thoroughly addressed in a major revision of the manuscript.

The referees' recommendations are clear, so I won't repeat the points listed below. It is important to carefully address all the issues raised by the referees. Please feel free to contact me in case you would like to discuss in further detail any of the issues raised by the reviewers.

We would welcome the submission of a revised version within six months for further consideration. As you may already know, our editorial policy allows in principle a single round of major revision, and it is therefore essential to provide responses to the referees' comments that are as complete as possible.

EMBO Molecular Medicine has a "scooping protection" policy, whereby similar findings that are published by others during review or revision are not a criterion for rejection. Should you decide to submit a revised version, I do ask that you get in touch after six months if you have not completed it, to update us on the status.

Please also contact us as soon as possible if similar work is published elsewhere. If other work is published, we may not be able to extend the revision period beyond six months.

Please read below for important editorial formatting and consult our author's guidelines for proper formatting of your revised article for EMBO Molecular Medicine.

I look forward to receiving your revised manuscript.

Yours sincerely,
Jingyi

Jingyi Hou
Senior Editor
EMBO Molecular Medicine

*****

When submitting your revised manuscript, please carefully review the instructions that follow below. We perform an initial quality control of all revised manuscripts before re-review; failure to include requested items will delay the evaluation of your revision.

We require:

1) A .docx formatted version of the manuscript text (including legends for main figures, EV figures and tables). Please make sure that the changes are highlighted to be clearly visible.

2) Individual production quality figure files as .eps, .tif, .jpg (one file per figure). For guidance, download the 'Figure Guide PDF': (https://www.embopress.org/page/journal/17574684/authorguide#figureformat).

3) A .docx formatted letter INCLUDING the reviewers' reports and your detailed point-by-point responses to their comments. As part of the EMBO Press transparent editorial process, the point-by-point response is part of the Review Process File (RPF), which will be published alongside your paper.

4) A complete author checklist, which you can download from our author guidelines (https://www.embopress.org/page/journal/17574684/authorguide#submissionofrevisions). Please insert information in the checklist that is also reflected in the manuscript. The completed author checklist will also be part of the RPF.

5) Please note that all corresponding authors are required to supply an ORCID ID for their name upon submission of a revised

manuscript.

6) It is mandatory to include a 'Data Availability' section after the Materials and Methods. Before submitting your revision, primary datasets produced in this study need to be deposited in an appropriate public database, and the accession numbers and database listed under 'Data Availability'. Please remember to provide a reviewer password if the datasets are not yet public (see https://www.embopress.org/page/journal/17574684/authorguide#dataavailability).

In case you have no data that requires deposition in a public database, please state so in this section. Note that the Data Availability Section is restricted to new primary data that are part of this study.

7) For data quantification: please specify the name of the statistical test used to generate error bars and P values, the number (n) of independent experiments (specify technical or biological replicates) underlying each data point and the test used to calculate p-values in each figure legend. The figure legends should contain a basic description of n, P and the test applied. Graphs must include a description of the bars and the error bars (s.d., s.e.m.). See also 'Figure Legend' guidelines: https://www.embopress.org/page/journal/17574684/authorguide#figureformat

8) At EMBO Press we ask authors to provide source data for the main manuscript figures. You will receive a separate email with instructions for providing source data with your revised manuscript, including how to upload and organize the files.

9) Our journal encourages inclusion of *data citations in the reference list* to directly cite datasets that were re-used and obtained from public databases. Data citations in the article text are distinct from normal bibliographical citations and should directly link to the database records from which the data can be accessed. In the main text, data citations are formatted as follows: "Data ref: Smith et al, 2001" or "Data ref: NCBI Sequence Read Archive PRJNA342805, 2017". In the Reference list, data citations must be labeled with "[DATASET]". A data reference must provide the database name, accession number/identifiers and a resolvable link to the landing page from which the data can be accessed at the end of the reference. Further instructions are available at .

10) We replaced Supplementary Information with Expanded View (EV) Figures and Tables that are collapsible/expandable online. A maximum of 5 EV Figures can be typeset. EV Figures should be cited as 'Figure EV1, Figure EV2" etc... in the text and their respective legends should be included in the main text after the legends of regular figures.

- For the figures that you do NOT wish to display as Expanded View figures, they should be bundled together with their legends in a single PDF file called *Appendix*, which should start with a short Table of Content. Appendix figures should be referred to in the main text as: "Appendix Figure S1, Appendix Figure S2" etc.

- Additional Tables/Datasets should be labeled and referred to as Table EV1, Dataset EV1, etc. Legends have to be provided in a separate tab in case of .xls files. Alternatively, the legend can be supplied as a separate text file (README) and zipped together with the Table/Dataset file.

See detailed instructions here:

.

11) The paper explained: EMBO Molecular Medicine articles are accompanied by a summary of the articles to emphasize the major findings in the paper and their medical implications for the non-specialist reader. Please provide a draft summary of your article highlighting

- the medical issue you are addressing,

- the results obtained and

- their clinical impact.

This may be edited to ensure that readers understand the significance and context of the research. Please refer to any of our published articles for an example.

12) Author contributions: You will be asked to provide CRediT (Contributor Role Taxonomy) terms in the submission system. These replace a narrative author contribution section in the manuscript.

13) A Conflict of Interest statement should be provided in the main text.

14) Every published paper now includes a 'Synopsis' to further enhance discoverability. Synopses are displayed on the journal webpage and are freely accessible to all readers. They include a short stand first (maximum of 300 characters, including space)

as well as 2-5 one-sentences bullet points that summarizes the paper. Please write the bullet points to summarize the key NEW findings. They should be designed to be complementary to the abstract - i.e. not repeat the same text. We encourage inclusion of key acronyms and quantitative information (maximum of 30 words / bullet point). Please use the passive voice. Please attach these in a separate file or send them by email, we will incorporate them accordingly.

Please also suggest a visual abstract to illustrate your article as a PNG file 550 px wide x 300-600 px high.

15) All Materials and Methods need to be described in the main text using our 'Structured Methods' format. According to this format, the Methods section includes a Reagents and Tools Table (listing key reagents, experimental models, software and relevant equipment and including their sources and relevant identifiers) followed by a Methods and Protocols section describing the methods, ideally using a step-by-step protocol format. The aim is to facilitate adoption of the methodologies across labs.

Please download and fill our Reagents and Tools Table template (.docx), which you can find in our author guidelines: https://www.embopress.org/page/journal/17574684/authorguide#structuredmethods

When submitting your revised manuscript, please DO NOT include the Reagents and Tools Table in the Methods section of the manuscript but upload it as a separate file choosing the file type "Reagent Table".

EMBO Molecular Medicine has a "scooping protection" policy, whereby similar findings that are published by others during review or revision are not a criterion for rejection. Should you decide to submit a revised version, I do ask that you get in touch after three months if you have not completed it, to update us on the status.

***** Reviewer's comments *****

Referee #1 (Remarks for Author):

In this work da Silva Padilha et al. suggest the use of the compound anle138b for therapeutic intervention against Huntington's disease (HD). Given the current lack of therapies for HD, the possibility to apply this approach is relevant, also because apparently the compound can be orally administered and it seems to be able to cross the blood-brain-barrier. A phase 2 trial for multiple system atrophy is ongoing, further suggesting translational potential for this compound. The manuscript is well written and the topic is relevant and interesting also for the non-specialist.
However, there are a few concerns about the medical impact of the results based on the experiments and analyses presented here. Although the Authors include different cellular and mouse models, to show the successful application of anle138b, more analyses should be included to strengthen the conclusions before publication.
Major criticisms
1) The rescue experiments in the R6/2 mice show limited effects on multiple parameters; although these are statistically significant, there is a minimal effect on mouse survival and motor amelioration, although the drug is administered already during conception. The Authors include another model in which they could show some phenotypic benefits when starting the treatment in adult mice. This additional model is a major strength of this study. However, the zQ175 heterozygous mice provide a limited set of readouts for showing beneficial effects by anle138b, with the exception of a few markers (PDE10A as a striatal marker only) and the effect of mutant Huntingtin aggregates. In my view, the analysis at the transcriptomic level of molecular signatures typical of HD and the effects of anle138b on these, in at least the mouse striatum, would help to better understand whether molecular changes and cellular processes will be affected by anle138b and to what extent these are rescued by the treatment. This analysis would be important because it is unclear how anle138b functions, and whether the effect on mutant Huntingtin accumulation is determinant to cure HD.
2) The role of anle13b on mHTT is unclear. How specific are the effects of anle138b for mHTT? Are the effects on mHTT inclusions in the nucleus or also cytoplasmic? And how to explain a reduction only at 8 weeks but not later in the striatum? Why are the effects on mHTT in both ages in the cortex? In the methods, it seems that for the mouse experiments, only inclusions overlapping with the nuclear DAPI signal are measured. Given the controversial impact of mHTT in the nuclei and cytoplasm, it would be important to differentiate these effects and to provide an explanation to the questions above.
Minor points
1) How is explained the insoluble mHTT detection in the wt for the filter retardation assay?
2) For the Western blots in Fig 5, 3 mice per group are used. How many technical replicates were performed?
3) Only one cell line is provided for the human HD cellular model, that was not differentiated in a striatal cell line: it would be important to include at least another cell line and/or differentiated neurons to account for the interindividual variability, otherwise the rationale for including this model is unclear.
4) The methods should include information about the image quantification in terms of number of images, considered brain region coordinates, how many cells, sections per mouse.
5) CAG repeat length in the zQ175 mice is missing and should be provided.

Referee #2 (Comments on Novelty/Model System for Author):

While the study is well-structured and presents compelling data-particularly in the R6/2 transgenic model, which over-expresses a truncated human huntingtin fragment with an expanded CAG repeat-the findings are only modestly validated in the zQ175 knock-in model. The latter is widely recognized as a physiologically relevant mouse model for Huntington's disease (HD), as it more closely recapitulates the full-length endogenous mutation seen in patients. This discrepancy somewhat limits the translational strength of the conclusions. The therapeutic potential of anle138b is of significant interest, especially given the current lack of effective treatments that can modify disease progression or delay onset in HD. However, a clearer link between the reduction of aggregates and key functional outcomes-such as somatic instability, transcriptional dysregulation, and neuronal loss-is needed to better define the therapeutic relevance of anle138b.
Specifically, several critical limitations temper the overall enthusiasm for the findings:
Lack of Assessment of Somatic Instability:
Emerging research highlights somatic CAG repeat instability-particularly in medium spiny neurons of the striatum-as a key determinant of neuronal vulnerability in HD. Given that aggregate toxicity and oxidative stress have been linked to increased somatic instability and neuronal loss, a relevant point that was not considered by the authors is whether anle138b modulates somatic instability. Integrating this analysis would provide valuable insight into the compound's potential disease-modifying effects.
Need for Direct Comparative Analysis Between Models:
The use of both the R6/2 and zQ175 models is a strength of the study; however, a more structured, side-by-side comparison of phenotypic and molecular responses between the two models would greatly enhance the interpretability and translational relevance of the results. Highlighting both convergent and divergent outcomes could yield deeper insights into the compound's mode of action across disease stages and genetic contexts.
High Phenotypic Variability in some phenotypes:
Several phenotypic readouts in the R6/2 mice (Fig. 4) and zQ175 (Fig. 7) exhibit substantial inter-animal variability, which raises concerns about reproducibility and statistical robustness. Increasing the sample size in these cohorts would help to better interpret the observed trends.
Transcriptomic Rescue in R6/2 and zQ175 Not Explored:
Previous studies have demonstrated widespread transcriptional dysregulation in R6/2 and zQ175 mice, particularly around 4-6 months of age. Investigating whether anle138b can reverse or mitigate these genome-wide molecular alterations would significantly strengthen the claim of its disease-modifying potential and provide important insight into its mechanism of action.

Referee #2 (Remarks for Author):

Huntington's disease (HD) is a fatal neurodegenerative disorder caused by a CAG repeat expansion in the HTT gene, leading to toxic protein aggregation and brain atrophy. The study by Miguel da Silva Padilha et al., testes the effects of the oligomer-modifying compound anle138b (emrusolmin) to reduce mutant huntingtin (mHTT) aggregation and neurotoxicity in cell cultures, HD mouse models, and patient-derived neural precursor cells.
While the study is well-structured and presents compelling data-particularly in the R6/2 transgenic model, which over-expresses a truncated human huntingtin fragment with an expanded CAG repeat-the findings are only modestly validated in the zQ175 knock-in model. The latter is widely recognized as a physiologically relevant mouse model for Huntington's disease (HD), as it more closely recapitulates the full-length endogenous mutation seen in patients. This discrepancy somewhat limits the translational strength of the conclusions. The therapeutic potential of anle138b is of significant interest, especially given the current lack of effective treatments that can modify disease progression or delay onset in HD. However, a clearer link between the reduction of aggregates and key functional outcomes-such as somatic instability, transcriptional dysregulation, and neuronal loss-is needed to better define the therapeutic relevance of anle138b.
Specifically, several critical limitations temper the overall enthusiasm for the findings:
Lack of Assessment of Somatic Instability:
Emerging research highlights somatic CAG repeat instability-particularly in medium spiny neurons of the striatum-as a key determinant of neuronal vulnerability in HD. Given that aggregate toxicity and oxidative stress have been linked to increased somatic instability and neuronal loss, a relevant point that was not considered by the authors is whether anle138b modulates somatic instability. Integrating this analysis would provide valuable insight into the compound's potential disease-modifying effects.
Need for Direct Comparative Analysis Between Models:
The use of both the R6/2 and zQ175 models is a strength of the study; however, a more structured, side-by-side comparison of phenotypic and molecular responses between the two models would greatly enhance the interpretability and translational relevance of the results. Highlighting both convergent and divergent outcomes could yield deeper insights into the compound's mode of action across disease stages and genetic contexts.
High Phenotypic Variability in some phenotypes:
Several phenotypic readouts in the R6/2 mice (Fig. 4) and zQ175 (Fig. 7) exhibit substantial inter-animal variability, which raises concerns about reproducibility and statistical robustness. Increasing the sample size in these cohorts would help to better interpret the observed trends.
Transcriptomic Rescue in R6/2 and zQ175 Not Explored:

Previous studies have demonstrated widespread transcriptional dysregulation in R6/2 and zQ175 mice, particularly around 4-6 months of age. Investigating whether anle138b can reverse or mitigate these genome-wide molecular alterations would significantly strengthen the claim of its disease-modifying potential and provide important insight into its mechanism of action.
Additional cellular and molecular phenotypes to be explored in human iPSCs:
To strengthen the conclusion regarding the beneficial effects of anle138b in rescuing HD-relevant functional phenotypes, the phenotypic analysis in iPSC-derived neuronal progenitors could be expanded to include additional cellular and molecular readouts. These may include BDNF trafficking, cell adhesion properties, and transcriptomic alterations, given their established relevance in HD pathology.

Minor points:

1. While the volumetric analyses of the striatum, cortex, and ventricles are relevant and encouraging, the study would benefit from incorporating more refined and sensitive methodologies, such as high-resolution structural MRI. This would allow for a more quantitative and nuanced assessment of brain atrophy, enabling the detection of subtle but biologically meaningful changes that may not be apparent with standard histological techniques.

2. The analysis of astrogliosis and microglia activation in the mouse brain parenchyma could benefit from an integrative quantitative analysis through western blot experiments, similar to what the authors performed for DARPP32

3. Considering the current focus on DNA repair, somatic instability of the expanded CAG tract as major contributors to the HD pathology, a discussion integrating these points with the relevance of treating aggregation in HD would be recommended.

Referee #3 (Comments on Novelty/Model System for Author):

This study uses 4 different models: primary mouse neurons, 2 different mice models (R6/2 and zQ175DN knock-in HD mice) and iPSC derived NPC from 1 HD patient. The rational for the specific choice of models remains vague.

Referee #3 (Remarks for Author):

The paper reports that the oligomer-modifying molecule anle138b improves multiple disease phenotypes in cell culture and two HD mouse models. Anle138b reduces mHTT aggregate formation and neurotoxicity in primary neurons. In R6/2 mice, oral administration improves motor performance and survival in females, decreases GFAP-positive cells and mHTT aggregates in the striatum and motor cortex, and enhances striatal markers. At 13 weeks, spine density and VGlut1/PSD-95 puncta increase following treatment.

mHTT reduction effects were also observed in zQ175DN mice and one human iPSC derived NPC line. While anle138b shows potential for disease modification, the results remain preliminary. The use of four models with different analytical parameters raises concerns about selective data presentation. Models should be clearly indicated when multiple are used in a figure.

R6/2 data suggest anle138b's positive effects are mostly in female mice and only at specific (late) ages. The current interpretation overstates the findings and should be more cautious. Further analysis in older zQ175DN knock-in mice is needed.

The HD patient-derived iPSC model could help assess anle138b's impact in human disease, but the current data are very preliminary. Analysis should be conducted in differentiated, disease-relevant neurons from a sufficient number of HD patients and controls, including detailed morphological assessments (neurite integrity, proteostasis, cell death, transcriptome, and proteome). Clinically relevant mHTT measures should be included to estimate translational potential.

Minor issues:
Why are different magnifications used in panels A and D? Standardizing them would improve consistency.

Point-by-point response to reviewers' comments

Referee #1 (Remarks for Author):

In this work da Silva Padilha et al. suggest the use of the compound anle138b for therapeutic intervention against Huntington's disease (HD). Given the current lack of therapies for HD, the possibility to apply this approach is relevant, also because apparently the compound can be orally administered and it seems to be able to cross the blood-brain-barrier. A phase 2 trial for multiple system atrophy is ongoing, further suggesting translational potential for this compound. The manuscript is well written and the topic is relevant and interesting also for the non-specialist.

We thank the reviewer for acknowledging the strengths of our study.

However, there are a few concerns about the medical impact of the results based on the experiments and analyses presented here. Although the Authors include different cellular and mouse models, to show the successful application of anle138b, more analyses should be included to strengthen the conclusions before publication.

Major criticisms

1) The rescue experiments in the R6/2 mice show limited effects on multiple parameters; although these are statistically significant, there is a minimal effect on mouse survival and motor amelioration, although the drug is administered already during conception. The Authors include another model in which they could show some phenotypic benefits when starting the treatment in adult mice. This additional model is a major strength of this study. However, the zQ175 heterozygous mice provide a limited set of readouts for showing beneficial effects by anle138b, with the exception of a few markers (PDE10A as a striatal marker only) and the effect of mutant Huntingtin aggregates. In my view, the analysis at the transcriptomic level of molecular signatures typical of HD and the effects of anle138b on these, in at least the mouse striatum, would help to better understand whether molecular changes and cellular processes will be affected by anle138b and to what extent these are rescued by the treatment. This analysis would be important because it is unclear how anle138b functions, and whether the effect on mutant Huntingtin accumulation is determinant to cure HD.

We note that keeping in mind the short life span of the R6/2 model, which amounts to only 15-20 weeks, an increase of 5 days is a considerable improvement. We have made a text change to acknowledge the mild effect of anle138b on survival. The text now reads:
"…Administration of anle138b <u>modestly</u> prolonged the life span of R6/2 mice by 5 days on average" (p. 6, line 145).

We thank the reviewer for proposing transcriptomics analyses to more robustly evaluate the impact on disease phenotypes in zQ175DN mice. To this end, we have treated a cohort of zQ157DN mice and wildtype littermate controls with anle138b or placebo starting at 4 months of age, and harvested striatal tissue at 6 months, a time point early in disease progression when transcriptional dysregulation has been reported in this model (Langfelder et al., 2016), and conducted RNA-sequencing. Transcriptional alterations were mild in the zQ175DN mice at this age, but we could observe beneficial changes in the transcriptional profile of anle138b-treated mice. The results are described in the text on p. 10-11, lines 263-283, and presented in Fig. 7D-G and Appendix Fig. S4. See also Reviewer #2, comment 4.

2) The role of anle138b on mHTT is unclear. How specific are the effects of anle138b for mHTT? Are the effects on mHTT inclusions in the nucleus or also cytoplasmic? And how to explain a reduction only at 8 weeks but not later in the striatum? Why are the effects on mHTT in both ages in the cortex? In the methods, it seems that for the mouse experiments, only inclusions overlapping with the nuclear DAPI signal are measured. Given the controversial impact of mHTT in the nuclei and cytoplasm, it would be important to differentiate these effects and to provide an explanation to the questions above.

Previous studies showed that anle138b also acts on other aggregating proteins, as explained in the text on p. 3, lines 63-67: "*Anle138b was shown to directly interact with the oligomeric species of disease-related aggregating proteins such as prion protein, α-synuclein, amyloid-beta (Aβ), tau and human islet amyloid polypeptide, inhibiting formation of aggregates and reducing their toxicity (Albariqi et al., 2024; Heras-Garvin et al., 2019; Martinez Hernandez et al., 2018; Wagner et al., 2015; Wagner et al., 2013; Wegrzynowicz et al., 2019).*"

Since we did not observe any significant effects in wildtype animals, anle138b does not seem to improve neuronal fitness in general, but rather act specifically in a disease context.

The reviewer correctly points out that there is increasing evidence of different properties and effects of mHTT in the nucleus vs. cytoplasm. Our findings in primary neurons transfected with a nuclear (HTTQ72-His) and cytoplasmic versions of mHTT (HTTQ97-mCherry, Fig. 1B) suggest that anle138b acts both on nuclear as well as cytoplasmic mHTT inclusion bodies. Therefore, to address this question *in vivo*, we have separately quantified mHTT inclusions inside and outside the nucleus in the motor cortex and striatum of 8- and 12-week-old R6/2 mice and 9-month-old zQ175DN mice. In R6/2 mice, nuclear inclusions were reduced by anle138b treatment in both regions at 8 weeks and in the motor cortex at 12 weeks (Fig. 4B). In contrast, extranuclear inclusions are rare (<5% of neurons) at 8 weeks of age. At an age when they become more frequent (13 weeks), we observe a significant reduction in extranuclear inclusions in anle138b-traeted mice in the motor cortex and a trend in the striatum (Supplementary Fig. S2B). Taken together, anle138b slows down the accumulation of both intranuclear and extranuclear mHTT inclusions in the R6/2 model. These new data are described in the text on p. 7, lines 188-195.

In the zQ175DN mice, we did not detect significant changes in the fractions of either nuclear or cytoplasmic inclusion bodies. These new data are presented in Fig. EV5B-C.

At the investigated timepoints, we did not observe an effect of anle138b on mHTT inclusions in the striatum of R6/2 mice. This is likely due to the severe nature of this model, which develops early and abundant pathology in the striatum. Please note that the fraction of neurons with inclusions at 13 weeks is almost 100%. We hypothesize that anle138b slows down the formation of inclusions, which eventually form in all cells, but at a later age. This is supported by a reduction in inclusion frequency at 8 weeks. However, the 13-week time point is likely already outside of the dynamic range of this parameter, with inclusion burden being saturated in both control and treated mice. In contrast, inclusion formation in the cortex is significantly slower (as we have reported previously, see (Hosp et al., 2017) and does not yet reach saturation at 13 weeks.

Minor points

3) How is explained the insoluble mHTT detection in the wt for the filter retardation assay?

It is common to observe some background signal in the filter trap assay as in Fig. 4C and 7B, especially when working with tissue lysates. All values are normalized to the signal observed in the wildtype (control) conditions, so that final results account for the existence of this background.

4) For the Western blots in Fig 5, 3 mice per group are used. How many technical replicates were performed?

One technical replicate was performed for each mouse. The data points on the graph represent individual animals, i.e., biological replicates.

5) Only one cell line is provided for the human HD cellular model, that was not differentiated in a striatal cell line: it would be important to include at least another cell line and/or differentiated neurons to account for the interindividual variability, otherwise the rationale for including this model is unclear.

As suggested by the reviewer, we have performed experiments in an additional HD-iPSC line, Q180 (HD iPSC Consortium, 2012). Importantly, we have also included an isogenic control available for this line. The new data, presented in Fig. 8D-E, is consistent with our previous findings in Q71 cells, now shown in Supplementary Fig. S4A-B. To complement the filter trap analysis with an orthogonal assay, we have quantified mHTT inclusions in Q180-NPCs by immunostaining. In line with the biochemical evidence, inclusions induced by MG-132 treatment of Q180 cells were significantly reduced by anle138b treatment. These results are described on p. 11, lines 287-297.

We did not differentiate iPSCs into striatal neurons for the following reasons: First, differentiation into striatal neurons has very low efficiency. In addition, HD-iPSC-derived neurons do not exhibit robust neurodegeneration-related phenotypes. In particular, they do not develop mHTT inclusions (HD iPSC Consortium, 2012; Noormohammadi et al., 2016). Thus, it is not possible to investigate the effect of anle138b in this system.

6) The methods should include information about the image quantification in terms of number of images, considered brain region coordinates, how many cells, sections per mouse.

We have included the respective information into the respective sections of the Materials and Methods:

p. 17, lines 466-467 (Immunocytochemistry on primary neurons):

„For all analyses, n = 4 – 6 independent experiments and 20 – 35 cells were considered per condition."

p. 18-19, lines 516-518, 521, 525-530, 534-535 (Immunocytochemistry on mouse brain sections):

„Reference anatomical landmarks such as the anterior commissure and the corpus callosum were used to choose the starting sections and verify the consistency of the final sections (Bregma = 1.045 mm to Bregma = - 0.755 mm). …
Immunoreactivity was analyzed in the striatum and motor cortex (Bregma = 0.345 ± 0.1 mm)…
For each mouse, two brain sections were analyzed. The percentage of inclusion bearing neurons was analyzed in the striatum and motor cortex (Bregma = 0.345 ±

0.1 mm) by counting the mHTT inclusions overlapping with nuclear DAPI (nuclear inclusions) or Neurotrace dye signal (cytoplasmic inclusions). For each mouse, 10 fields of view (FOVs), per section, and two brain sections were analyzed. For the quatification of excitatory synapses in the dorsal striatum (Bregma = -0.2 ± 0.1 mm)…
For each mouse, two brain sections and 10 FOVs per section were analyzed.“

p. 20, lines 557, 560-561 (dendritic spine labeling):
“For dendritic spine density analysis in the dorsal striatum (Bregma = -0.2 ± 0.1 mm), a ROI was manually drawn…
For each mouse, 10 – 28 dendrites were analyzed and 20 µm of each dendrite were considered for analysis.“

7) CAG repeat length in the zQ175 mice is missing and should be provided.

We have performed CAG repeat sizing in zQ175DN mice, the results are now shown in Fig. EV3A. No significant difference was observed between placebo- and anle138b-treated zQ175DN mice.

Referee #2 (Remarks for Author):

Huntington's disease (HD) is a fatal neurodegenerative disorder caused by a CAG repeat expansion in the HTT gene, leading to toxic protein aggregation and brain atrophy. The study by Miguel da Silva Padilha et al., testes the effects of the oligomer-modifying compound anle138b (emrusolmin) to reduce mutant huntingtin (mHTT) aggregation and neurotoxicity in cell cultures, HD mouse models, and patient-derived neural precursor cells. While the study is well-structured and presents compelling data - particularly in the R6/2 transgenic model, which over-expresses a truncated human huntingtin fragment with an expanded CAG repeat - the findings are only modestly validated in the zQ175 knock-in model. The latter is widely recognized as a physiologically relevant mouse model for Huntington's disease (HD), as it more closely recapitulates the full-length endogenous mutation seen in patients. This discrepancy somewhat limits the translational strength of the conclusions. The therapeutic potential of anle138b is of significant interest, especially given the current lack of effective treatments that can modify disease progression or delay onset in HD. However, a clearer link between the reduction of aggregates and key functional outcomes-such as somatic instability, transcriptional dysregulation, and neuronal loss-is needed to better define the therapeutic relevance of anle138b.

We thank the reviewer for the positive comments and constructive feedback.

Specifically, several critical limitations temper the overall enthusiasm for the findings:

1) Lack of Assessment of Somatic Instability: Emerging research highlights somatic CAG repeat instability - particularly in medium spiny neurons of the striatum - as a key determinant of neuronal vulnerability in HD. Given that aggregate toxicity and oxidative stress have been linked to increased somatic instability and neuronal loss, a relevant point that was not considered by the authors is whether anle138b modulates somatic instability. Integrating this analysis would provide valuable insight into the compound's potential disease-modifying effects.

We thank the reviewer for the helpful suggestion. We agree that assessment of somatic instability would be a very valuable addition to the study. Unfortunately, we did not have a sufficient number of animals to collect tissue for analysis of somatic

instability within the time frame of the revisions. We have acknowledged in the manuscript that these experiments would be an important direction for further studies (p. 14, lines 375-378):

"In addition, a crucial pathogenic mechanism of HD that has recently come into focus is somatic instability of CAG repeats (Handsaker et al., 2025; Tabrizi et al., 2020). It will therefore be important in future studies to also investigate the ability of anle138b to modulate somatic repeat expansion."

2) Need for Direct Comparative Analysis Between Models: The use of both the R6/2 and zQ175 models is a strength of the study; however, a more structured, side-by-side comparison of phenotypic and molecular responses between the two models would greatly enhance the interpretability and translational relevance of the results. Highlighting both convergent and divergent outcomes could yield deeper insights into the compound's mode of action across disease stages and genetic contexts.

We note that the majority of analyses were the same in both models: brain atrophy, astrogliosis, microgliosis, inclusion quantification by immunostaining, aggregate load by filter trap, Western blots for DARPP-32 and PDE10A. In the revised manuscript, we have also performed synaptic stainings in zQ175DN mice to complement the set of analyses for this model (Fig. EV5H-J). We did not detect significant changes in synaptic marker densities in this mouse model, and therefore could not assess the impact of anle138b on this parameter.

Behavioral defects and life span were only quantified in R6/2 mice. These analyses were precluded in zQ175DN mice by a lack of significant impairments, based on our findings in a previous study (Voelkl et al., *Life Sci Alliance* 2023).

We have included an overview table (Appendix Table S1) listing the findings in both models side by side to provide a clear and structured comparison.

Model	R6/2 (12 weeks)	zQ175DN (9 months)
Motor behavior and life span	Grip Strength - **Significant improvement**<br>Rotarod - **Significant improvement**<br>Open field - **Significant improvement**<br>Limb clasping - **Significant improvement**<br>Life span - **Significant increase in females**	**No motor phenotype** was observed until 12 months of age [19]. Heterozygous zQ175DN mice **do not display reduced survival** [28]
Brain atrophy	Forebrain area - **Significant improvement**<br>Whole brain volume - Non-significant improvement<br>Ventricular volume - **Significant improvement**	Forebrain area - **No phenotype**<br>Whole brain volume - **No phenotype**<br>Ventricular volume - **No phenotype**
Neuroinflammation	Astrogliosis - **Significant improvement**<br>Microgliosis - Non-significant improvement	Astrogliosis - **No phenotype**<br>Microgliosis - **No phenotype**
mHTT aggregate load	Nuclear inclusions - **Significant improvement**<br>SDS-insoluble inclusions - **Significant improvement**	Nuclear inclusions - Non-significant improvement<br>SDS-insoluble inclusions - **Significant improvement**
Striatal markers	DARPP-32 - **Significant improvement**<br>PDE10A - **Significant improvement**	DARPP-32 - **No phenotype**<br>PDE10A - **Improvement**
Synapse density	Dendritic spine density - **Significant improvement**<br>Excitatory synapses - Non-significant improvement	Excitatory synapses - **No phenotype**

Appendix Table S1. *Summary of the effects of anle138b's on HD-related phenotypes in the R6/2 and zQ175DN mouse models.*

We have also included a summary of similarities and differences of the two models into the Discussion (p. 11, lines 305-313):

"The zQ175DN knock-in model only displayed few significant phenotypes (mHTT aggregation and decreased PDE10A protein levels). The effect of anle138b on those phenotypes was in agreement with the findings in the R6/2 model (Appendix Table S1). Moreover, we demonstrate that able138b ameliorated transcriptional alterations in this line already at the preclinical age of 6 months after only 2 months of treatment. In the R6/2 line, we additionally show that anle138b partially rescued motor impairments seen at the advanced disease stage, prolonged life span, reduced brain atrophy, astrogliosis and synapse loss (Appendix Table S1). The mild nature of the zQ175DN knock-in model precluded the assessment of anle138b effects on these phenotypes in this mouse line (Voelkl et al., 2023)."

3) High Phenotypic Variability in some phenotypes: Several phenotypic readouts in the R6/2 mice (Fig. 4) and zQ175 (Fig. 7) exhibit substantial inter-animal variability, which raises concerns about reproducibility and statistical robustness. Increasing the sample size in these cohorts would help to better interpret the observed trends.

We note that high phenotypic variability is inherent to diseases in the progressive stage. We have increased the sample size from n=3 to n=4 for the filter trap assay in zQ175DN mice (Fig. 7B-C). We unfortunately did not have additional R6/2 animals or tissue available to increase the sample size in Fig. 4A-B.

4) Transcriptomic Rescue in R6/2 and zQ175 Not Explored: Previous studies have demonstrated widespread transcriptional dysregulation in R6/2 and zQ175 mice, particularly around 4-6 months of age. Investigating whether anle138b can reverse or mitigate these genome-wide molecular alterations would significantly strengthen the claim of its disease-modifying potential and provide important insight into its mechanism of action.

We have performed transcriptomic analysis of striatal tissue in zQ175DN and littermate control mice. The mice were treated with anle138b or placebo starting from 4 months as in other experiments, and sacrificed at 6 months, an early time point in disease progression when transcriptional dysregulation has been reported in this model (Langfelder et al., 2016), as pointed out by the reviewer. Transcriptional alterations that we observed in the zQ175DN mice at this age were overall mild. Nevertheless, we detected beneficial effects of anle138b on the transcriptional profile of the striatal tissue. The results are described in the text on p. 10-11, lines 263-283, and presented in Fig. 7D-G and Appendix Fig. S4. See also Reviewer #1, comment 1.

5) Additional cellular and molecular phenotypes to be explored in human iPSCs: To strengthen the conclusion regarding the beneficial effects of anle138b in rescuing HD-relevant functional phenotypes, the phenotypic analysis in iPSC-derived neuronal progenitors could be expanded to include additional cellular and molecular readouts. These may include BDNF trafficking, cell adhesion properties, and transcriptomic alterations, given their established relevance in HD pathology.

To strengthen the findings in human HD-iPSCs, we have included an additional iPSC line, Q180, as well as an isogenic control for this line. To support our results in the filter trap assay with an orthogonal approach, we have performed immunostaining for mHTT inclusions and quantified inclusion load by microscopy. We found that in the presence of anle138b, the fraction of NPCs with mHTT inclusion was significantly reduced (Fig. 8D-E).

We believe that further proposed cellular / molecular readouts in iPSCs, such as BDNF trafficking and cell adhesion properties, while very interesting to explore, are outside the scope of the current project, since they would require extensive optimization and would extend beyond the time frame of the revisions.

To investigate which transcriptomic alterations are rescued by anle138b, we have conducted RNA-seq profiling of zQ175DN mice (see response to the previous comment).

Minor points:

6) While the volumetric analyses of the striatum, cortex, and ventricles are relevant and encouraging, the study would benefit from incorporating more refined and sensitive methodologies, such as high-resolution structural MRI. This would allow for a more quantitative and nuanced assessment of brain atrophy, enabling the detection of subtle but biologically meaningful changes that may not be apparent with standard histological techniques.

We thank the reviewer for the helpful suggestion. We were not able to perform the proposed experiments within the time frame of revisions, but will make sure to incorporate this approach in future studies.

7) The analysis of astrogliosis and microglia activation in the mouse brain parenchyma could benefit from an integrative quantitative analysis through western blot experiments, similar to what the authors performed for DARPP32.

As recommended by the reviewer, we have carried out Western blot for Iba1 and GFAP in the cortical and striatal tissue of R6/2 and zQ175DN mice. The results obtained by Western blot align very well with the immunostainings: in R6/2 mice, GFAP protein levels were significantly increased in placebo-treated, but not in anle138b-treated animals (Fig. EV2C-D). We also observed a clear trend for increased Iba1 levels in placebo-treated R6/2 mice, which did not reach statistical significance. In contrast, anle138b-treated animals, Iba1 protein levels were very similar to wildtype controls (Fig. EV2E-F). These new results are described on p. 7, lines 176-181.

In zQ175DN mice, the changes in GFAP and Iba1 levels by Western blot were not significant, therefore potential positive effect of anle138b could not be assessed (Fig. EV4E-H, text p. 9, lines 248-249).

8) Considering the current focus on DNA repair, somatic instability of the expanded CAG tract as major contributors to the HD pathology, a discussion integrating these points with the relevance of treating aggregation in HD would be recommended.

We agree that this is a very relevant point, and have included the following sentences into the discussion (p. 14, lines 375-378):

"In addition, a crucial pathogenic mechanism of HD that has recently come into focus is somatic instability of CAG repeats (Handsaker et al., 2025; Tabrizi et al., 2020). It will therefore be important in future studies to also investigate the ability of anle138b to modulate somatic repeat expansion."

Referee #3 (Comments on Novelty/Model System for Author):

1) This study uses 4 different models: primary mouse neurons, 2 different mice models (R6/2 and zQ175DN knock-in HD mice) and iPSC derived NPC from 1 HD patient. The rational for the specific choice of models remains vague.

We have used primary mouse neurons for a first assessment of anle138b effects in a physiologically relevant *in vitro* system before embarking on animal experiments. We then opted for two complementary mouse models, both well-characterized and widely used in the field: the early-onset, juvenile HD-like R6/2 model with a shortened life span and clear behavioral deficits; and the late-onset zQ175DN model with mild phenotypes, but high genetic validity due to the expression of full-length mHTT from the endogenous locus. The HD-iPSC culture model was used as an important human patient-derived model system to extend our findings to the context of human disease. We have rephrased the text in several places to explain the rationale for using these model systems more clearly:

p. 4, lines 78-82 "*Here, we show that anle138b reduces mHTT aggregation in cultured primary neurons, two complementary mouse models of HD (the early-onset transgenic R6/2 model with a severe phenotype and the late-onset zQ175DN knock-in model that is genetically similar to patients), as well as in human neural precursor cells (NPCs) differentiated from HD patients-derived induced pluripotent stem cells (iPSCs).*"

p. 4, lines 88-89 *"To assess the effects of anle138b in the context of HD, we first employed a cellular model system and assessed mHTT-dependent phenotypes in primary neuronal cultures isolated from mouse embryos".*

p. 9, line 234-235 *"Having observed a clear disease-modifying effect of anle138b in R6/2 mice, we sought to validate these findings in another HD model more similar to adult-onset HD."*

p. 11, line 286-287 *"Having observed positive effects of anle138b in murine neurons and HD mouse models, we aimed to validate these findings in a human model system derived from HD patients".*

Referee #3 (Remarks for Author):

The paper reports that the oligomer-modifying molecule anle138b improves multiple disease phenotypes in cell culture and two HD mouse models. Anle138b reduces mHTT aggregate formation and neurotoxicity in primary neurons. In R6/2 mice, oral administration improves motor performance and survival in females, decreases GFAP-positive cells and mHTT aggregates in the striatum and motor cortex, and enhances striatal markers. At 13 weeks, spine density and VGlut1/PSD-95 puncta increase following treatment.

2) mHTT reduction effects were also observed in zQ175DN mice and one human iPSC derived NPC line. While anle138b shows potential for disease modification, the results remain preliminary. The use of four models with different analytical parameters raises concerns about selective data presentation. Models should be clearly indicated when multiple are used in a figure.

Wherever possible, we have used the same analyses for the different model systems. In particular, analyses of brain atrophy, astrogliosis, microgliosis, inclusion quantification by immunostaining, aggregate load by filter trap, Western blots for DARPP-32 and PDE10A were performed in both R6/2 and zQ175DN mice. In the

revised version of the manuscript, we have also added quantification of synaptic markers by immunostaining in zQ175DN mice to match these analyses in R6/2 mice. Moreover, we have performed quantification of GFAP and Iba1 levels by Western blots in both models.

Behavioral defects and life span were only quantified in R6/2 mice, because these analyses were precluded in zQ175DN mice by a lack of significant impairments (Voelkl et al., *Life Sci Alliance* 2023).

We have included an overview table (Appendix Table S1) listing the findings in both mouse models side by side to provide a clear and structured comparison. We have also included a summary of similarities and differences of the two models into the Discussion (p. 11-12, lines 305-313):

"The zQ175DN knock-in model only displayed few significant phenotypes (mHTT aggregation and decreased PDE10A protein levels). The effect of anle138b on those phenotypes was in agreement with the findings in the R6/2 model (Appendix Table S1). Moreover, we demonstrate that able138b ameliorated transcriptional alterations in this line already at the preclinical age of 6 months after only 2 months of treatment. In the R6/2 line, we additionally show that anle138b partially rescued motor impairments seen at the advanced disease stage, prolonged life span, reduced brain atrophy, astrogliosis and synapse loss (Appendix table S1). The mild nature of the zQ175DN knock-in model precluded the assessment of anle138b effects on these phenotypes in this mouse line (Voelkl et al., 2023)."

For the cell culture models, neurotoxicity analysis and quantification of mHTT inclusions were performed in primary neurons, while mHTT aggregation in HD-iPSC-derived NPCs was probed by filter trap. To be consistent, we have attempted filter trap in primary neuronal cultures, however, due to the low transfection rates of primary neurons we could not detect aggregated mHTT in sufficient amounts for a convincing rescue. Conversely, we have performed quantification of mHTT inclusions by immunostaining in NPCs – these new data are presented in Fig. 8D-E and described in the text on p. 11, lines 294-297.

Our findings in HD-NPCs demonstrate lack of cell death (see Fig. R1 below), consistent with previous reports (Koyuncu et al., 2018). Therefore, assessment of anle138b impact on mHTT toxicity is not possible in this system. We have clarified this point in the text (p. 11, lines 290-291).

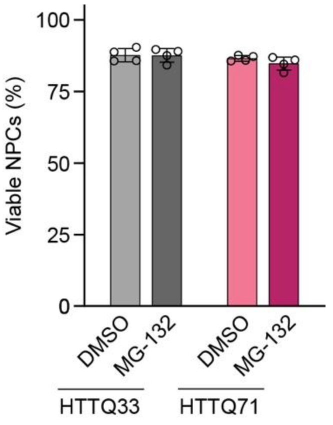

Figure R1. *Quantification of the fraction of viable NPCs in the indicated conditions. mHTT aggregation was induced by proteasome inhibition with 5 μM MG-132 for 8 h. DMSO treatment was used as vehicle control. Viability was assessed by immunostaining against the apoptotic marker cleaved caspase-3. N=4 experiments.*

3) R6/2 data suggest anle138b's positive effects are mostly in female mice and only at specific (late) ages. The current interpretation overstates the findings and should be more cautious. Further analysis in older zQ175DN knock-in mice is needed.

Except for survival, the positive effects have been observed in mice of either sex. To illustrate this more clearly, we now show the behavioral analyses separated by sex in Appendix Fig. S2A-H. In all the tests, the beneficial effects of anle138b are seen in both male and female mice.

At the age of 8 weeks, most of the phenotypes in R6/2 mice are still rather mild, it is therefore difficult to detect a rescue effect. However, in case of a clear phenotype (e.g., fraction of neurons with mHTT inclusions, Figure 4B), a positive effect of anle138b was obvious. Nevertheless, we have made text changes to provide a more cautious interpretation of the findings, as suggested by the reviewer:

Discussion, p. 11, lines 309-310: *"..anle138b partially rescued motor impairments seen at the advanced disease stage…"*

We agree that further analyses in older zQ175DN mice would be helpful. However, we were not able to perform such analyses within the time frame of the revisions. As the model is a slow-progressing one and we have already assessed it at the age of 9 months, these experiments would require more than a year to perform.

4) The HD patient-derived iPSC model could help assess anle138b's impact in human disease, but the current data are very preliminary. Analysis should be conducted in differentiated, disease-relevant neurons from a sufficient number of HD patients and controls, including detailed morphological assessments (neurite integrity, proteostasis, cell death, transcriptome, and proteome). Clinically relevant mHTT measures should be included to estimate translational potential.

To strengthen the findings in human HD-iPSCs, we have included an additional iPSC line, Q180, as well as an isogenic control for this line. To support our results in the filter trap assay with an orthogonal approach, we have performed immunostaining for mHTT inclusions and quantified inclusion load by microscopy. We found that in the presence of anle138b, the fraction of NPCs with mHTT inclusion was significantly reduced (Fig. 8D-E).

Our findings in HD-NPCs demonstrate lack of cell death (see Fig. R1 above), consistent with previous reports (Koyuncu et al., 2018). Therefore, assessment of anle138b impact on cell death is not possible in this system. We have clarified this point in the text (p. 11, lines 290-291).

We believe that differentiation of iPSCs into mature neurons and further proposed cellular / molecular readouts, such as neurite integrity and proteomics, while very interesting to explore, are outside the scope of the current project, since they would require extensive optimization and would extend beyond the time frame of the revisions.

To investigate which transcriptomic alterations are rescued by anle138b, we have conducted RNA-seq profiling of zQ175DN mice. The mice were treated with anle138b or placebo starting from 4 months as in other experiments, and sacrificed at 6 months, an age when transcriptional dysregulation has been reported in this model (Langfelder et al., 2016). Transcriptional alterations that we observed in the zQ175DN mice at this age were overall mild. Nevertheless, we detected beneficial effects of anle138b on the transcriptional profile of the striatal tissue. The results are described in the text on p.

10-11, lines 263-283, and presented in Fig. 7D-G and Appendix Fig. S4. See also Reviewer #1, comment 1 and Reviewer #2, comment 4.

Minor issues:

5) Why are different magnifications used in panels A and D? Standardizing them would improve consistency.

The reviewer presumably refers to Fig. 1. Thank you for the comment. We have replaced the images in panel D with the same magnification as in A for consistency.

In addition to the changes requested by the reviewers, we have also included dose-response experiments in primary neurons with different concentrations of anle138b, demonstrating that the compound is efficient at even lower concentrations (starting from 500 nM) than those used in all previous experimental studies. These concentrations are similar to the ones reached in humans in the phase 1 trial. These new data are presented in Fig. EV1 and described in the text on p. 4, lines 100-104.

References

Albariqi, M.M.H., S.M.G. Baauw, S.J.P.J. Fens, S. Versteeg, S. Ryazanov, A. Leonov, H.L.D.M. Willemen, N. Stathonikos, R.M. Seychell, A. El Saghir, B. Gerritsen, L. Khemtemourian, N. Vassallo, A. Giese, N. Eijkelkamp, C. Griesinger, and J.W.M. Höppener. 2024. The amyloid oligomer modulator anle138b has disease modifying effects in a human IAPP transgenic mouse model of type 2 diabetes mellitus (hIAPP Ob/Ob mice). *bioRxiv*.

Consortium, H.D.i. 2012. Induced pluripotent stem cells from patients with Huntington's disease show CAG-repeat-expansion-associated phenotypes. *Cell Stem Cell*. 11:264-278.

Handsaker, R.E., S. Kashin, N.M. Reed, S. Tan, W.S. Lee, T.M. McDonald, K. Morris, N. Kamitaki, C.D. Mullally, N.R. Morakabati, M. Goldman, G. Lind, R. Kohli, E. Lawton, M. Hogan, K. Ichihara, S. Berretta, and S.A. McCarroll. 2025. Long somatic DNA-repeat expansion drives neurodegeneration in Huntington's disease. *Cell*. 188:623-639 e619.

Heras-Garvin, A., D. Weckbecker, S. Ryazanov, A. Leonov, C. Griesinger, A. Giese, G.K. Wenning, and N. Stefanova. 2019. Anle138b modulates alpha-synuclein oligomerization and prevents motor decline and neurodegeneration in a mouse model of multiple system atrophy. *Mov Disord*. 34:255-263.

Hosp, F., S. Gutierrez-Angel, M.H. Schaefer, J. Cox, F. Meissner, M.S. Hipp, F.U. Hartl, R. Klein, I. Dudanova, and M. Mann. 2017. Spatiotemporal Proteomic Profiling of Huntington's Disease Inclusions Reveals Widespread Loss of Protein Function. *Cell Rep*. 21:2291-2303.

Koyuncu, S., I. Saez, H.J. Lee, R. Gutierrez-Garcia, W. Pokrzywa, A. Fatima, T. Hoppe, and D. Vilchez. 2018. The ubiquitin ligase UBR5 suppresses proteostasis collapse in pluripotent stem cells from Huntington's disease patients. *Nat Commun*. 9:2886.

Langfelder, P., J.P. Cantle, D. Chatzopoulou, N. Wang, F. Gao, I. Al-Ramahi, X.H. Lu, E.M. Ramos, K. El-Zein, Y. Zhao, S. Deverasetty, A. Tebbe, C. Schaab, D.J. Lavery, D. Howland, S. Kwak, J. Botas, J.S. Aaronson, J. Rosinski, G. Coppola, S. Horvath, and X.W. Yang. 2016. Integrated genomics and proteomics define huntingtin CAG length-dependent networks in mice. *Nature neuroscience*. 19:623-633.

Martinez Hernandez, A., H. Urbanke, A.L. Gillman, J. Lee, S. Ryazanov, H.Y. Agbemenyah, E. Benito, G. Jain, L. Kaurani, G. Grigorian, A. Leonov, N. Rezaei-Ghaleh, P. Wilken, F.T. Arce, J. Wagner, M. Fuhrmann, M. Caruana, A. Camilleri, N. Vassallo, M. Zweckstetter, R. Benz, A. Giese, A. Schneider, M. Korte, R. Lal, C. Griesinger, G. Eichele, and A. Fischer. 2018. The diphenylpyrazole compound anle138b blocks Abeta channels and rescues disease phenotypes in a mouse model for amyloid pathology. *EMBO Mol Med*. 10:32-47.

Noormohammadi, A., A. Khodakarami, R. Gutierrez-Garcia, H.J. Lee, S. Koyuncu, T. Konig, C. Schindler, I. Saez, A. Fatima, C. Dieterich, and D. Vilchez. 2016. Somatic increase of CCT8

mimics proteostasis of human pluripotent stem cells and extends C. elegans lifespan. *Nat Commun*. 7:13649.

Tabrizi, S.J., M.D. Flower, C.A. Ross, and E.J. Wild. 2020. Huntington disease: new insights into molecular pathogenesis and therapeutic opportunities. *Nat Rev Neurol*.

Voelkl, K., S. Gutierrez-Angel, S. Keeling, S. Koyuncu, M. da Silva Padilha, D. Feigenbutz, T. Arzberger, D. Vilchez, R. Klein, and I. Dudanova. 2023. Neuroprotective effects of hepatoma-derived growth factor in models of Huntington's disease. *Life Sci Alliance*. 6.

Wagner, J., S. Krauss, S. Shi, S. Ryazanov, J. Steffen, C. Miklitz, A. Leonov, A. Kleinknecht, B. Goricke, J.H. Weishaupt, D. Weckbecker, A.M. Reiner, W. Zinth, J. Levin, D. Ehninger, S. Remy, H.A. Kretzschmar, C. Griesinger, A. Giese, and M. Fuhrmann. 2015. Reducing tau aggregates with anle138b delays disease progression in a mouse model of tauopathies. *Acta Neuropathol*. 130:619-631.

Wagner, J., S. Ryazanov, A. Leonov, J. Levin, S. Shi, F. Schmidt, C. Prix, F. Pan-Montojo, U. Bertsch, G. Mitteregger-Kretzschmar, M. Geissen, M. Eiden, F. Leidel, T. Hirschberger, A.A. Deeg, J.J. Krauth, W. Zinth, P. Tavan, J. Pilger, M. Zweckstetter, T. Frank, M. Bahr, J.H. Weishaupt, M. Uhr, H. Urlaub, U. Teichmann, M. Samwer, K. Botzel, M. Groschup, H. Kretzschmar, C. Griesinger, and A. Giese. 2013. Anle138b: a novel oligomer modulator for disease-modifying therapy of neurodegenerative diseases such as prion and Parkinson's disease. *Acta Neuropathol*. 125:795-813.

Wegrzynowicz, M., D. Bar-On, L. Calo, O. Anichtchik, M. Iovino, J. Xia, S. Ryazanov, A. Leonov, A. Giese, J.W. Dalley, C. Griesinger, U. Ashery, and M.G. Spillantini. 2019. Depopulation of dense alpha-synuclein aggregates is associated with rescue of dopamine neuron dysfunction and death in a new Parkinson's disease model. *Acta Neuropathol*. 138:575-595.

4th May 2026

Dear Irina,

Thank you for the submission of your revised manuscript to EMBO Molecular Medicine. We have now received the enclosed reports from the two referees who were asked to reassess your manuscript. As you will see, both referees agree that the revision has improved the study, although they have raised several - relatively minor- concerns.

Before I can accept your manuscript, pending the amendments outlined below, please address the following issues:

- The remaining comment from Referee #1, and comments #2 and #4 from Referee #2.

On a more editorial level:

1. The email to David Vilchez (dvilchez01@gmail.com) was undeliverable. Please provide a valid email address.

2. Please remove the "Authors' Contribution" section.

3. Reference format: Limit the number of author names to 10, followed by et al.

4. Please provide a "Synopsis" (as a separate DOCX file) to enhance discoverability. Synopses are displayed on the journal webpage and are freely accessible to all readers. They include a short stand first (maximum of 300 characters, including space) as well as 2-5 one-sentences bullet points that summarizes the paper. Please write the bullet points to summarize the key NEW findings. They should be designed to be complementary to the abstract - i.e. not repeat the same text. We encourage inclusion of key acronyms and quantitative information (maximum of 30 words / bullet point). Please use the passive voice. Please attach these in a separate file or send them by email, we will incorporate them accordingly.

5. Please move "the paper explained" to the main manuscript file.

6. Appendix: Please remove the yellow font. On the first page, please add "Appendix for [Manuscript Title]."

7. Author checklist: response in line 80 is missing, please complete it.

8.There is a callout for Table EV1, but no corresponding file is provided. Please resolve this discrepancy.

9. During our routine source data check, we identified potential duplication within the source data for Figure 1E (see attached). Could you please review the highlighted cells and provide clarification on these duplicates?

10. Please address the following issues in figure legends:
- Please note that the exact p values are not provided in the legends of figures 1B, C, E; 2B, C, E, F; 3D, F; 4B, D; 6B, D, E; 7C,8E, EV1 A, B; S1B, D; S2 A-C, D-F, H; S3A-C; S5 B
- Please note that in figures 6B, EV2 D there is a mismatch between the annotated p values in the figure legend and the annotated p values in the figure file that should be corrected.
- Please note that information related to n is missing in the legends of figures 2B, C, E, F, H; 7E-G
-Please note that the error bars are not defined in the legends of figures EV1 A, B; S1 A, B

11. Please correct the following section headings: Competing Interests Statement => Disclosure and Competing Interests Statement; Materials and Methods => Methods

I look forward to seeing a revised form of your manuscript as soon as possible.

Kind regards,
Jingyi

Jingyi Hou
Senior Editor
EMBO Molecular Medicine

*** Instructions to submit your revised manuscript ***

*** PLEASE NOTE *** As part of the EMBO Publications transparent editorial process initiative (see our Editorial at https://www.embopress.org/doi/pdf/10.1002/emmm.201000094), EMBO Molecular Medicine will publish online a Review Process File to accompany accepted manuscripts.

In the event of acceptance, this file will be published in conjunction with your paper and will include the anonymous referee reports, your point-by-point response and all pertinent correspondence relating to the manuscript. If you do NOT want this file to be published, please inform the editorial office at contact@embomolmed.org.

*****

When preparing your revised manuscript, please refer to our guidelines: https://link.springer.com/journal/44321/submission-guidelines#cms-Revised-submissions. We perform an initial quality control of all revised manuscripts before re-review; failure to include requested items will delay the evaluation of your revision.

***** Reviewer's comments *****

Referee #1 (Remarks for Author):

The Authors have adequately addressed all my raised concerns, and I have no additional comments. However, because the Western blots were not performed as technical replicates, this information should be included and/or considered at the editorial level based on the journal policy on this matter.

Referee #2 (Comments on Novelty/Model System for Author):

The authors have improved the manuscript by providing a more rigorous comparative framework and expanding their experimental models. A key strength of the revision is the structured, side-by-side characterization of the R6/2 and zQ175DN animal models now detailed in Appendix S1. This comparison successfully highlights the more attenuated behavioral phenotype of the zQ175DN line while identifying specific, rescued phenotypes that demonstrate the therapeutic potential of anle138b. This animal work is further supported by the addition of the Q180 human iPSC line. By including this line with a strongly extended CAG repeat, the authors have provided a more robust validation of phenotypic amelioration in a human context, particularly regarding the reduction of protein aggregation.

Referee #2 (Remarks for Author):

The authors have clearly improved the manuscript by addressing the key experimental requests. They have provided a more structured comparison of animal models, performed transcriptomic analysis, and utilized additional cellular models to validate their findings.
The following summary outlines the primary revisions and the areas where further clarification is requested.

1. Comparative Analysis of Animal Models
The authors successfully addressed the request for a side-by-side comparison between the R6/2 and zQ175DN models.
Appendix S1: A comprehensive table comparing the phenotypes of these two models has been added. The data highlights that the zQ175DN model generally exhibits milder behavioral abnormalities compared to R6/2. However, the authors have successfully identified and characterized few specific rescued phenotypes within this model.
2. Transcriptomic Analysis in zQ175DN Mice
A major addition to the revised paper is the bulk RNA-seq analysis of the striatum and cortex in zQ175DN mice to assess the molecular rescue potential of anle138b. While the inclusion of this complex experiment is appreciated, several technical concerns remain regarding the results:
> Limited Differential Expression: The study reports only 12 differentially expressed genes (DEGs) in the striatum between WT and zQ175DN animals. This is notably lower than previous standards, such as Langfelder et al. (2018) and Lee et al., 2020 (TRAP technology), which identified thousands of DEGs at the same time points. Because of this extremely low level of DEG in the reference conditions (WT versus zQ175DN placebo), then the reduction in the DEG observed after anle138b treatment is

quite uncertain and the results not convincing.
Some methodological biases might affect the results:
> Sample Size: The legend for Figure 7 indicates n=3-7. It is unclear which groups utilized the lower end of this range (n=3), which may cause issues on the statistical power to detect subtle transcriptomic changes.
> Sequencing Technique: The use of 3' RNA-seq may limit the sensitivity of the analysis compared to standard full-length bulk RNA-seq.
> High variability between different biological replicates: Could the authors show the QC and the PCA of the newly produced data?

The authors should explicitly state the sample size for each experimental group, discuss the potential biases introduced by the 3' RNA-seq technique, and provide a detailed comparison/discussion regarding why their results differ so drastically from the established zQ175 and zQ175DN transcriptomic landscape.
A clustering of the heatmap focusing on the differences detected between WT and zQ175DN will be valuable to better appreciate how comparable are the DEG profiles of the zQ175DN treated with placebo and the % of transcriptional rescue obtained by treating with anle138b.

3. iPSC Characterization and Functional Phenotypes
To reinforce the evidence for anle138b's efficacy, the authors expanded their cellular models:
> New Cell Line: The inclusion of the Q180 iPSC line provides more robust evidence across a different strongly extended CAG repeat.
> Phenotypic Amelioration: Further characterization of the reduction in aggregation phenotypes was provided, addressing the need for broader molecular readouts in human-derived models.
4. Quantitative Analysis of Gliosis
In response to the request for integrative quantitative analysis of neuroinflammation:
> Western Blotting: The authors provided Western Blot (WB) data for astrogliosis and microglia activation in Extended View 2 (EV2).
The current quality of the WB images in EV2 is insufficient for reliable quantification. The authors are encouraged to provide higher-resolution blots or alternative quantitative data to support these findings.

Point-by-point response to reviewers' comments

Referee #1 (Remarks for Author):

The Authors have adequately addressed all my raised concerns, and I have no additional comments. However, because the Western blots were not performed as technical replicates, this information should be included and/or considered at the editorial level based on the journal policy on this matter.

We thank the reviewer for the positive evaluation.
We have added a sentence in the Methods section to clarify that the Western blots were not performed as technical replicates (p. 22, line 629-630):

> "For each experiment, 3 – 4 biological replicates per genotype and treatment group were analyzed. One technical replicate was performed for each mouse."

Referee #2

The authors have improved the manuscript by providing a more rigorous comparative framework and expanding their experimental models. A key strength of the revision is the structured, side-by-side characterization of the R6/2 and zQ175DN animal models now detailed in Appendix S1. This comparison successfully highlights the more attenuated behavioral phenotype of the zQ175DN line while identifying specific, rescued phenotypes that demonstrate the therapeutic potential of anle138b. This animal work is further supported by the addition of the Q180 human iPSC line. By including this line with a strongly extended CAG repeat, the authors have provided a more robust validation of phenotypic amelioration in a human context, particularly regarding the reduction of protein aggregation.

Referee #2 (Remarks for Author):

The authors have clearly improved the manuscript by addressing the key experimental requests. They have provided a more structured comparison of animal models, performed transcriptomic analysis, and utilized additional cellular models to validate their findings.

We thank the reviewer for highlighting the strengths of the revised manuscript.

The following summary outlines the primary revisions and the areas where further clarification is requested.

1. Comparative Analysis of Animal Models
The authors successfully addressed the request for a side-by-side comparison between the R6/2 and zQ175DN models.

Appendix S1: A comprehensive table comparing the phenotypes of these two models has been added. The data highlights that the zQ175DN model generally exhibits milder behavioral abnormalities compared to R6/2. However, the authors have successfully identified and characterized few specific rescued phenotypes within this model.

We are happy to hear that the table comparing the findings in the R6/2 and zQ175DN mouse models is helpful.

2. Transcriptomic Analysis in zQ175DN Mice
A major addition to the revised paper is the bulk RNA-seq analysis of the striatum and cortex in zQ175DN mice to assess the molecular rescue potential of anle138b. While the inclusion of this complex experiment is appreciated, several technical concerns remain regarding the results:
> Limited Differential Expression: The study reports only 12 differentially expressed genes (DEGs) in the striatum between WT and zQ175DN animals. This is notably lower than previous standards, such as Langfelder et al. (2018) and Lee et al., 2020 (TRAP technology), which identified thousands of DEGs at the same time points. Because of this extremely low level of DEG in the reference conditions (WT versus zQ175DN placebo), then the reduction in the DEG observed after anle138b treatment is quite uncertain and the results not convincing. Some methodological biases might affect the results:
> Sample Size: The legend for Figure 7 indicates n=3-7. It is unclear which groups utilized the lower end of this range (n=3), which may cause issues on the statistical power to detect subtle transcriptomic changes.
> Sequencing Technique: The use of 3' RNA-seq may limit the sensitivity of the analysis compared to standard full-length bulk RNA-seq.
> High variability between different biological replicates: Could the authors show the QC and the PCA of the newly produced data?

The authors should explicitly state the sample size for each experimental group, discuss the potential biases introduced by the 3' RNA-seq technique, and provide a detailed comparison/discussion regarding why their results differ so drastically from the established zQ175 and zQ175DN transcriptomic landscape.
A clustering of the heatmap focusing on the differences detected between WT and zQ175DN will be valuable to better appreciate how comparable are the DEG profiles of the zQ175DN treated with placebo and the % of transcriptional rescue obtained by treating with anle138b.

We thank the reviewer for the detailed comments and constructive suggestions on the interpretation of the RNA-seq data.
We agree with the limitations of our approach and have included the following text into the discussion (p. 12, lines 337-342):

> "It should be mentioned that the transcriptional changes detected in our study in 6-month-old zQ175 mice were very mild compared to previous findings (Langfelder *et al.*, 2016; Lee *et al*, 2020). This might be due to the low sample size, colony differences and/or methodological aspects, such as the use of 3' RNA-seq technique that has limited sensitivity compared to full-length bulk RNA-seq. Nevertheless, the subtle transcriptional dysregulation that we were able to detect was partially rescued by anle138b treatment."

We have stated the exact number of animals per group in the legend for Fig. 7 (page 34, lines 1086-1087):

> "For all transcriptomic analyses shown in (D - G), n = 4 WT, 3 WT + anle138, 7 zQ175DN and 4 zQ175DN + anle138 mice."

As requested by the reviewer, we now show the QC of the RNA-seq data in the source data for Fig. 7.

We have also included a heatmap (Fig. 7D, right) focusing on the genes differentially expressed between the WT and zQ175DN groups and showing their rescue by anle138b treatment.

3. iPSC Characterization and Functional Phenotypes
To reinforce the evidence for anle138b's efficacy, the authors expanded their cellular models:
> New Cell Line: The inclusion of the Q180 iPSC line provides more robust evidence across a different strongly extended CAG repeat.
> Phenotypic Amelioration: Further characterization of the reduction in aggregation phenotypes was provided, addressing the need for broader molecular readouts in human-derived models.

We thank the reviewer for acknowledging the value of the additional iPSC experiments.

4. Quantitative Analysis of Gliosis
In response to the request for integrative quantitative analysis of neuroinflammation:
> Western Blotting: The authors provided Western Blot (WB) data for astrogliosis and microglia activation in Extended View 2 (EV2).
The current quality of the WB images in EV2 is insufficient for reliable quantification. The authors are encouraged to provide higher-resolution blots or alternative quantitative data to support these findings.

We have replaced the Western blot images in Figure EV2 with better-quality ones.

20th May 2026

Dear Irina,

We are pleased to inform you that your manuscript is accepted for publication and is now being sent to our publisher to be included in the next available issue of EMBO Molecular Medicine.

Your manuscript will be processed for publication by EMBO Press. It will be copy edited and you will receive page proofs prior to publication. Please note that you will be contacted by Springer Nature Author Services to complete licensing and payment information.

You may qualify for financial assistance for your publication charges - either via a Springer Nature fully open access agreement or an EMBO initiative. Check your eligibility: https://link.springer.com/journal/44321/how-to-publish-with-us

Should you be planning a Press Release on your article, please notify the production team via this form in order to coordinate publication and release dates: https://forms.cloud.microsoft/e/eH4A8JVP6g

If you have any questions, please do not hesitate to contact the Editorial Office. Thank you for your contribution to EMBO Molecular Medicine.

Yours sincerely,
Jingyi

Jingyi Hou
Senior Editor
EMBO Molecular Medicine

>>> Please note that it is EMBO Molecular Medicine policy for the transcript of the editorial process (containing referee reports and your response letter) to be published as an online supplement to each paper. If you do NOT want this, you will need to inform the Editorial Office via email immediately. More information is available here: https://link.springer.com/partners/embo-press/editorial-policies#Peer%20review

EMBO Press Author Checklist

Corresponding Author Name: Irina Dudanova	
Journal Submitted to: EMBO Molecular Medicine	
Manuscript Number: EMM-2025-21575	

USEFUL LINKS FOR COMPLETING THIS FORM
The EMBO Journal - Author Guidelines
EMBO Reports - Author Guidelines
Molecular Systems Biology - Author Guidelines
EMBO Molecular Medicine - Author Guidelines

Reporting Checklist for Life Science Articles (updated January
This checklist is adapted from Materials Design Analysis Reporting (MDAR) Checklist for Authors. MDAR establishes a minimum set of requirements in transparent reporting in the life sciences (see Statement of Task: 10.31222/osf.io/9sm4x). Please follow the journal's guidelines in preparing your
Please note that a copy of this checklist will be published alongside your article.

Abridged guidelines for figures
1. Data
The data shown in figures should satisfy the following conditions:
- ➞ the data were obtained and processed according to the field's best practice and are presented to reflect the results of the experiments in an accurate and unbiased manner.
- ➞ ideally, figure panels should include only measurements that are directly comparable to each other and obtained with the same assay.
- ➞ plots include clearly labeled error bars for independent experiments and sample sizes. Unless justified, error bars should not be shown for technical
- ➞ if n<5, the individual data points from each experiment should be plotted. Any statistical test employed should be justified.
- ➞ Source Data should be included to report the data underlying figures according to the guidelines set out in the authorship guidelines on Data

2. Captions
Each figure caption should contain the following information, for each panel where they are relevant:
- ➞ a specification of the experimental system investigated (eg cell line, species name).
- ➞ the assay(s) and method(s) used to carry out the reported observations and measurements.
- ➞ an explicit mention of the biological and chemical entity(ies) that are being measured.
- ➞ an explicit mention of the biological and chemical entity(ies) that are altered/varied/perturbed in a controlled manner.
- ➞ the exact sample size (n) for each experimental group/condition, given as a number, not a range;
- ➞ a description of the sample collection allowing the reader to understand whether the samples represent technical or biological replicates (including how many animals, litters, cultures, etc.).
- ➞ a statement of how many times the experiment shown was independently replicated in the laboratory.
- ➞ definitions of statistical methods and measures:
  - common tests, such as t-test (please specify whether paired vs. unpaired), simple χ2 tests, Wilcoxon and Mann-Whitney tests, can be unambiguously identified by name only, but more complex techniques should be described in the methods section;
  - are tests one-sided or two-sided?
  - are there adjustments for multiple comparisons?
  - exact statistical test results, e.g., P values = x but not P values < x;
  - definition of 'center values' as median or average;
  - definition of error bars as s.d. or s.e.m.

Please complete ALL of the questions below.
Select "Not Applicable" only when the requested information is not relevant for your study.

Materials

Newly Created Materials	Information included in the manuscript?	In which section is the information available? (Reagents and Tools Table, Materials and Methods, Figures, Data Availability Section)
New materials and reagents need to be available; do any restrictions apply?	Not Applicable	

Antibodies	Information included in the manuscript?	In which section is the information available? (Reagents and Tools Table, Materials and Methods, Figures, Data Availability Section)
For **antibodies** provide the following information:<br>- Commercial antibodies: RRID (if possible) or supplier name, catalogue number and or/clone number<br>- Non-commercial: RRID or citation	Yes	Materials and Methods

DNA and RNA sequences	Information included in the manuscript?	In which section is the information available? (Reagents and Tools Table, Materials and Methods, Figures, Data Availability Section)
**Short novel DNA or RNA including primers, probes**: provide the sequences.	Not Applicable	

Cell materials	Information included in the manuscript?	In which section is the information available? (Reagents and Tools Table, Materials and Methods, Figures, Data Availability Section)
**Cell lines:** Provide species information, strain. Provide accession number in repository **OR** supplier name, catalog number, clone number, and/**OR** RRID.	Yes	Material and Methods
**Primary cultures:** Provide species, strain, sex of origin, genetic modification status.	Yes	Material and Methods
Report if the cell lines were recently **authenticated** (e.g., by STR profiling) and tested for mycoplasma contamination.	Yes	Material and Methods

Experimental animals	Information included in the manuscript?	In which section is the information available? (Reagents and Tools Table, Materials and Methods, Figures, Data Availability Section)
**Laboratory animals or Model organisms:** Provide species, strain, sex, age, genetic modification status. Provide accession number in repository **OR** supplier name, catalog number, clone number, **OR** RRID.	Yes	Material and Methods
**Animal observed in or captured from the field:** Provide species, sex, and age where possible.	Not Applicable	
Please detail **housing and husbandry conditions**.	Yes	Material and Methods

Plants and microbes	Information included in the manuscript?	In which section is the information available? (Reagents and Tools Table, Materials and Methods, Figures, Data Availability Section)
**Plants:** provide species and strain, ecotype and cultivar where relevant, unique accession number if available, and source (including location for collected wild specimens).	Not Applicable	
**Microbes:** provide species and strain, unique accession number if available, and source.	Not Applicable	

Human research participants	Information included in the manuscript?	In which section is the information available? (Reagents and Tools Table, Materials and Methods, Figures, Data Availability Section)
If collected and within the bounds of privacy constraints report on age, sex and gender or ethnicity for all study participants.	Not Applicable	

Core facilities	Information included in the manuscript?	In which section is the information available? (Reagents and Tools Table, Materials and Methods, Figures, Data Availability Section)
If your work benefited from core facilities, was their service mentioned in the acknowledgments section?	Yes	Acknowlegments

Design

Study protocol	Information included in the manuscript?	In which section is the information available? (Reagents and Tools Table, Materials and Methods, Figures, Data Availability Section)
If study protocol has been **pre-registered, provide DOI in the manuscript**. For clinical trials, provide the trial registration number **OR** cite DOI.	Not Applicable	
Report the **clinical trial registration number** (at ClinicalTrials.gov or equivalent), where applicable.	Not Applicable	

Laboratory protocol	Information included in the manuscript?	In which section is the information available? (Reagents and Tools Table, Materials and Methods, Figures, Data Availability Section)
Provide DOI OR other citation details if **external detailed step-by-step protocols** are available.	Yes	Material and Methods

Experimental study design and statistics	Information included in the manuscript?	In which section is the information available? (Reagents and Tools Table, Materials and Methods, Figures, Data Availability Section)
Include a statement about **sample size** estimate even if no statistical methods were used.	Yes	Materials and Methods
Were any steps taken to minimize the effects of subjective bias when allocating animals/samples to treatment (e.g. **randomization procedure**)? If yes, have they been described?	Yes	Materials and Methods
Include a statement about **blinding** even if no blinding was done.	Yes	Materials and Methods
Describe **inclusion/exclusion criteria** if samples or animals were excluded from the analysis. Were the criteria pre-established?<br><br>If sample or data points were omitted from analysis, report if this was due to attrition or intentional exclusion and provide justification.	Not Applicable	
For every figure, are **statistical tests** justified as appropriate? Do the data meet the assumptions of the tests (e.g., normal distribution)? Describe any methods used to assess it. Is there an estimate of variation within each group of data? Is the variance similar between the groups that are being statistically compared?	Yes	Figure legends

Sample definition and in-laboratory replication	Information included in the manuscript?	In which section is the information available? (Reagents and Tools Table, Materials and Methods, Figures, Data Availability Section)
In the figure legends: state number of times the experiment was **replicated** in laboratory.	Yes	Figure legends
In the figure legends: define whether data describe **technical or biological replicates**.	Yes	Figure legends

Ethics

Ethics	Information included in the manuscript?	In which section is the information available? (Reagents and Tools Table, Materials and Methods, Figures, Data Availability Section)
Studies involving **human participants**: State details of **authority granting ethics approval** (IRB or equivalent committee(s), provide reference number for approval.	Not Applicable	
Studies involving **human participants**: Include a statement confirming that **informed consent** was obtained from all subjects and that the experiments conformed to the principles set out in the WMA Declaration of Helsinki and the Department of Health and Human Services Belmont Report.	Not Applicable	
Studies involving **human participants:** For publication of **patient photos**, include a statement confirming that consent to publish was obtained.	Not Applicable	
Studies involving experimental **animals**: State details of **authority granting ethics approval** (IRB or equivalent committee(s), provide reference number for approval. Include a statement of compliance with ethical regulations.	Yes	Materials and Methods
Studies involving **specimen and field samples:** State if relevant **permits** obtained, provide details of authority approving study; if none were required, explain why.	Not Applicable	

Dual Use Research of Concern (DURC)	Information included in the manuscript?	In which section is the information available? (Reagents and Tools Table, Materials and Methods, Figures, Data Availability Section)
Could your study fall under dual use research restrictions? Please check biosecurity documents and list of **select agents and toxins** (CDC): https://www.selectagents.gov/sat/list.htm	Not Applicable	
If you used a select agent, is the security level of the lab appropriate and reported in the manuscript?	Not Applicable	
If a study is subject to dual use research of concern regulations, is the name of the **authority granting approval and reference number** for the regulatory approval provided in the manuscript?	Not Applicable	

Reporting

The MDAR framework recommends adoption of discipline-specific guidelines, established and endorsed through community initiatives. Journals have their own policy about requiring specific guidelines and recommendations to complement MDAR.

Adherence to community standards	Information included in the manuscript?	In which section is the information available? (Reagents and Tools Table, Materials and Methods, Figures, Data Availability Section)
State if relevant guidelines or checklists (e.g., **ICMJE, MIBBI, ARRIVE, PRISMA**) have been followed or provided.	Not Applicable	
For **tumor marker prognostic studies,** we recommend that you follow the **REMARK** reporting guidelines (see link list at top right). See author guidelines, under 'Reporting Guidelines'. Please confirm you have followed these guidelines.	Not Applicable	
For **phase II and III randomized controlled trials**, please refer to the **CONSORT** flow diagram (see link list at top right) and submit the CONSORT checklist (see link list at top right) with your submission. See author guidelines, under 'Reporting Guidelines'. Please confirm you have submitted this list.	Not Applicable	

Data Availability

Data availability	Information included in the manuscript?	In which section is the information available? (Reagents and Tools Table, Materials and Methods, Figures, Data Availability Section)
Have **primary datasets** been deposited according to the journal's guidelines (see 'Data Deposition' section) and the respective accession numbers provided in the Data Availability Section?	Yes	Figure legends, Data availability
Were **human clinical and genomic datasets** deposited in a public access-controlled repository in accordance to ethical obligations to the patients and to the applicable consent agreement?	Not Applicable	
Are **computational models** that are central and integral to a study available without restrictions in a machine-readable form? Were the relevant accession numbers or links provided?	Not Applicable	
If publicly available data were reused, provide the respective **data citations in the reference list**.	Yes	Materials and Methods
